# Palladium-Catalyzed Carbonylative Cyclization of 1-Alkynyl-2-iodo-d-glucal

**DOI:** 10.1021/acs.orglett.4c03337

**Published:** 2024-09-30

**Authors:** Milene M. Hornink, Giuseppe E. Figlino, Mônica F. Z. J. Toledo, Daniel C. Pimenta, Hélio A. Stefani

**Affiliations:** † Faculdade de Ciências Farmacêuticas, Departamento de Farmácia, Universidade de São Paulo, São Paulo, SP 05508-220, Brazil; ‡ Centro Universitário São Camilo, São Paulo, SP 05585-000, Brazil; § Instituto Butantan, São Paulo, SP 05585-000, Brazil

## Abstract

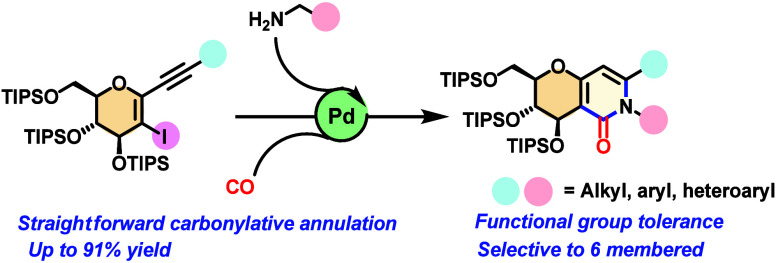

Cascade reactions are important synthetic tools for the synthesis of heterocyclic molecules, particularly those catalyzed by palladium. Herein, we report a palladium-catalyzed aminocarbonylative cyclization of new 1-alkynyl-2-iodo-d-glucals, which undergo a tandem carbonylative cyclization in the presence of various amine nucleophiles. A broad range of aromatic and aliphatic amines were applied as coupling partners, resulting in the selective and high-yield synthesis of glycosides fused to pyridinones. A plausible mechanism is proposed, proceeding via a tandem palladium aminocarbonylation followed by a palladium-catalyzed *endo-dig* cyclization.

Glycosides make up a class of renewable compounds found in nature, widely used as building blocks in organic synthesis.^1^ C-Glycosides feature an aglycon moiety or other glycoside linked through a C–C bond. They have garnered a significant amount of attention due to their potential applications as therapeutic agents, demonstrating enzymatic and chemical resistance to hydrolysis.^2^ Various approaches have been developed for the synthesis of C-glycosides,^3^ including C-oligosaccharides^4^ and C-aryl-glycosides.^5^ Several fused C-glycosides occur naturally, particularly those fused to ethers,^6^ such as marine neurotoxin neodysiherbaine A.^7^ Bergenin exhibits multiple pharmacological properties, including antihepatotoxic, antiulcerogenic, anti-HIV, antifungal, hepatoprotective, antiarrhythmic, and neuroprotective activities.^8^ Isatisin is a nitrogen heterocycle fused glycoside that shows anti-HIV activity (Scheme 1A).^9^

**Scheme 1:**
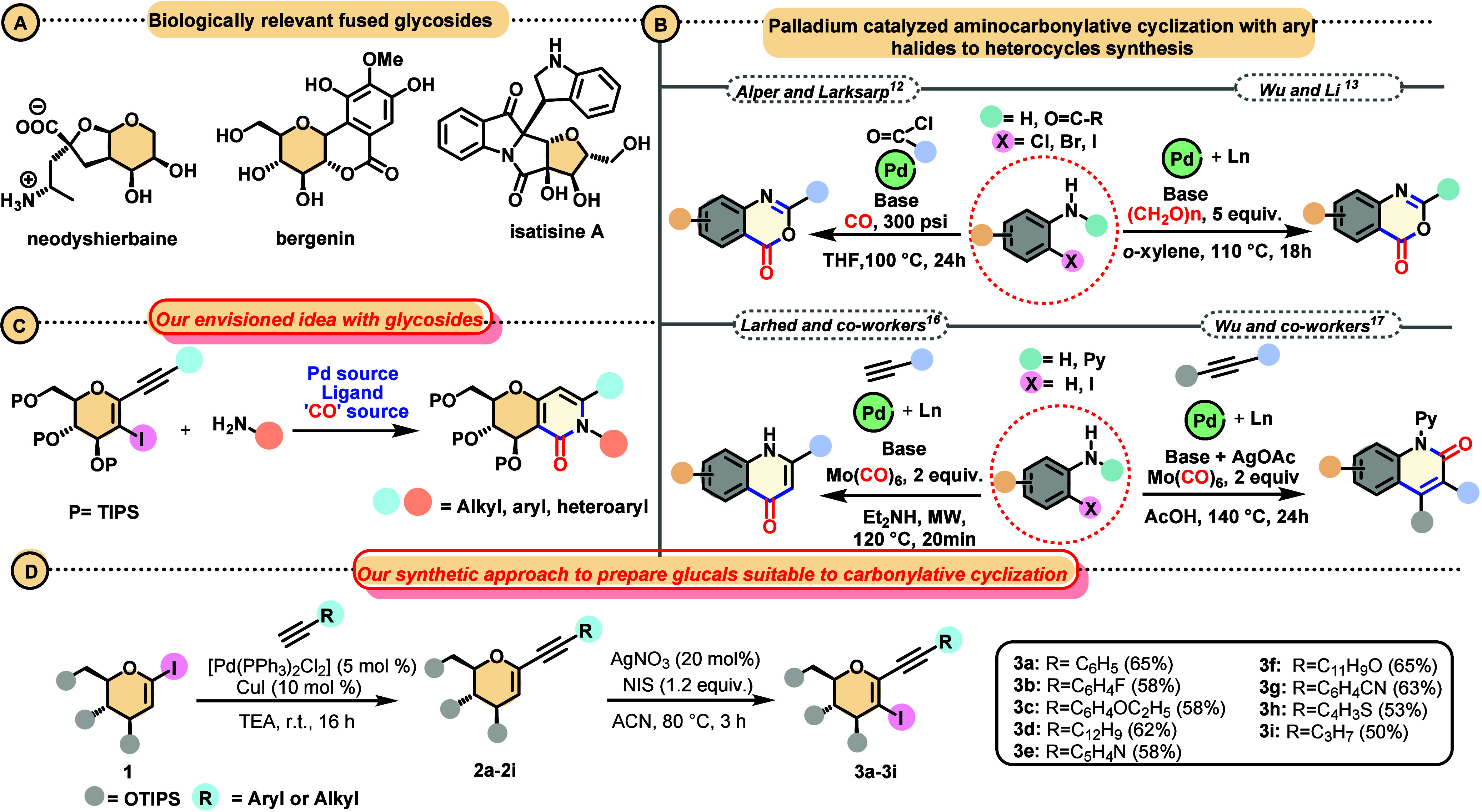
Background and Synthetic Approach for This Study

Cascade reactions are recognized as an important strategy for synthesizing organic molecules and as a sustainable tool, because a cascade approach-based synthesis avoids multiple steps, workups, and purifications.^10^ In this context, palladium-catalyzed cyclization reactions are a common synthetic tool, especially to construct heterocycles. They can be efficiently synthezized by a combination of carbonylative processes followed by intramolecular cyclization reactions.^11^ A survey of the literature revealed that palladium-catalyzed carbonylative cyclization was applied to the preparation of 2-substituted benzoxazinones, and different sources of carbonyl were used (Scheme 1B). In 2009, Alper and Larksar developed an approach based on a two-step process in which the starting 2-bromoaniline reacts with the acyl chloride and then the corresponding amide product undergoes oxidative addition in the catalytic cycle (Scheme 1B).^12^ Recently, Wu and Li demonstrated the direct use of a 2-bromoarylamide and applied formaldehyde as a “CO” source.^13^ Also, Larhed and co-workers applied a 2-iodophenol in a reaction with cyanoamide as a nucleophile to synthesize 2-aminobenzoxazinones; in this case, the CO source was Mo(CO)_6_.^14^

The quinolone nucleus can be prepared through palladium-catalyzed annulations, applying terminal alkynes as coupling partners with 2-iodoanilines. Ponomaryov reported the palladium-catalyzed preparation of 4-quinolones by applying high-pressure CO gas, 2-iodoanilines, and arylacetylenes.^15^ Later, approaches in which Mo(CO)_6_ was applied became more common, and Larhed and co-workers demonstrated the reaction of substituted 2-iodoanilines with acetylenes under microwave irradiation.^16^ Wu and co-workers developed a palladium-promoted cyclization to synthesize 2-quinolones, starting from pyridine-substituted anilines, which avoided the use of haloanilines (Scheme 1B).^17^ Other groups also studied different reagents and conditions to prepare quinolones,^18^ quinoxalinones,^19^ and quinazolinones.^20^

In this study, we report a strategy for synthesizing glucals fused to pyridinones through a palladium-catalyzed aminocarbonylative cyclization of 1-alkynyl-2-iodo-d-glucals and amines using Mo(CO)_6_ as a solid source of CO (Scheme 1C). There has been no previous study of glucal derivatives engaged in carbonylative cyclizations. Our initial efforts focused on synthesizing a suitable substrate, 1-alkynyl-2-iodo-d-glucals (**3a**–**i**) (Scheme 1D), envisioned to undergo the proposed tandem carbonylative cyclization in the presence of a palladium catalyst. As shown in Scheme 1D, the first step involved the palladium-catalyzed Sonogashira reaction with 1-iodoglucal, yielding large amounts of alkynylated products **2a**–**i**. We then performed a selective 2-iodination^21^ to prepare compounds **3a**–**i** in good yields (for more details, see sections 2.1 and 2.2 of the Supporting Information). In our exploratory experiments, we employed compound **3a**, containing an electron-neutral phenyl acetylene derivative, along with aniline as our coupling partner in the presence of PdCl_2_, DIPEA (*N*,*N*-diisopropylethylamine) as the base, and Mo(CO)_6_ as the “CO” source, and the reaction mixture was heated in oil bath to 80 °C for 16 h. This condition leads directly to target six-membered product **4a** in a selective fashion in an isolated yield of 26%; unreacted starting material **3** was recovered. The structure of the final pyridinone product was confirmed by heteronuclear single-quantum coherence (HSQC) and heteronuclear multiple-bond coherence (HMBC) nuclear magnetic resonance (NMR) experiments (section 4 of the Supporting Information).

On the basis of this positive result, we performed further optimization steps to seek other reagents and conditions. First, we evaluated the palladium source; the use of Pd(OAc)_2_, without the addition of a ligand, led to a slight increase in the yield. The use of a Pd(0) species such as Pd(dba)_2_ led to the observation of only traces of products, with the observation of the unreacted starting material (see entries 1–3 of Table 1 of the Supporting Information). When we added a phosphine such as DEPphos, an increase in the isolated yield was observed. The use of TEA (triethylamine) as an organic base decreased the isolated yield; the same result was observed with K_2_CO_3_. As the isolated yield was not efficiently affected by these changes, we decided to double the number of equivalents of aniline to 2.4, which increased the yield (see entry 9 of Table 1 of the Supporting Information). Toluene as a solvent led to a decrease in the yield; however, when acetonitrile was used, an increase in the yield was achieved (see entries 10 and 11 of Table 1 of the Supporting Information). The increase in the reaction time was beneficial to the final isolated yield (91%). For full optimization details, see section 2.3 of the Supporting Information. Control experiments were performed to check the influence of each reaction component. The Mo(CO)_6_-promoted cyclization is known from the literature.^22^ For entry 2 of Table 1, the reaction was performed with no palladium, and only the starting material was recovered. Without the phosphine ligand, a moderate yield was achieved (entry 3); in this case, the presence of the phosphine is important to enable product formation in high yield. In entry 4, the base decomposition of CHCl_3_ was applied as an ex situ methodology to generate CO in a two-chamber system but a very poor yield was obtained. To verify if the excess aniline used would act as a base in the catalytic process, an experiment was conducted in the absence of a base, but only traces of products were observed (entry 5). Finally, when 1,4-dioxane was used as the solvent (entry 6), a considerable decrease in the yield was observed, indicating that when acetonitrile is used as the solvent it may act as a ligand.

**Table 1:**
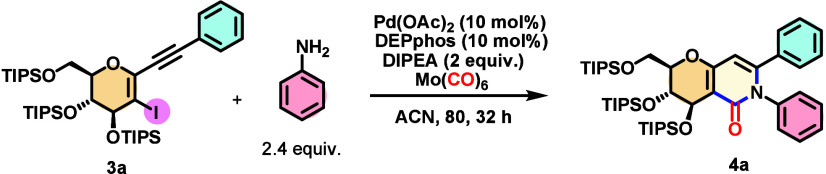
Control Experiments

entry	deviation from the “standard conditions”	yield (%)^a^
1	none	91
2	no Pd(OAc)_2_	no reaction
3	no DEPphos	46
4	no Mo(CO)_6_	10^b^
5	do DIPEA	trace
6	1,4-dioxane as the solvent	64

a Isolated yields.

b The reaction was conducted using a system of CHCl_3_ (5 equiv) and KOH (10 equiv) in toluene as the “CO” source in a two-pot process.

We then proceeded to evaluate the generality of the reaction scope, as shown in Scheme 2. First, we chose **3a** as the starting material, containing an electron-neutral phenyl substituent, and different anilines were applied. We performed experiments with electron-neutral aniline, electron-rich 4-ethoxyaniline, and sterically demanding 2,3-trimethylaniline and 2-naphthylaniline, giving high yields of products **4a**–**d** in the range of 80–91%. When 4-fluoroaniline was applied, the yield remained increased for compound **4e** (73%). A reaction with electron-poor 4-cyanoaniline did not take place, and only the starting material was recovered. When electron-poor 2-aminopyridine was evaluated, a poor yield of product **4g** of only 32% was obtained.

**Scheme 2:**
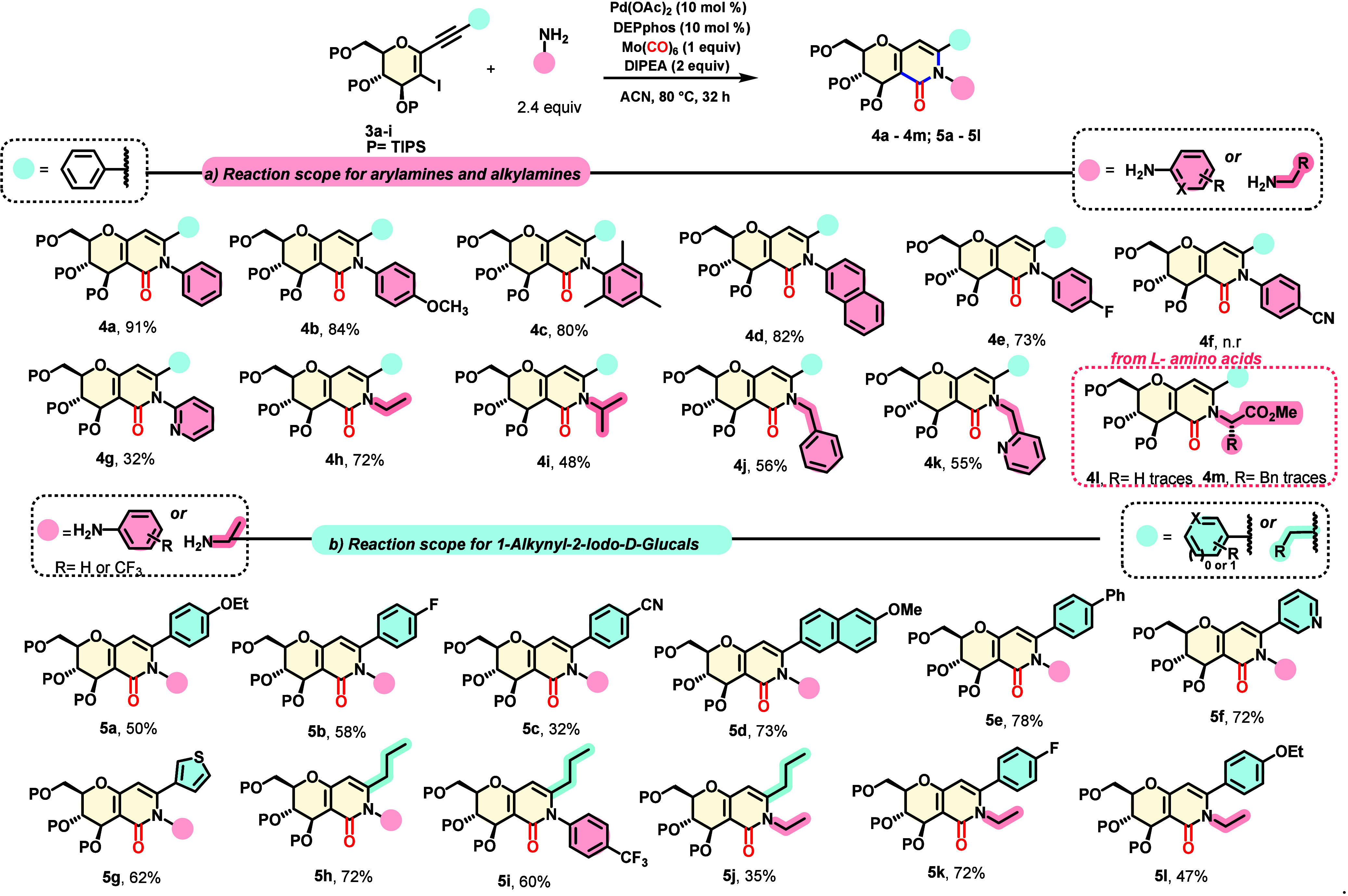
Substrate Scope

Next, we evaluated alkylamines as coupling partners. Ethylamine provided **4h** in good yield (72%), and isopropylamine produced a moderate yield of 48%. Benzylamine and 2-picolylamine provided good yields of **4j** and **4k**, respectively. l-Amino acid esters were also evaluated in the cyclization process; only the starting material was recovered (Scheme 2, **4l** and **4m**). Next, we explored different groups attached to the alkyne by fixing aniline as the coupling partner. Moderate yields were obtained with electron-rich aromatic acetylenes such as 4-OEt (**5a**, 50%) and its halogenated analogue 4-F (**5b**, 58%). When an electron-poor aromatic acetylene substituted with a 4-CN group was applied, the yield of the desired product **5c** was decreased to 32%. On the contrary, electron-rich naphthyl and biphenyl groups attached to the acetylene provided higher yields of **5d** and **5e**, respectively. Heterocyclic moieties attached to the alkyne were also investigated. Interestingly, when electron-poor pyridine (**5f**) was tested, an increased yield of 72% was obtained, whereas with electron-rich thiophenes (**5g**), a moderate yield of 62% was achieved. Also, the reactivity of alkyl groups attached to the alkyne moiety was explored, with compounds **5h** and **5i** isolated in good yields of 72% and 60%, respectively, when aniline and 4-trifluoromethylaniline were applied as the coupling partners. The use of an alkylamine, such as ethylamine, resulted in a poor isolated yield of only 35%. Mixed examples of the substituted acetylenes were prepared by coupling 4-fluorophenyl-substituted and 4-ethoxy-substituted derivatives with ethylamine, producing good yields of 72% and 47% for compounds **5k** and **5l**, respectively.

Subsequently, we performed some derivatization of the cyclization products and reaction scale-up. When we performed the carbonylative cyclization reaction on a 1 mmol scale, a high yield of the desired compound of 74% was isolated, as shown in Scheme 3a. Then, the triisopropylsilyl (TIPS) protecting group was removed by the use of a tetrabutylammonium fluoride (TBAF) solution in tetrahydrofuran (THF), and trihydroxylated products **6a** and **6b** were isolated in high yields [85% and 81%, respectively (Scheme 3b)]. To test the extension of the reaction with secondary amines, we performed aminocarbonylation with morpholine, but after 32 h, only traces of products were isolated (Scheme 3c). Next, we decided to use the 2-bromide derivative to evaluate whether it reacts as the iodide, but in this experiment, only the starting material was isolated (Scheme 3d).

**Scheme 3:**
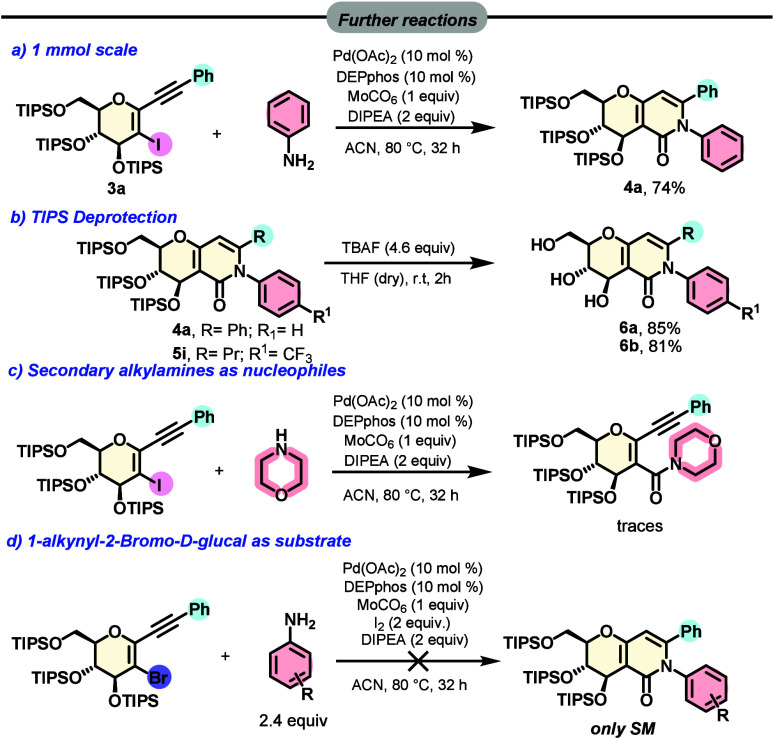
Further Reactions

The proposed reaction mechanism (Scheme 4) starts with reduction of Pd(II) to active species Pd(0), and then oxidative addition furnishes intermediate **I**. On the basis of the literature, CO insertion and the formation of intermediate **II** are the next steps;^19^ then, nucleophilic attack forms intermediate **III**, and reductive elimination leads to the formation of **IV**. In this step, the palladium may be coordinated to the alkyne moiety and nucleophilic attack of the amide nitrogen is facilitated due to activation of the triple bond by Pd(II).^23^ The attack is an *endo-dig* cyclization, although an *exo-dig* cyclization is also favorable in terms of Baldwin rules;^24^ however, this product was not observed. Intermediate **V**, the σ-vinylpalladium complex, undergoes a protodepalladation reaction to give product **4**.^25^

**Scheme 4:**
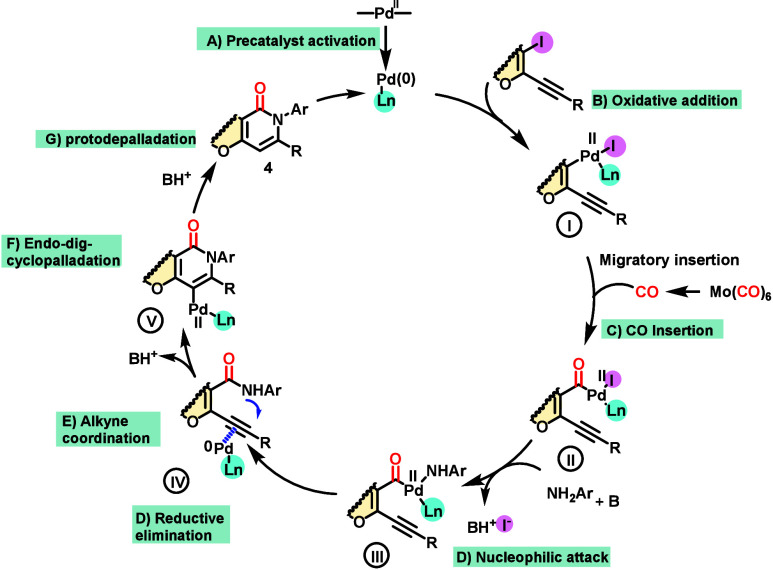
Proposed Reaction Mechanism

In conclusion, we present a novel palladium-catalyzed tandem aminocarbonylative cyclization of 1-alkynyl-2-iodoglucals for the synthesis of new pyridinone derivatives via selective *endo-dig* cyclization. This method effectively engages a variety of aromatic, heterocyclic, and alkyl acetylenes with readily accessible amine nucleophiles, enabling the straightforward incorporation of a carbonyl functionality. The process exhibited high selectivity, yielding six-membered rings as the sole products. We successfully synthesized 24 new glucal-fused pyridinones via this elegant sequential transformation, demonstrating the method’s efficiency and versatility.

## Data Availability

The data underlying this study are available in the published article and its Supporting Information.

## Supporting Information Available

FAIR data, including the primary NMR FID files, for compounds **2c**–**h**, **3a**–**i**, **4a**–**e**, **4g**–**k**, **5a**–**l**, **6a**, and **6b** (ZIP)
Experimental procedures, compound characterization data (NMR, IR, HRMS, and [α]_D_^20^), and copies of ^1^H and ^13^C NMR spectra (PDF)
