# Supplementary material for: Palladium-Catalyzed Carbonylative Cyclization of 1-Alkynyl-2-iodo-d-glucal
Source: Org Lett. 2024 Sep 30;26(40):8621–5. doi: 10.1021/acs.orglett.4c03337 (PMC11474949; doi:10.1021/acs.orglett.4c03337)
Supplement: Supplementary file 2 — ol4c03337_si_002.pdf [file ol4c03337_si_002.pdf]

Palladium-Catalyzed Carbonylative Cyclization of 1-Alkynyl-2-Iodo-D-Glucal

Milene M. Hornink,[a] Giuseppe E. Figlino,[b] Monica F. Z. J. Toledo,[a] Daniel C. Pimenta,[c] Hélio A Stefani*[a]

[a]Faculdade de Ciências Farmacêuticas, Departamento de Farmácia, Universidade de São Paulo, São Paulo SP - Brazil. [b]Centro Universitário São Camilo, [c]Instituto Butantan, São Paulo - SP, Brazil.

***Corresponding Author**: hstefani@usp.br

Table of Contents

1. General Information...2

2. Experimental Procedures and Characterization Data...2

2.1 General procedure for the synthesis of 1-alkynyl-glucals (2) 2

2.2 General procedure for the synthesis of 2-iodo-1-alkinyl-glucals (3) 5

2.3 Optimization studies...9

2.4 General procedure for Carbonylative cyclization reaction (4 and 5) 11

2.5 Procedure for Deprotection (6) 22

3. NMR (^{1}H, ^{13}C) Spectra of Products...24

4. NMR (HMBC, HSQC) Spectra of Products...63

1. General Information

All reagents were purchased from Sigma-Aldrich, Alfa Aesar, Acros Organics, Oakwood or Fluorochem. When they were not a HPLC-grade solvents, they were purified by distillation. Other solvents, like DIPEA was also dried over CaH_2. Thin Layer Chromatography was carried out using g Merck TLC 60 F254 silica gel plates and visualized under UV light (254 nm) and stained with acidic vanillin solution. Flash column chromatography was performed using silica gel with a pore size of 60 Å, 230-400 Mesh (Sigma Aldrich, cat.# 22,719-6). Nuclear magnetic resonance (NMR) spectra were recorded in $CDCl_3$, $(CD_3)_2CO$) or DMSO-*d6* using a Bruker DPX 300 instrument (^{1}H at 300 MHz, ^{13}C at 75 MHz). Chemical shifts, δ, are reported in parts per million (ppm) and are referenced to the residual solvent. ^{1}H peaks are quoted to the nearest 0.01 Hz and ^{13}C peaks are quoted to the nearest 0.1 Hz. The abbreviation utilized to report the peaks are: s (singlet), d (doublet), t (triplet), dd (doublet of doublets) m (multiplet). High-resolution mass spectra (HRMS) were recorded on a Shimadzu ESI-TOF mass spectrometer. FTIR data were obtained using an Agilent Technologies Cary 630. Optical rotations were measured at 20 °C by using an Anton Paar MCP200 Polarimeter.

2. Experimental Procedures and Characterization Data

The *O*-TIPS protection of D-glucal and its subsequent C1 iodination (**1**) was performed using protocols reported in literature.[1]

2.1 General procedure for the synthesis of 1-alkynyl-glucals (2)

1-alkynyl-glucals were prepared according to previous published methods.[1d]

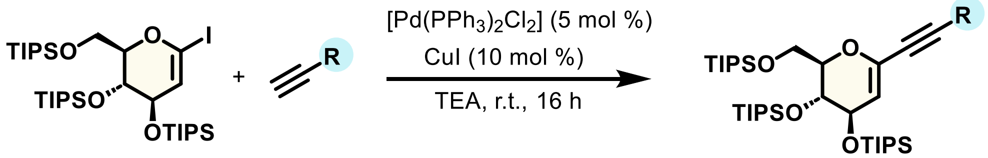

$[Pd(PPh_3)_2Cl_2]$ (7.0 mg, 5 mol %) and CuI (3.8 mg, 10 mol %) were added to a solution of 1-iodoglucal (0.2 mmol, 1.0 equiv) and acetylene (0.48 mmol, 2.4 equiv) in Et_3N (6 mL). the reaction was stirred at room temperature for 16 hours. The solvent was

[1] a) Friesen, R.; Loo, R. W. *J. Org. Chem.* **1991**, *56*, 4821. b) Friesen, R. W.; Sturino, C. F.; Daljeet, A. K.; Kolaczewska *J. Org. Chem.* **1991**, *56*, 1944 .c) Potuzak, J. S.; Tan, D. S. *Tetrahedron Lett*. **2004**, *45*, 1797. d) Koester, D. C.; Werz, D. B. *Beilstein J. Org. Chem*. **2012**, *8*, 675. e) Parkan, K.; Pohl, R.; Kotora, M. *Chem. Eur. J.* **2014**, *20*, 4414. f) Liu, M.; Niu, Y.; Wu, Y-F.; Ye, X-S. *Org. Lett.* **2016**, *18*, 1836. g) Linker, T. Schanzenbach, D.; Elamparuthi, E.; Sommermann, T.; Fudickar, W.; Gyo llai, V.; Somsa k, L.; Demuth, W.; Schmittel M. *J. Am. Chem. Soc.* **2008**, 130, 16003. h) Zhang, S.; Niu, S-Z.; Y-H.; Ye, X-S. *Org. Let.* **2017,** 19, 3608.

removed under reduced pressure. The 1-alkynyl-glucals were purified by column chromatography on silica gel flash.

(((2*R*,3*R*,4*R*)-6-(phenylethynyl)-2-(((triisopropylsilyl)oxy)methyl)-3,4-dihydro-2*H*-pyran-3,4-diyl)bis(oxy))bis(triisopropylsilane) (2a)

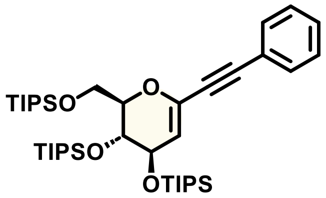

The product was obtained as a yellow oil (131.6 mg, 92 %). The spectral data are in accordance with the previously reported in the literature. [1d]

(((2*R*,3*R*,4*R*)-6-(phenylethynyl)-2-(((triisopropylsilyl)oxy)methyl)-3,4-dihydro-2*H*-pyran-3,4-diyl)bis(oxy))bis(triisopropylsilane) (2b)

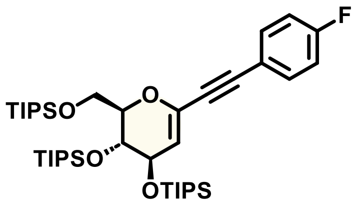

The product was obtained as a yellow oil (134.9 mg, 92 %). The spectral data are in accordance with the previously reported in the literature. [1d]

(((2*R*,3*R*,4*R*)-6-((4-ethoxyphenyl)ethynyl)-2-(((triisopropylsilyl)oxy)methyl)-3,4-dihydro-2*H*-pyran-3,4-diyl)bis(oxy))bis(triisopropylsilane) (2c)

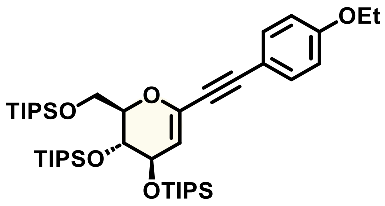

The product was obtained as a yellow oil (135.16 mg, 89 %). Eluent: EtOAc/hexane = 1/99 to 5/95. **^{1}H NMR** (300 MHz, $CDCl_3$) δ 7.42 (d, J = 8.4 Hz, 2H), 6.82 (d, J = 8.4 Hz, 2H), 5.31 (d, J = 4.6 Hz, 1H), 4.38 – 4.35 (m, 1H), 4.13 – 4.00 (m, 5H), 3.93 (dd, J = 11.1 Hz, 4.9 Hz, 1H), 1.41 (t, J = 7.0 Hz, 3H), 1.08 (m, 63H). **^{13}C NMR** (75 MHz, $CDCl_3$) δ 159.4, 136.5, 133.4 (2C), 114.5 (2C), 114.4, 106.6, 87.4, 83.9, 81.6, 69.7, 66.2, 63.6, 61.9, 29.9, 18.3 – 18.2 (18C), 14.9, 12.7 (3C), 12.5 (3C), 12.2 (3C). **HRMS (ESI)** *m/z*: $[M + H]^+$ Calcd for $C_{43}H_{79}O_5Si_3$ 759.5235; Found 759.5235.

(((2*R*,3*R*,4*R*)-6-([1,1'-biphenyl]-4-ylethynyl)-2-(((triisopropylsilyl)oxy)methyl)-3,4-dihydro-2*H*-pyran-3,4-diyl)bis(oxy))bis(triisopropylsilane) (2d)

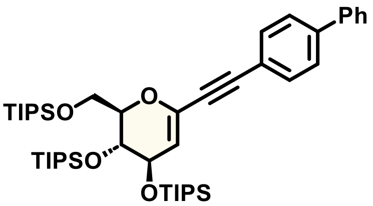

The product was obtained as a yellow semisolid (134.5 mg, 85 %). Eluent: DCM/hexane = 5/95 to 10/90. **^{1}H NMR** (300 MHz, $CDCl_3$) δ 7.62 – 7.56 (m, 6H), 7.49 – 7.42 (m, 2H), 7.38 – 7.33(m, 1H), 5.37 (dd, *J* = 5.3, 1.4 Hz, 1H), 4.41 – 4.37 (m, 1H), 4.15 – 4.06 (m, 3H), 3.94 (dd, *J* = 11.2 Hz, 4.7 Hz, 1H), 1.10 (m, 63H). **^{13}C NMR** (75 MHz, $CDCl_3$) δ 141.4, 140.5, 136.5, 132.4 (2C), 129.0 (2C), 127.8, 127.2 (2C), 127.1 (2C), 121.5, 107.3, 87.2, 86.0, 81.8, 69.7, 66.2, 61.9, 18.4-18.2 (18C), 12.7 (3C), 12.6 (3C), 12.3 (3C). **HRMS (ESI)** *m/z*: $[M + Na]^+$ Calcd for $C_{47}H_{78}O_4Si_3Na$ 813.5106; Found 813.5115.

3-(((2*R*,3*R*,4*R*)-3,4-bis((triisopropylsilyl)oxy)-2-(((triisopropylsilyl)oxy)methyl)-3,4-dihydro-2*H*-pyran-6-yl)ethynyl)pyridine (2e)

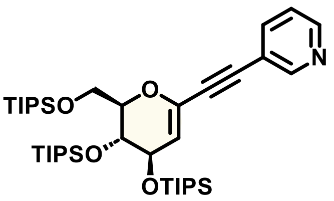

The product was obtained as a yellow oil (104.7 mg, 85 %). Eluente: EtOAc/hexane = 5/95. **^{1}H NMR** (300 MHz, $CDCl_3$) δ 8.73 (s, 1H), 8.56 (d, *J* = 5.2 Hz, 1H), 7.90 – 7.86 (m, 1H), 7.39 – 7.34 (m, 1H), 5.41 (dd, *J* = 5.3, 1.6 Hz, 1H), 4.40 – 4.36 (m, 1H), 4.13 – 4.04 (m, 3H), 3.89 (dd, *J* = 11.1, 4.3 Hz, 1H), 1.08 (m, 63H). **^{13}C NMR** (75 MHz, $CDCl_3$) δ 151.0, 147.4, 140.2, 135.8, 123.7, 120.6, 108.7, 89.5, 83.1, 81.2, 69.6, 65.9, 61.8, 18.3-18.2 (18C), 12.7 (3C), 12.5 (3C), 12.2 (3C). **HRMS (ESI)** *m/z*: $[M + H]^+$ Calcd for $C_{40}H_{74}NO_4Si_3$ 716.4926; Found 716.4909.

(((2*R*,3*R*,4*R*)-6-([1,1'-biphenyl]-4-ylethynyl)-2-(((triisopropylsilyl)oxy)methyl)-3,4-dihydro-2*H*-pyran-3,4-diyl)bis(oxy))bis(triisopropylsilane) (2f)

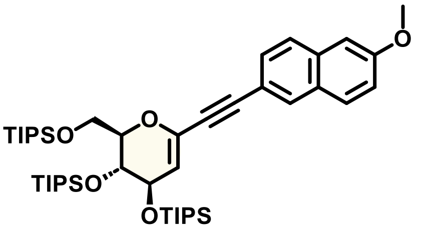

The product was obtained as a yellow oil (132.6 mg, 85 %). Eluente: DCM/hexane = 5/95 to 25/75. **^{1}H NMR** (300 MHz, $CDCl_3$) δ 7.95 (d, *J* = 1.6 Hz, 1H), 7.70-7.64 (m, 2H), 7.50 (dd, *J* = 8.5 Hz, 1.7 Hz, 1H), 7.15 (dd, *J* = 8.8 Hz, 2.5 Hz, 1H), 7.10 (d, *J* = 2.5 Hz, 1H), 5.38 (dd, *J* = 5.4 Hz, 1.7 Hz, 1H), 4.41-4.37 (m, 1H), 4.15-4.06 (m, 3H), 3.98-3.92 (m, 4H), 1.15-1.10 (m, 63H). **^{13}C NMR** (75 MHz, $CDCl_3$) δ 158.6, 136.5, 134.5, 131.8, 129.5, 129.1, 128.5, 126.8, 119.5, 117.5, 107.0, 106.0, 87.9, 84.9, 81.7, 69.7, 66.2, 61.9, 55.5, 18.3-18.2 (18C), 12.7 (3C), 12.5 (3C), 12.3 (3C). **HRMS (ESI)** *m/z*: $[M + H]^+$ Calcd for $C_{46}H_{79}O_5Si_3$ 795.5235; Found 795.5231.

4-(((2*R*,3*R*,4*R*)-3,4-bis((triisopropylsilyl)oxy)-2-(((triisopropylsilyl)oxy)methyl)-3,4-dihydro-2*H*-pyran-6-yl)ethynyl)benzonitrile (2g)

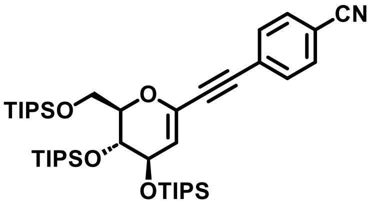

The product was obtained as a yellow oil (111 mg, 75 %). Eluente: DCM/hexane 20/80 to 50/50. **^{1}H NMR** (300 MHz, $CDCl_3$) δ 8.60 (d, *J* = 8.2 Hz, 2H), 7.55 (d, *J* = 8.3 Hz, 2H), 5.40 (d, *J* = 5.2 Hz, 1H), 4.39-4.35 (m, 1H), 4.10-4.03 (m, 3H), 3.88 (dd, *J* = 11.2 Hz, 4.4 Hz, 1H), 1.07 (m, 63H). **^{13}C NMR** (75 MHz, $CDCl_3$) δ 135.8, 132.3 (2C), 132.0 (2C), 127.5, 118.5, 122.0, 108.8, 89.4, 85.5, 81.9, 69.6, 65.9, 61.8, 18.3-18.1 (18C), 12.6 (3C), 12.5 (3C), 12.2 (3C). **HRMS (ESI)** *m/z*: $[M + K]^+$ Calcd for $C_{42}H_{73}NO_4Si_3K$ 778.4484; Found 778.4483.

(((2*R*,3*R*,4*R*)-6-(thiophen-2-ylethynyl)-2-(((triisopropylsilyl)oxy)methyl)-3,4-dihydro-2*H*-pyran-3,4-diyl)bis(oxy))bis(triisopropylsilane) (2h)

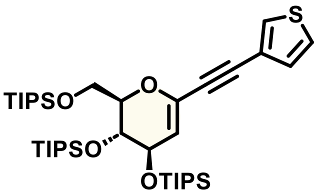

The product was obtained as a pale yellow oil (102.1 mg, 88 %). Eluente: DCM/hexane 5/95 to 15/85. **^{1}H NMR** (300 MHz, $CDCl_3$) δ 7.52 (dd, *J* = 2.9 Hz, 1.0 Hz, 1H), 7.27 (t, *J* = 2.6 Hz, 1H), 7.17 (dd, *J* = 5.2 Hz, 1.0 Hz, 1H), 5.33 (dd, *J* = 5.3, 1.3 Hz, 1H), 4.39-4.35 (m, 1H), 4.14-4.04 (m, 3H), 3.93 (dd, *J* = 11.4, 4.8 Hz, 1H), 1.09 (m, 63H). **^{13}C NMR** (75 MHz, $CDCl_3$) δ 136.3, 130.0, 129.6, 125.3, 1217.7, 107.1, 84.8, 82.5, 81.7, 69.7, 66.1, 61.8, 18.3-18.2 (18C), 12.7 (3C), 1254 (3C), 12.2 (3C). **HRMS (ESI)** *m/z*: $[M + K]^+$ Calcd for $C_{39}H_{72}O_4SSi_3K$ 759,4096; Found 759.4096.

(((2*R*,3*R*,4*R*)-6-(pent-1-yn-1-yl)-2-(((triisopropylsilyl)oxy)methyl)-3,4-dihydro-2*H*-pyran-3,4-diyl)bis(oxy))bis(triisopropylsilane) (2i)

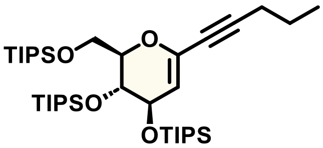

The product was obtained as a colorless oil (111.7 mg, 82 %). The spectral data are in accordance with the previously reported in the literature. [1d]

2.2 General procedure for the synthesis of 2-iodo-1-alkynyl-glucals (3) [2]

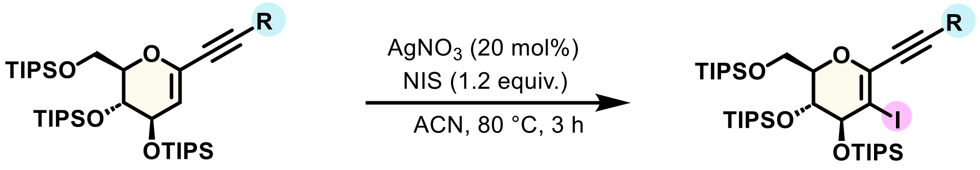

2a-i **3a-i**

$AgNO_3$ (0.04 mmol, 6.9 mg, 20 mol %) and NIS (0.24 mmol, 54 mg, 1.2 equiv) were added to a solution of the 1-alkynyl glucal (0.2 mmol, 1.0 equiv). The reaction was stirred at 80 °C in oil bath for 3 hours. The reaction was filtered of in a pad of Celite and thoroughly rinsed with acetone. The solvent was removed under reduced pressure. The crude product was purified by column chromatography on silica gel flash.

(((2*R*,3*R*,4*S*)-5-iodo-6-(phenylethynyl)-2-(((triisopropylsilyl)oxy)methyl)-3,4-dihydro-2*H*-pyran-3,4-diyl)bis(oxy))bis(triisopropylsilane) (3a)

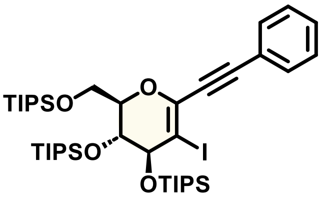

The product was obtained as a pale yellow oil (109.3 mg, 65 %). Eluent: hexane/DCM = 100/0 to 95/5. **^{1}H NMR** (300 MHz, $CDCl_3$) δ 7.56 – 7.53 (m, 2H), 7.34 – 7.32 (m. 3H), 4.48 – 4.43 (m, 1H), 4.32 – 4.31 (m, 1H), 4.16 – 4.15 (m, 1H), 4.10 (dd, *J* = 11.5 Hz, 7.9 Hz, 1H), 3.89 (dd, *J* = 11.5 Hz, 3.9 Hz, 1H), 1.14 – 1.06 (m, 63H). **^{13}C NMR** (75 MHz, $CDCl_3$) δ 138.8, 132.1 (2C), 129.1, 128.4 (2C), 122.3, 91.2, 87.1, 82.1, 78.8, 75.3, 70.9, 62.2, 18.6 – 18.2 (18C), 13.2 (3C), 12.7 (3C), 12.3 (3C). **HRMS (ESI)** *m/z*: $[M + Na]^+$ Calcd for $C_{41}H_{73}IO_4Si_3Na$ 863.3759; Found 863.3774.

(((2*R*,3*R*,4*S*)-6-((4-fluorophenyl)ethynyl)-5-iodo-2-(((triisopropylsilyl)oxy)methyl)-3,4-dihydro-2*H*-pyran-3,4-diyl)bis(oxy))bis(triisopropylsilane) (3b)

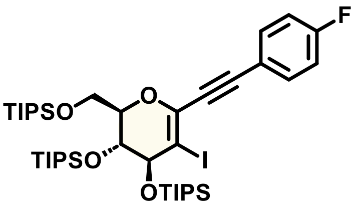

The product was obtained as a yellow oil (99.7 mg, 58 %). Eluent: hexane/DCM = 100/0 to 95/5. **^{1}H NMR** (300 MHz, $(CD_3\ )_2$ CO) δ 7.63 – 7.58 (m, 2H), 7.25 – 7.19 (m. 2H), 4.54 – 4.50 (m, 1 H), 4.41 – 4.39 (m, 1 H), 4.29 – 4.27 (m, 1 H), 4.21 (dd, *J* = 11.6, 8.2 Hz, 1 H), (dd, *J* = 11.6, 3.4 Hz, 1 H), 1.20 – 1.10 (m, 63H). **^{13}C NMR** (75 MHz, $(CD_3\ )_2$ CO) δ 164.0 (d, *J* = 247 Hz, C-F), 139.4, 134.8 (d, *J* = 8.7 Hz, C-F), 118.9 (d, *J* = 3.3 Hz, C-F), 116.8 (d, *J* = 21 Hz, C-F), 90.6, 87.4, 82.9, 79.1,

---

[2] Dharuman, S.; Vankar, Y.D. *Org. Lett.* **2014**, 16, 4, 1172–1175.

76.0, 71.5, 62.9, 18.9 – 18.4 (18 C), 13.8 (3 C), 13.3 (3 C), 12.9 (3 C). **HRMS (ESI)** *m/z*: $[M + Na]^+$ Calcd for $C_{41}H_{72}FIO_4Si_3Na$ 881.3665; found 881.3660.

(((2*R*,3*R*,4*S*)-6-((4-ethoxyphenyl)ethynyl)-5-iodo-2-(((triisopropylsilyl)oxy)methyl)-3,4-dihydro-2*H*-pyran-3,4-diyl)bis(oxy))bis(triisopropylsilane) (3c)

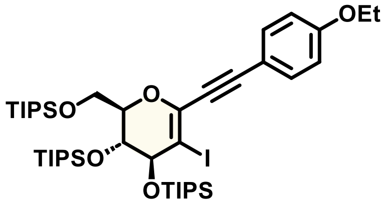

The product was obtained as a yellow oil (113 mg, 58%). Eluent: hexane/DCM = 95/5 to 90/10. **^{1}H NMR** (300 MHz, $CDCl_3$) δ 7.47 (d, *J* = 8.9 Hz, 2H), 6.83 (d, *J* = 8.8 Hz, 2H), 4.47-4.43 (m, 1 H), 4.33-4.31 (m, 1H), 4.17 – 4.15 (m, 1H), 4.04 (q, *J* = 7.0 Hz, 2H), 3.91 (dd, *J* = 11.4 Hz, 4.1 Hz, 1 H), 1.41 (d, *J* = 7.0 Hz, 3H), 1.14 – 1.06 (m, 63H). **^{13}C NMR** (75 MHz, $CDCl_3$) δ 159.8, 139.0, 133.6 (2C), 114.6 (2C), 114.1, 91.5, 85.9, 82.1, 78.1, 75.3, 70.9, 63.7, 62.3, 18.7 – 18.2 (18C), 14.9, 13.2 (3C), 12.7 (3C), 12.3 (3C). **HRMS (ESI)** *m/z*: $[M + H]^+$ Calcd for $C_{43}H_{78}IO_5Si_3$ 885.4202; Found 885.4245.

((((2*R*,3*R*,4*S*)-6-([1,1'-biphenyl]-4-ylethynyl)-5-iodo-2-(((triisopropylsilyl)oxy)methyl)-3,4-dihydro-2*H*-pyran-3,4-diyl)bis(oxy))bis(triisopropylsilane) (3d)

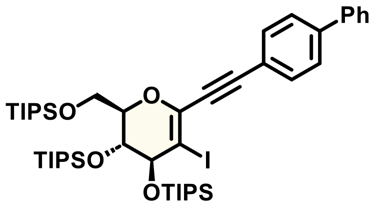

The product was obtained as a pale yellow semisolid (113.7 mg, 62 %). Eluent: hexane/DCM = 95/5 to 90/10. **^{1}H NMR** (300 MHz, $(CD_3)_2CO$) δ 7.74 – 7.63 (m, 6H), 7.52 – 7.47 (m, 2H), 7.42 – 7.40 (m, 1H), 4.57 – 4.52 (m, 1H), 4.43 – 4.43 (m, 1H), 4.29 – 4.28 (m, 1H), 4.23 (dd, *J* = 11.6 Hz, 8.2 Hz, 1H), 3.99 (dd, *J* = 11.5 Hz, 3.5 Hz, 1 H), 1.22-1.13 (m, 63H). **^{13}C NMR** (75 MHz, $CDCl_3$) δ 141.8, 140.4, 138.8, 133.1, 132.4 (2C), 129.0 (2C), 127.8, 127.28, 127.20 (2C), 127.1 (2C), 121.1, 91.1, 87.7, 82.1, 78.9, 75.3, 70.9, 62.2, 18.6 – 18.1 (18C), 13.1 (3C), 12.7 (3C), 12.3 (3C). **HRMS (ESI)** *m/z*: $[M + Na]^+$ Calcd for $C_{47}H_{77}IO_4Si_3Na$ 939.4066; Found 939.4072.

3-(((2*R*,3*R*,4*S*)-5-iodo-3,4-bis((triisopropylsilyl)oxy)-2-(((triisopropylsilyl)oxy)methyl)-3,4-dihydro-2*H*-pyran-6-yl)ethynyl)pyridine (3e)

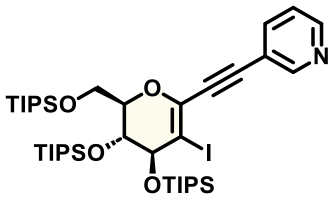

The product was obtained as a bege solid (113.7 mg, 62 %). Eluent: hexane/EtOAc = 95/5 to 90/10. mp. 50-52 °C. **^{1}H NMR** (300 MHz, $CDCl_3$) δ 8.78 (s, 1H), 8.56 (dd, *J* = 4.7 Hz, 1.0 Hz, 1H), 7.81 (dt, *J* = 7.9 Hz, 2.0 Hz, 1H), 7.29 –

7.25 (m, 1H), 4.49 – 4.45 (m, 1H), 4.33 – 4.32 (m, 1H), 4.17 – 4.16 (m, 1H), 4.11 (dd, *J* = 11.6 Hz, 8.1 Hz, 1H), 3.88 (dd, *J* = 11.5 Hz, 3.9 Hz, 1H), 1.14 – 1.06 (m, 63H). **^{13}C NMR** (75 MHz, $CDCl_3$) δ 152.6, 149.3, 138.8, 138.4, 123.1, 119.5, 90.1, 87.6, 82.2, 79.8, 75.2, 70.8, 62.2, 18.7 – 18.2 (18C), 13.2 (3C), 12.7 (3C), 12.3 (3C). **HRMS (ESI)** *m/z*: $[M + H]^+$ Calcd for $C_{40}H_{73}INO_4Si_3$ 842.3892; Found 842.3853.

(((2*R*,3*R*,4*S*)-5-iodo-6-((6-methoxynaphthalen-2-yl)ethynyl)-2-(((triisopropylsilyl) oxy)methyl)-3,4-dihydro-2*H*-pyran-3,4-diyl)bis(oxy))bis(triisopropylsilane) (3f)

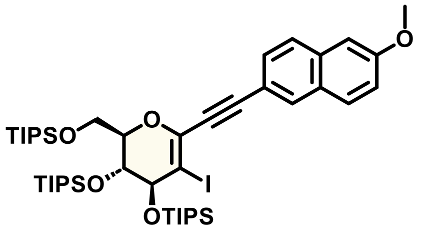

The product was obtained as a pale yellow oil (119.7 mg, 65%). Eluente: hexane/DCM = 90/10 to 75/25. **^{1}H NMR** (300 MHz, $CDCl_3$) δ 8.09 (d, *J* = 8.8 Hz, 1H), 7.96 (s, 1H), 7.79 (d, *J* = 8.8 Hz, 1H), 7.62 (d, *J* = 9.0 Hz, 1H), 7.34-7.31 (m, 1H), 7.21 (d, *J* = 9.0 Hz, 1H), 4.50-4.45 (m, 1H), 4.34 (m, 1H), 4.18-4.09 (m, 2H), 4.03 (s, 3H), 3.91 (dd, *J* = 11.5 Hz, 3.9 Hz, 1H), 1.15-1.08 (m, 63H). **^{13}C NMR** (75 MHz, $CDCl_3$) δ 157.6, 138.7, 135.7, 132.3, 131.4, 130.6 (2C), 130.5, 129.4, 118.0, 113.5, 91.1, 87.5, 82.1, 79.0, 75.3, 70.8, 62.2, 57.3, 18.6-18.2 (18C), 13.1 (3C), 12.7 (3C), 12.3 (3C). **HRMS (ESI)** *m/z*: $[M + H]^+$ Calcd for $C_{46}H_{78}IO_5Si_3$ 921.4202; Found 921.4234.

4-(((2*R*,3*R*,4*S*)-5-iodo-3,4-bis((triisopropylsilyl)oxy)-2-(((triisopropylsilyl)oxy) methyl)-3,4-dihydro-2*H*-pyran-6-yl)ethynyl)benzonitrila (3g)

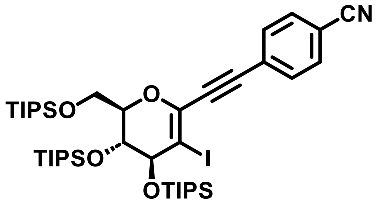

The product was obtained as a pale yellow oil (109.1 mg, 63 %). Eluente: hexane/DCM = 95/5. **^{1}H NMR** (300 MHz, $CDCl_3$) δ 7.62 (m, 4H), 4.47-4.45 (m, 1H), 4.32-4.31 (m, 1H), 4.16 (m, 1H), 4.11 (dd, *J* = 11.5 Hz, 8.1 Hz, 1H), 3.87 (dd, *J* = 11.5 Hz, 3.7 Hz, 1H), 1.14-1.06 (m, 63H). **^{13}C NMR** (75 MHz, $CDCl_3$) δ138.2, 132.4 (2C), 132.1 (2C), 127.1, 118.5, 112.4, 90.9, 89.0, 82.3, 80.5, 75.1, 70.8, 62.1, 18.6-18.1 (18C), 13.1 (3C), 12.7 (3C), 12.2 (3C). **HRMS (ESI)** *m/z*: $[M + Na]^+$ Calcd for $C_{42}H_{72}INO_4Si_3Na$ 888.3711; Found 888.3687.

(((2*R*,3*R*,4*S*)-5-iodo-6-(thiophen-2-ylethynyl)-2-(((triisopropylsilyl)oxy)methyl)-3,4-dihydro-2*H*-pyran-3,4-diyl)bis(oxy))bis(triisopropylsilane) (3h)

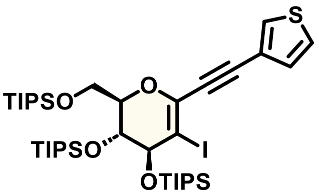

The product was obtained as a yellow oil (89.9 mg, 53 %). Eluente: hexane/DCM = 95/5 to 85/15. **^{1}H NMR** (300 MHz, $CDCl_3$) δ 7.57 (dd, *J* = 3.0 Hz, 0.8 Hz, 1H), 7.28-7.26 (m, 1H), 7.20 (dd, *J* = 5.0 Hz, 0.8 Hz, 1H), 4.48-4.43 (m, 1H), 4.33-4.31 (m, 1H), 4.10 (dd, *J* = 11.4 Hz, 7.8 Hz, 1H), 3.90 (dd, *J* = 11.4 Hz, 4.0 Hz, 1H), 1.14-1.06 (m, 63H). **^{13}C NMR** (75 MHz, $CDCl_3$) δ 138.7, 130.1, 125.4, 121.3, 86.54, 86.51, 82.1, 78.5, 75.2, 70.8, 62.2, 18.6-18.1 (18C), 13.1 (3C), 12.7 (3C), 12.2 (3C). **HRMS (ESI)** *m/z*: $[M + Na]^+$ Calcd for $C_{39}H_{71}IO_4SSi_3Na$ 869.3323; Found 869.3327.

(((2*R*,3*R*,4*S*)-5-iodo-6-(pent-1-yn-1-yl)-2-(((triisopropylsilyl)oxy)methyl)-3,4-dihydro-2*H*-pyran-3,4-diyl)bis(oxy))bis(triisopropylsilane) (3i)

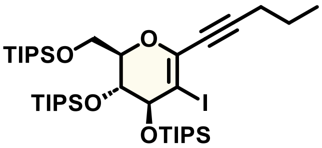

The product was obtained as a pale yellow oil (80.7 mg, 50 %). Eluent: hexane/DCM = 100/0 to 95/5. **^{1}H NMR** (300 MHz, $CDCl_3$) δ 4.41 – 4.38 (m, 1H), 4.28 – 4.26 (m, 1H), 4.13-4.11 (m, 1H), 4.08 – 4.02 (dd, *J* = 11.4 Hz, 7.9 Hz, 1H), 3.86 (dd, *J* = 11.4, 4.1 Hz, 1H), 2.36 (t, *J* = 7.0 Hz, 2H), 1.62 (sext, *J* = 7.2 Hz, 2H), 1.30 – 1.18 (m, 3H), 1.13 – 1.05 (m, 63H). **^{13}C NMR** (75 MHz, $CDCl_3$) δ 138.8, 93.2, 81.9, 78.6, 76.8, 75.3, 70.8, 62.2, 21.8, 21.4, 18.6-18.1 (18C), 13.7, 13.1 (3C), 12.7 (3C), 12.2 (3C). **HRMS (ESI)** *m/z*: $[M + H]^+$ Calcd for $C_{38}H_{76}IO_4Si_3$ 807.4096; Found 807.4110.

2.3 Optimization studies

We commenced our study by employing 2-iodo-1-alkynylglucal and aniline as our coupling partners, in the first experiment, $PdCl_2$ was chosen as palladium source, DIPEA as base, $MoCO_6$ as 'CO' source, 1,4 dioxane was chosen as solvent, as it was suitable to the solubilization of the starting material, the reaction was heated up to 80°C in oil bath over 16 hours. This condition leads to an isolated yield of 26 %. Then we explored other palladium sources, we applied $Pd(OAc)_2$ as catalyst which lead to a slightly increased isolated yield of 31%, the use of a Pd (0) as $Pd(dba)_2$ provided traces of product, as shown in Table 1 entries 1-3. Next, we evaluated the phosphine ligand, PPh_3 did not lead to significant changes in the isolated yield, when compared to the reaction performed with no phosphine (entry 4). Interestingly, when Xantphos was used a slightly increase in isolated yield was obtained, 32% (entry 5) however when DepPhos was applied the product was isolated in 44% yield, entry 6. With this set of conditions, we decided to test different bases, when a organic base such as TEA was applied only 16% of isolated

product was achieved, entry 7. We also testes K_2CO_3 and only 10 % of isolated yield was obtained, entry 8. As the abovementioned set of conditions did not see to efficiently improved the isolated yield, we increased the equivalents of aniline to 2.4 and the isolated yield obtained was 64% entry 9. We continued our study with DIPEA as base and changed the reaction solvent, to toluene, in this experiment the isolated yield decreased to was 55%, entry 10. We then applied acetonitrile as solvent, entry 11 and an improvement in the isolated yield was noticed, the reaction was conducted in 100 °C, and a decrease in the isolated yield was noticed, we decided to apply a reaction time of 32 h, entry 13 and the final isolated yield was 91%. Using other transition metal such as rhodium provided only traces of the desired product. The Carbonylative cyclization was attempted with other metal catalyst, in entry 14 we applied a rhodium catalyst, but only traces of products were detected by analysis of the crude NMR mixture. We also performed the reaction in a two-chamber system and the CO was generated through haloform reaction, in this experiment only 10% of isolated product was achieved, entry 15.

Table 1. Optimization of the reactions conditions[a]

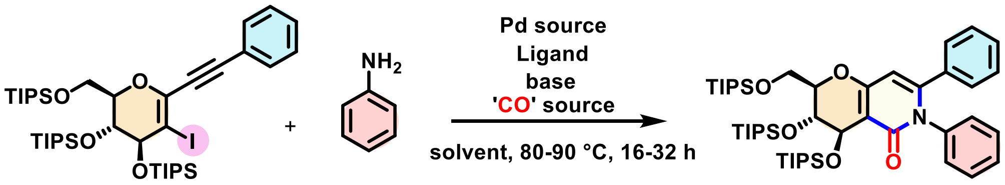

Entry	Catalyst (mol%)	Ligante (10 mol%)	Base	Aniline (equiv.)	Solvent	Time (h)	Yield (%)[b]
				Palladium source			
1	$PdCl_2$	-	DIPEA	1.2	1,4-Dioxane	16	26
2	$Pd(OAc)_2$	-	DIPEA	1.2	1,4-Dioxane	16	31
3	$Pd(dba)_2$	-	DIPEA	1.2	1,4-Dioxane	16	traces
				Ligand			
4	$Pd(OAc)_2$	PPh_3	DIPEA	1.2	1,4-Dioxane	16	27
5	$Pd(OAc)_2$	Xantphos	DIPEA	1.2	1,4-Dioxane	16	32
6	$Pd(OAc)_2$	DEPphos	DIPEA	1.2	1,4-Dioxane	16	44
				Base			
7	$Pd(OAc)_2$	DEPphos	TEA	1.2	1,4-Dioxane	16	16
8	$Pd(OAc)_2$	DEPphos	K_2CO_3	1.2	1,4-Dioxane	16	10
				Aniline			
9	$Pd(OAc)_2$	DEPphos	DIPEA	2.4	1,4-Dioxane	16	60
				Solvent			

10	$Pd(OAc)_2$	DEPphos	DIPEA	2.4	Toluene	16	55
11	$Pd(OAc)_2$	DEPphos	DIPEA	2.4	ACN	16	70
Temperature							
12	$Pd(OAc)_2$	DEPphos	DIPEA	2.4	ACN	16	50[c]
Time							
13	$Pd(OAc)_2$	DEPphos	DIPEA	2.4	ACN	32	91
Other transition metal							
14	$[RhCp^*Cl_2]_2$	DEPphos [d]	DIPEA	2.4	ACN	32	Trace
Other CO source[e]							
15	$Pd(OAc)_2$	DEPphos	DIPEA	2.4	ACN	32	10

[a]Reaction conditions; [b] Isolated yield; [c]Reaction at 100 °C; [d]20 mol %, [e] The reaction was conducted using the system of $CHCl_3$(5 equiv), KOH (10 equiv) in toluene as "CO"source in a two-pot process.

. **2.4 General procedure for Carbonylative cyclization reaction (4 and 5)**

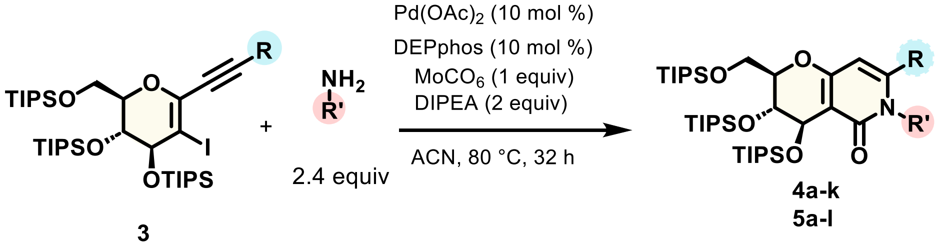

Compound **3** (50 μmol, 1.0 equiv), DIPEA (17.4 μL, 100 μmol, 2.0 equiv), correspondent aniline (120 μmol, 2.4 equiv), $MoCO_6$ (13.2 mg, 50 μmol, 1.0 equiv), DEPphos (2.7 mg, 5 μmol, 0.1 equiv), $Pd(OAc)_2$ (1.1 mg, 5 μmol, 0.1 equiv) and anhydrous acetonitrile (0.5 mL) were added to an oven-dried reaction tube. The reaction mixture was heated to 80 °C in oil bath and stirred for 32 h. Then the mixture was concentrated in vacuo and the residue was purified by flash column chromatography on silica gel to afford the desired products**.**

For reaction with 1 mmol of **3a** was employed: **3a** (841 mg, 1 mmol, 1.0 equiv), DIPEA (0.35 mL, 2 mmol, 2.0 equiv), aniline (0.22 mL, 2.4 mmol, 2.4 equiv), $MoCO_6$ (264 mg, 1 mmol, 1.0 equiv), DEPphos (54 mg, 0,1 mmol, 0.1 equiv), $Pd(OAc)_2$ (22 mg, 0.1 mmol, 0.1 equiv) and anhydrous acetonitrile (5 mL).

(2*R*,3*R*,4*R*)-6,7-diphenyl-3,4-bis((triisopropylsilyl)oxy)-2-(((triisopropylsilyl)oxy)methyl)-2,3,4,6-tetrahydro-5*H*-pyrano[3,2-c]pyridin-5-one (4a).

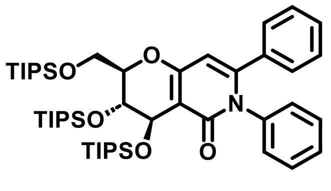

The product was obtained as pale yellow oil (from 0.1 mmol 38 mg, 91 %; from 1 mmol 617 mg, 74%). Eluent: hexane/DCM = 75/25 to 60/40. **[α]D^{20}** = + 6.5 (c = 0.1 in $CHCl_3$). **^{1}H NMR** (300 MHz, $CDCl_3$) δ 7.23 – 7.12 (m, 6 H), 7.08 – 7.05 (m. 3 H), 6.90 (m, 1 H), 5.98 (s, 1 H), 4.90 – 4.89 (m, 1 H), 4.55 – 4.51 (m, 1 H), 4.33 – 4.32 (m, 1 H), 4.20 (dd, *J* = 11.5 Hz, 8.1 Hz 1 H), 4.00 (dd, *J* = 11.6 Hz, 3.3 Hz, 1 H), 1.08 – 1.02 (m, 63 H). **^{13}C NMR** (75 MHz, $CDCl_3$) δ 164.0, 160.5, 148.8, 139.2, 136.0, 129.1 (2C), 128.6, 128.3, 127.9 (2C), 127.6, 106.6, 102.5, 84.0, 69.2, 64.2, 63.7, 18.7 – 18.2 (18C), 12.7 (3C), 12.7 (3C), 12.3 (3C). **IR** (ν, cm^{-1}) = 2844; 2795; 1601; 1520; 1431; 1220; 1048; 1026; 855; 739; 659. **HRMS (ESI)** *m/z*: $[M + Na]^+$ Calcd for $C_{48}H_{79}NO_5Si_3Na$ 856.5164; Found 856.5132.

(2*R*,3*R*,4*R*)-6-(4-methoxyphenyl)-7-phenyl-3,4-bis((triisopropylsilyl)oxy)-2-(((triisopropylsilyl)oxy)methyl)-2,3,4,6-tetrahydro-5*H*-pyrano[3,2-c]pyridin-5-one (4b)

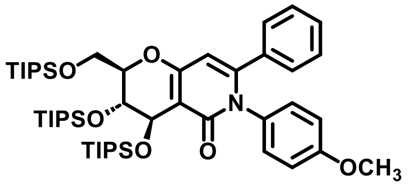

The product was obtained as a pale yellow oil (36.2 mg, 84 %). Eluent: hexane/DCM = 75/25 to 50/50. **[α]D^{20}** = +8.5 (c = 0.1 in $CHCl_3$). **^{1}H NMR** (300 MHz, $(CD_3)_2CO$) δ 7.23 – 7.16 (m, 5 H), 6.94 (t, *J* = 7.4 Hz, 2 H), 6.78 (t, *J* = 8.7 Hz, 2 H), 5.93 (s, 1 H), 4.93 – 4.92 (m, 1 H), 4.61 – 4.58 (m, 1 H), 4.45 – 4.44 (m, 1 H), 4.28 (dd, *J* = 11.4 Hz, 8.2 Hz, 1 H), 4.11 (dd, *J* = 11.5 Hz, 3.4 Hz, 1 H), 3.73 (s, 3 H), 1.12 – 1.07 (m, 63 H). **^{13}C NMR** (75 MHz, $(CD_3)_2CO$) δ 165.0, 161.1, 160.2, 159.9, 137.1, 133.1, 131.9, 131.5, 130.1 (2C), 129.4, 129.0 (2C), 114.7, 107.1, 102.4, 84.7, 70.3, 65.1, 65.0, 55.9, 19.3 – 18.7 (18C), 13.6 (3C), 13.5 (3C) 13.2 (3C). **IR** (ν, cm^{-1}) = 2844; 2769; 1596; 1520; 1462; 1413; 1207; 1026; 855; 739; 659. **HRMS (ESI)** *m/z*: $[M + H]^+$ Calcd for $C_{49}H_{82}NO_6Si_3$ 864.5450; Found 864.5456.

(2*R*,3*R*,4*R*)-6-mesityl-7-phenyl-3,4-bis((triisopropylsilyl)oxy)-2-(((triisopropylsilyl)oxy)methyl)-2,3,4,6-tetrahydro-5*H*-pyrano[3,2-c]pyridin-5-one (4c)

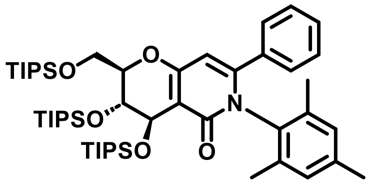

The product was obtained as a caramel solid (35.0 mg, 80 %). Eluent: hexane/DCM = 75/25 to 50/50. mp. 55-57 °C.**[α]D^{20}** = +7.2 (c = 0.1 in $CHCl_3$). **^{1}H NMR** (300 MHz,

$(CD_3)_2CO$) δ 7.27 – 7.19 (m, 5H), 6.79-6.77 (m 2H), 5.99 (s, 1H), 4.92 – 4.91 (m, 1H), 4.64 – 4.61 (m, 1H), 4.45 – 4.44 (m, 1H), 4.35 (dd, *J* = 11.6 Hz, 8.4 Hz, 1H), 4.11 (dd, *J* = 11.6 Hz, 3.1 Hz, 1H), 2.18 (s, 3H), 2.04 (s, 3H), 1.99 (s, 3H) 1.14 – 1.09 (m, 63 H). **^{13}C NMR** (75 MHz, $(CD_3)_2CO$) δ 163.8, 161.1, 150.4, 138.4, 136.7, 136.3, 129.6, 129.5, 129.4, 129.1 (2C), 128.6 (2C), 106.8, 102.5, 84.3, 70.0, 65.1, 64.6, 20.9, 19.1 (2C), 18.9 – 18.3 (18C), 13.4 (3C), 13.3 (3C), 12.9 (3C). **IR** (ν, cm^{-1}) = 2844; 2769; 1596; 1518; 1413; 1373; 1028; 855; 730; 659. **HRMS (ESI)** *m/z*: $[M + H]^+$ Calcd for $C_{51}H_{86}NO_5Si_3$ 876.5814; Found 876.5804.

(2*R*,3*R*,4*R*)-6-(naphthalen-2-yl)-7-phenyl-3,4-bis((triisopropylsilyl)oxy)-2-(((triisopropylsilyl)oxy)methyl)-2,3,4,6-tetrahydro-5*H*-pyrano[3,2-c]pyridin-5-one (4d)

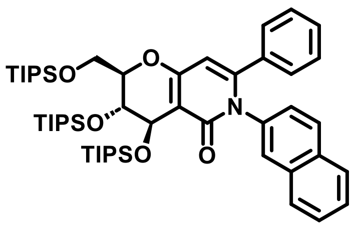

The product was obtained as a pale yellow oil (36.2 mg, 82 %). Eluent: hexane/DCM = 75/25 to 50/50. **$[\alpha]_D^{20}$** = +4.5 (c = 0.1 in $CHCl_3$). **^{1}H NMR** (300 MHz, $(CD_3)_2CO$) δ 7.85-7.74 (m, 3H), 7.53 (s, 1H), 7.47-7.41 (m, 2H), 7.26-7.21 (m, 3H), 7.13-7.10 (m, 3H), 6.00 (s, 1H), 4.94 (m, 1 H), 4.63 - 4.61 (m, 1H), 4.46 (m, 1H), 4.30 (dd, *J* = 11.4 Hz, 8.2 Hz, 1 H), 4.13 (dd, *J* = 11.4 Hz, 3.0 Hz, 1H), 1.11 – 1.07 (m, 63 H). **^{13}C NMR** (75 MHz, $(CD_3)_2CO$) δ 164.7, 161.0, 150.3, 137.9, 136.7, 134.0, 132.2, 129.8, 129.2, 128.8, 128.78, 128.71, 127.3, 127.1, 107.0, 102.5, 84.5, 70.0, 64.8, 64.7, 19.0 – 18.4 (18C), 13.4 (3C), 13.3 (3C) 12.9 (3C). **IR** (ν, cm^{-1}) = 2844; 2769; 1600; 1518; 1413; 1376; 1026; 853; 719; 657. **HRMS (ESI)** *m/z*: $[M + H]^+$ Calcd for $C_{52}H_{82}NO_5Si_3$ 884.5501; Found 884.5496.

(2*R*,3*R*,4*R*)-6-(4-fluorophenyl)-7-phenyl-3,4-bis((triisopropylsilyl)oxy)-2-(((triisopropylsilyl)oxy)methyl)-2,3,4,6-tetrahydro-5*H*-pyrano[3,2-c]pyridin-5-one (4e)

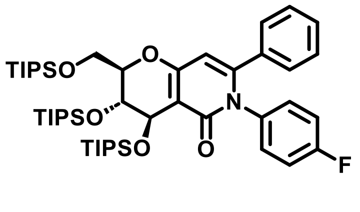

The product was obtained as a pale yellow oil (31.2 mg, 73%). Eluent: hexane/DCM = 75/25 to 50/50. **$[\alpha]_D^{20}$** = +5.2 (c = 0.1 in $CHCl_3$). **^{1}H NMR** (300 MHz, $(CD_3)_2CO$) δ 7.24 – 7.15 (m, 5H), 7.09 – 7.03 (m, 4H), 5.97 (s, 1H), 4.92 (dd, *J* = 3.1 Hz, 1.9 Hz, 1H), 4.63 – 4.58 (m, 1 H), 4.45 (dd, *J* = 3.1 Hz, 1.5 Hz, 1H), 4.28 (dd, *J* = 11.5 Hz, 8.2 Hz, 1H), 4.11 (dd, *J* = 11.5 Hz, 3.4 Hz, 1 H), 1.15 – 1.06 (m, 63 H). **^{13}C NMR** (75 MHz, $(CD_3)_2CO$) δ 164.3, 163.0 (d, *J* = 260 Hz, C-F), 161.2, 150.4, 136.7,

136.6 (d, J = 3.0 Hz, C-F), 132.9 (d, J = 11.2 Hz, C-F), 130.1 (2C), 129.6, 129.1 (2C), 116.2 (d, J= 22.5 Hz, C-F), 107.1, 102.7, 84.7, 70.2, 65.0, 64.9, 19.2 – 18.7 (18C), 13.6 (3C), 13.5 (3C) 13.1 (3C). **IR** (ν, cm^{-1}) = 2844; 2769; 1601; 1520; 1460; 1413; 1376; 1050; 1026; 855; 726; 659. **HRMS (ESI)** *m/z*: $[M + Na]^+$ Calcd for $C_{48}H_{78}FNO_5Si_3Na$ 874.5070; Found 874.5039.

(2*R*,3*R*,4*R*)-7-phenyl-6-(pyridin-2-yl)-3,4-bis((triisopropylsilyl)oxy)-2-(((triisopropylsilyl)oxy)methyl)-2,3,4,6-tetrahydro-5*H*-pyrano[3,2-c]pyridin-5-one (4g).

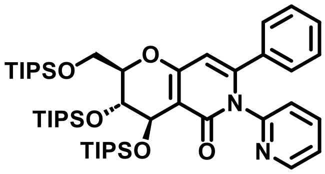

The product was obtained as a pale yellow oil (13.3 mg, 32 %). Eluent: hexane/EtOAc = 90/10 to 65/35. $[\alpha]_D^{20}$ = +4.4 (c = 0.1 in $CHCl_3$). **^{1}H NMR** (300 MHz, $(CD_3)_2CO$) δ 8.26 (ddd, *J* = 4.9 Hz, 1.9 Hz, 0.9 Hz, 1H), 7.77 (td, *J* = 7.8 Hz, 1.9 Hz, 1H), 7.30 (dt, *J* = 7.8 Hz, 0.9 Hz, 1H), 7.21-7.14 (m, 6H), 5.95 (s, 1 H), 4.90 (dd, *J* = 2.8 Hz, 1.9 Hz, 1H), 4.62 – 4.58 (m, 1 H), 4.42 (dd, *J* = 2.9 Hz, 1.4 Hz, 1H), 4.27 (dd, *J* = 11.5 Hz, 8.2 Hz, 1H), 4.10 (dd, *J* = 11.5 Hz, 3.4 Hz, 1H), 1.10 – 1.06 (m, 63 H). **^{13}C NMR** (75 MHz, $(CD_3)_2CO$) δ 164.5, 161.1, 153.2, 149.7, 149.4, 138.1, 136.3, 129.4 (2C), 129.1, 128.6 (2C), 126.0, 123.8, 106.7, 102.4, 84.4, 69.9, 64.59, 64.55, 18.9 – 18.3 (18C), 13.2 (3C), 13.1 (3C), 12.8 (3C). **IR** (ν, cm^{-1}) = 2844; 2769; 1601; 1518; 1417; 1376; 1028; 855; 728; 659. **HRMS (ESI)** *m/z*: $[M + Na]^+$ Calcd for $C_{47}H_{78}N_2O_5Si_3Na$ 857.5116; Found 857.5122.

(2*R*,3*R*,4*R*)-6-ethyl-7-phenyl-3,4-bis((triisopropylsilyl)oxy)-2-(((triisopropylsilyl)oxy)methyl)-2,3,4,6-tetrahydro-5*H*-pyrano[3,2-c]pyridin-5-one (4h)

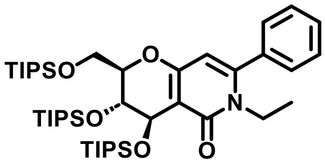

The product was obtained as a yellow oil (28.3 mg, 72 %). Eluent: hexane/DCM = 80/20 to 70/30. $[\alpha]_D^{20}$ = +3.0 (c = 0.1 in $CHCl_3$). **^{1}H NMR** (300 MHz, $CDCl_3$) δ 7.44 – 7.42 (m, 3H), 7.33-7.31 (m, 2H), 5.76 (s, 1 H), 4.89-4.88 (m, 1H), 4.47 – 4.44 (m, 1H), 4.28 – 4.26 (m, 1H), 4.11 (dd, *J* = 11.5 Hz, 8.4 Hz, 1 H), 3.94 (m, 2H), 3.70 (q, *J* = 7.7 Hz, 1H), 1.33 (t, *J* = 7.7 Hz, 3H), 1.09 – 1.03 (m, 63 H). **^{13}C NMR** (75 MHz, $CDCl_3$) δ 163.5, 159.6, 148.9, 136.1, 129.0, 128.6 (2C), 128.5 (2C), 106.5, 102.1, 83.6, 69.2, 64.1, 63.8, 40.3, 19.6 – 17.8 (18C), 14.4, 12.8 (3C), 12.6 (3C), 12.2 (3C). **IR**

(ν, cm^{-1}) = 2827; 2771; 1594; 1525; 1411; 1220; 1028; 983; 855; 732; 661. **HRMS (ESI)** *m/z*: $[M + Na]^+$ Calcd for $C_{44}H_{79}NO_5Si_3Na$ 808.5164; Found 808.5133.

(2*R*,3*R*,4*R*)-6-isopropyl-7-phenyl-3,4-bis((triisopropylsilyl)oxy)-2-(((triisopropylsilyl)oxy)methyl)-2,3,4,6-tetrahydro-5*H*-pyrano[3,2-c]pyridin-5-one (4i)

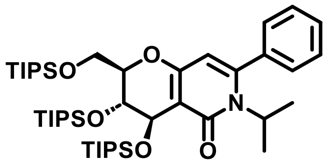

The product was obtained as a yellow oil (19.1 mg, 48 %). Eluent: hexane/DCM = 85/15 to 65/35. **$[\alpha]_D^{20}$** = +5.7 (c = 0.1 in $CHCl_3$). **^{1}H NMR** (300 MHz, $(CD_3)_2CO$) δ 7.52 – 7.50 (m, 3H), 7.40 – 7.36 (m, 2H), 5.65 (s, 1H), 4.92 (dd, *J* = 2.9 Hz, 1.9 Hz, 1H), 4.52 – 4.48 (m, 1H), 4.40 (dd, *J* = 3.0 Hz, 1.4 Hz, 1H), 4.20 (dd, *J* = 11.5 Hz, 8.2 Hz, 1H), 4.14-4.09 (m, 1H), 4.05 (dd, *J* = 11.5 Hz, 3.4 Hz, 1H), 1.51 (d, *J* = 6.7 Hz, 3H), 1.45 (d, *J* = 6.7 Hz, 3H), 1.14 – 1.06 (m, 63 H). **^{13}C NMR** (75 MHz, $(CD_3)_2CO$) δ 164.8, 159.7, 150.7, 137.7, 129.9, 129.6 (2C), 128.8 (2C), 107.9, 102.2, 84.1, 70.0, 64.7, 64.6, 53.5, 19.9, 19.7, 19.0 – 18.4 (18C), 13.4 (3C), 13.3 (3C) 12.8 (3C). **IR** (ν, cm^{-1}) = 2844; 2769; 1592; 1527; 1413; 1220; 1127; 1026; 855; 730; 657. **HRMS (ESI)** *m/z*: $[M + H]^+$ Calcd for $C_{45}H_{82}NO_5Si_3$ 800.5501; Found 800.5494.

(2*R*,3*R*,4*R*)-6-benzyl-7-phenyl-3,4-bis((triisopropylsilyl)oxy)-2-(((triisopropylsilyl)oxy)methyl)-2,3,4,6-tetrahydro-5*H*-pyrano[3,2-c]pyridin-5-one (4j)

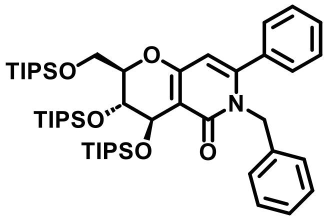

The product was obtained as a pale yellow oil (23.7 mg, 56 %). Eluent: hexane/EtOAc = 75/25 to 60/40. **$[\alpha]_D^{20}$** = +3.6 (c = 0.1 in $CHCl_3$). **^{1}H NMR** (300 MHz, $(CD_3)_2CO$) δ 7.45-7.33 (m, 3H), 7.18-7.15 (m, 5H), 6.88-6.85 (m, 2 H), 5.80 (s, 1 H), 5.16 (d, *J* = 15.8 Hz, 1H), 5.10 (d, *J* = 15.8 Hz, 1H), 4.96 – 4.94 (m, 1 H), 4.59 – 4.55 (m, 1 H), 4.44 – 4.42 (m, 1 H), 4.27 (dd, *J* = 11.5 Hz, 8.3 Hz, 1H), 4.08 (dd, *J* = 11.5 Hz, 3.3 Hz, 1H), 1.13 – 1.06 (m, 63 H). **^{13}C NMR** (75 MHz, $(CD_3)_2CO$) δ 164.6, 160.5, 150.8, 139.1, 136.3, 130.0, 129.4 (2C), 129.1 (2C), 128.9 (2C), 127.5, 127.4 (2C), 106.7, 102.5, 84.2, 70.0, 65.1, 64.5, 48.3, 19.0 – 18.4 (18C), 13.4 (3C), 13.3 (3C), 12.9 (3C). **IR** (ν, cm^{-1}) = 2844; 2769; 1594; 1523; 1411; 1376; 1028; 855; 732; 659. **HRMS (ESI)** *m/z*: $[M + H]^+$ Calcd for $C_{49}H_{82}NO_5Si_3$ 848.5501; Found 848.5515.

(2*R*,3*R*,4*R*)-7-phenyl-6-(pyridin-2-ylmethyl)-3,4-bis((triisopropylsilyl)oxy)-2-(((triisopropylsilyl)oxy)methyl)-2,3,4,6-tetrahydro-5*H*-pyrano[3,2-c]pyridin-5-one (4k)

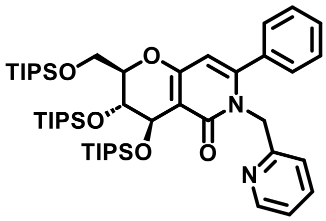

The product was obtained as a yellow oil (23.3 mg, 55 %). Eluent: hexane/EtOAc = 95/5 to 90/10. **[α]$_D$^{20}** = +4.2 (c = 0.1 in $CHCl_3$). **^{1}H NMR** (300 MHz, $(CD_3)_2CO$) δ 8.39 (d, *J* = 4.7 Hz, 1H), 7.63 (td, *J* = 7.7 Hz, 1.7 Hz, 1H), 7.40-7.33 (m, 5H), 7.16 (dd, *J* = 7.5 Hz, 4.7 Hz, 1H), 7.01 (d, *J* = 7.7 Hz, 1H), 5.84 (s, 1 H), 5.11 (s, 2H), 4.90 (m, 1H), 4.59 - 4.55 (m, 1 H), 4.41 (dd, *J* = 2.8 Hz, 1.3 Hz, 1H), 4.26 (dd, *J* = 11.5 Hz, 8.3 Hz, 1H), 4.08 (dd, *J* = 11.5 Hz, 3.3 Hz, 1H), 1.10 – 1.03 (m, 63 H). **^{13}C NMR** (75 MHz, $(CD_3)_2CO$) δ 164.3, 160.6, 158.3, 151.0, 149.7, 136.9, 136.4, 130.0, 129.3 (2C), 129.2 (2C), 122.5, 121.6, 106.6, 102.2, 84.2, 70.0, 65.0, 64.5, 50.7, 19.0 – 18.4 (18C), 13.3 (3C), 13.2 (3C), 12.8 (3C). **IR** (ν, cm^{-1}) = 2844; 2769; 1594; 1523; 1413; 1376; 1028; 855; 730; 659. **HRMS (ESI)** *m/z*: $[M + H]^+$ Calcd for $C_{48}H_{81}N_2O_5Si_3$ 849.5453; Found 849.5424.

(2*R*,3*R*,4*R*)-7-(4-ethoxyphenyl)-6-phenyl-3,4-bis((triisopropylsilyl)oxy)-2-(((triisopropylsilyl)oxy)methyl)-2,3,4,6-tetrahydro-5*H*-pyrano[3,2-c]pyridin-5-one (5a)

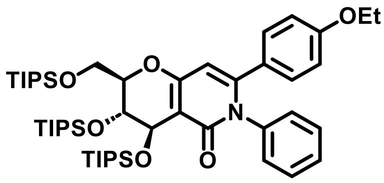

The product was obtained as a yellow oil (22 mg, 50 %). Eluent: hexane/DCM = 70/30 to 60/40. **[α]$_D$^{20}** = +4.1 (c = 0.1 in $CHCl_3$). **^{1}H NMR** (300 MHz, $(CD_3)_2CO$) δ 7.28 – 7.16 (m, 3H), 7.07 – 7.04 (m. 4H), 6.72 (d, *J* = 8.7 Hz, 2 H), 5.92 (s, 1 H), 4.93 – 4.91 (m, 1 H), 4.61 – 4.56 (m, 1 H), 4.45 (dd, *J* = 2.9 Hz, 1.3 Hz, 1H), 4.28 (dd, *J* = 11.5 Hz, 8.2 Hz, 1H), 4.11 (dd, *J* = 11.5 Hz, 3.4 Hz, 1H), 3.96 (d, *J* = 6.9 Hz, 2 H), 1.33 – 1.28 (m, 3 H), 1.12 – 1.06 (m, 63H). **^{13}C NMR** (75 MHz, $(CD_3)_2CO$) δ 164.6, 160.9, 159.9, 150.1, 140.5, 131.2 (2C), 130.7, 130.5, 129.1 (2C), 128.8, 128.2, 114.6 (2), 106.5, 102.1, 84.4, 70.0, 64.8, 64.7, 64.0, 19.0 – 18.4 (18C), 14.9, 13.37 (3C), 13.32 (3C), 12.9 (3C). **IR** (ν, cm^{-1}) = 2844; 2769; 1596; 1520; 1462; 1208; 1026; 855; 732; 659. **HRMS (ESI)** *m/z*: $[M + K]^+$ Calcd for $C_{50}H_{83}NO_6Si_3K$ 916.5165; Found 916.5165.

(2*R*,3*R*,4*R*)-7-(4-fluorophenyl)-6-phenyl-3,4-bis((triisopropylsilyl)oxy)-2-(((triisopropylsilyl)oxy)methyl)-2,3,4,6-tetrahydro-5*H*-pyrano[3,2-c]pyridin-5-one (5b)

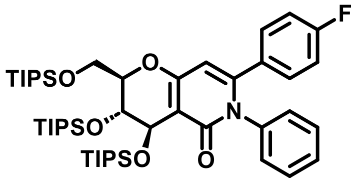

The product was obtained as a white solid (24.6 mg, 58 %). Eluent: hexane/DCM = 75/25 to 60/40. mp. 96-99 °C. **[α]$_D^{20}$** = +6.3 (c = 0.1 in $CHCl_3$). **^{1}H NMR** (300 MHz, $(CD_3)_2CO$) δ δ 7.29 – 7.18 (m, 5H), 7.07 – 7.02 (m, 2H), 6.98 – 6.93 (m, 2H), 5.97 (s, 1H), 4.92 (dd, *J* = 3.0 Hz, 1.9 Hz, 1H), 4.62 – 4.58 (m, 1H), 4.45 (dd, *J* = 3.0 Hz, 1.5 Hz, 1H), 4.28 (dd, *J* = 11.5 Hz, 8.2 Hz, 1H), 4.11 (dd, *J* = 11.5 Hz, 3.4 Hz, 1H), 1.12 – 1.06 (m, 63 H). **^{13}C NMR** (75 MHz, $(CD_3)_2CO$) δ 164.4, 163.2 (d, *J* = 245 Hz, C-F), 160.9, 149.0, 140.2, 133.0 (d, *J* = 3.0 Hz, C-F), 132.1 (d, *J* = 8.25 Hz, C-F), 130.7, 130.4, 129.3 (2C), 128.4, 115.7 (d, *J* = 21.7 Hz, C-F), 107.1, 102.5, 84.5, 70.0, 64.8, 64.7, 19.0 – 18.4 (18C), 13.37 (3C), 13.31 (3C), 12.9 (3C). **IR** (ν, cm^{-1}) = 2844; 2769; 1600; 1526; 1413; 1182; 1141; 1058; 1026; 981; 855; 728; 659. **HRMS (ESI)** *m/z*: $[M + K]^+$ Calcd for $C_{48}H_{78}FNO_5Si_3K$ 890.4809; Found 890.4819.

4-((2*R*,3*R*,4*R*)-5-oxo-6-phenyl-3,4-bis((triisopropylsilyl)oxy)-2-(((triisopropylsilyl)oxy)methyl)-3,4,5,6-tetrahydro-2*H*-pyrano[3,2-c]pyridin-7-yl)benzonitrile (5c)

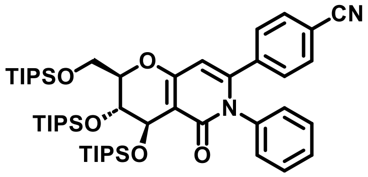

The product was obtained as a bege solid (13.7 mg, 32 %). Eluent: hexane/DCM = 70/30 to 60/40. mp. 135-137 °C **[α]$_D^{20}$** = +8.7 (c = 0.1 in $CHCl_3$). **^{1}H NMR** (300 MHz, $(CD_3)_2CO$) δ 7.62 (dd, *J* = 7.9 Hz, 2H), 7.41 (dd, *J* = 7.9 Hz, 2H), 7.31 – 7.22 (m, 3H), 7.12 – 7.06 (m. 2H), 6.06 (s, 1 H), 4.93 – 4.92 (m, 1 H), 4.62 – 4.60 (m, 1 H), 4.46-4.45 (m, 1H), 4.28 (dd, *J* = 11.7 Hz, 8.7 Hz, 1H), 4.11 (dd, *J* = 11.6 Hz, 3.6 Hz, 1H), 1.14 – 1.06 (m, 63H). **^{13}C NMR** (75 MHz, $(CD_3)_2CO$) δ 164.5, 161.0, 148.5, 141.1, 140.0, 131.8 (2C), 131.1 (2C), 130.9, 130.7, 129.7 (2C), 128.9, 118.9, 113.2, 107.9, 103.2, 84.8, 70.1, 65.0, 64.8, 19.2 – 18.6 (18C), 13.6 (3C), 13.5 (3C), 13.1 (3C). **IR** (ν, cm^{-1}) = 2844; 2769; 1596; 1520; 1361; 1222; 1138; 1076; 1052; 1028; 985; 661. **HRMS (ESI)** *m/z*: $[M + Na]^+$ Calcd for $C_{49}H_{78}N_2O_5Si_3Na$ 881.5116; Found 881.5119.

(2*R*,3*R*,4*R*)-7-(6-methoxynaphthalen-2-yl)-6-phenyl-3,4-bis((triisopropylsilyl)oxy)-2-(((triisopropylsilyl)oxy)methyl)-2,3,4,6-tetrahydro-5*H*-pyrano[3,2-c]pyridin-5-one (5d)

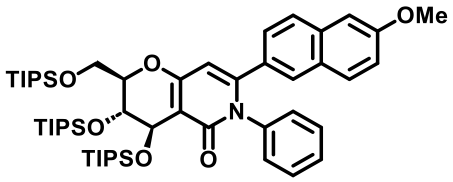

The product was obtained as a pale yellow solid. Eluent: hexane/DCM = 75/25 to 60/40. mp. 182-185 ºC (33.9 mg, 73 %). **[α]$_D$^{20}** = +5.4 (c = 0.1 in $CHCl_3$). **^{1}H NMR** (300 MHz, $(CD_3)_2CO$) δ 7.74-7.71 (m, 2H), 7.53 (d, *J* = 8.5 Hz, 1H), 7.17-7.03 (m, 8H), 6.06 (s, 1 H), 4.95(m, 1 H), 4.62 – 4.60 (m, 1 H), 4.47-4.46 (m, 1H), 4.29 (dd, *J* = 11.4 Hz, 8.4 Hz, 1H), 4.13 (dd, *J* = 11.5 Hz, 2.9 Hz, 1H), 3.87 (s, 3H), 1.12 – 1.08 (m, 63H). **^{13}C NMR** (75 MHz, $(CD_3)_2CO$) δ 164.6, 161.0, 159.5, 150.3, 140.4, 135.1, 131.8, 130.8, 130.5, 129.2 (2C), 129.1 (2C), 129.0, 128.2, 127.5, 127.0, 120.1, 106.8, 106.5, 102.6, 84.4, 70.0, 64.8, 64.7, 55.7, 19.0 – 18.4 (18C), 13.38 (3C), 13.32 (3C), 12.9 (3C). **IR** (ν, cm^{-1}) = 2844; 2771; 1601; 1520; 1411; 1130; 1026; 855; 730; 657. **HRMS (ESI)** *m/z*: $[M + K]^+$ Calcd for $C_{53}H_{83}NO_6Si_3K$ 952,5165; Found 952.5157.

(2*R*,3*R*,4*R*)-7-([1,1'-biphenyl]-4-yl)-6-phenyl-3,4-bis((triisopropylsilyl)oxy)-2-(((triisopropylsilyl)oxy)methyl)-2,3,4,6-tetrahydro-5*H*-pyrano[3,2-c]pyridin-5-one (5e)

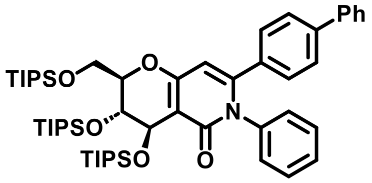

The product was obtained as a pale yellow solid (35.5 mg, 78 %). Eluent: hexane/DCM = 75/25 to 60/40. mp. 260°C dec. **[α]$_D$^{20}** = +3.3 (c = 0.1 in $CHCl_3$). **^{1}H NMR** (300 MHz, $(CD_3)_2CO$) 7.59-7.56 (m, 2H), 7.50 (dd, *J* = 8.4 Hz, 1.9 Hz, 2H), 7.42 (td, *J* = 8.4 Hz, 1.9 Hz, 2H), 7.36-7.28 (m, 2H), 7.27-7.18 (m, 4H), 7.12-7.05 (m, 2H), 6.03 (s, 1H), 4.94 (m, 1H), 4.62– 4.60 (m, 1H), 4.46-4.45 (m, 1H), 4.29 (dd, *J* = 11.5 Hz, 8.1 Hz, 1H), 4.12 (dd, *J* = 11.5 Hz, 3.4 Hz, 1H), 1.12 – 1.06 (m, 63H). **^{13}C NMR** (75 MHz, $(CD_3)_2CO$) δ 164.7, 161.2, 150.0, 141.8, 140.7, 140.5, 135.8, 130.9, 130.6 (2C), 129.9 (2C), 129.5 (2C), 128.8, 128.6, 127.8 (2C), 127.3 (2C), 107.2, 102.7, 84.7, 70.2, 65.0, 64.9, 19.2 – 18.6 (18C), 13.6 (3C), 13.5 (3C) 13.1 (3C). **IR** (ν, cm^{-1}) = 2844; 2769; 1600; 1520; 1411; 1024; 855; 661. **HRMS (ESI)** *m/z*: $[M + Na]^+$ Calcd for $C_{54}H_{83}NO_5Si_3Na$ 932.5477; Found 932.5456.

(2*R*,3*R*,4*R*)-6-phenyl-7-(pyridin-3-yl)-3,4-bis((triisopropylsilyl)oxy)-2-(((triisopropylsilyl)oxy)methyl)-2,3,4,6-tetrahydro-5*H*-pyrano[3,2-c]pyridin-5-one (5f)

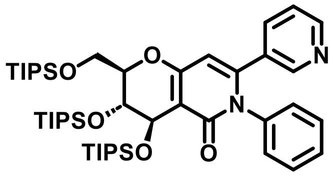

The product was obtained as a caramel oil (28.6 mg, 72 %). Eluent: hexane/EtOAc = 90/10 to 75/25. **[α]$_D$^{20}** = +13.7 (c = 0.1 in $CHCl_3$). **^{1}H NMR** (300 MHz, $(CD_3)_2CO$) δ 8.47-8.46 (m, 2H), 7.62-7.58 (m, 1H), 7.38-7.23 (m, 4H), 7.16 (t, *J* = 7.3 Hz, 1H), 6.12 (s, 1H), 5.01-5.00 (m, 1H), 4.62 – 4.58 (m, 1H), 4.71-4.67 (m, 1H), 4.54-4.52 (m, 1H), 4.35 (dd, *J* = 11.4 Hz, 8.0 Hz, 1H), 4.19 (dd, *J* = 11.5 Hz, 3.5 Hz, 1H), 1.19 – 1.14 (m, 63H). **^{13}C NMR** (75 MHz, $(CD_3)_2CO$) δ 164.4, 160.8, 150.2, 150.1, 146.9, 139.9, 137.0, 132.5, 130.8, 130.5, 129.5 (2C), 128.6, 123.4, 107.5, 103.0, 84.5, 69.9, 64.7, 64.6, 18.9 – 18.4 (18C), 13.36 (3C), 13.31 (3C), 12.9 (3C). **IR** (ν, cm^{-1}) = 2844; 2769; 1601; 1516; 1413; 1028; 855; 726; 659. **HRMS (ESI)** *m/z*: $[M + H]^+$ Calcd for $C_{47}H_{79}N_2O_5Si_3$ 835.5297; Found 835.5285.

(2*R*,3*R*,4*R*)-6-phenyl-7-(thiophen-3-yl)-3,4-bis((triisopropylsilyl)oxy)-2-(((triisopropylsilyl)oxy)methyl)-2,3,4,6-tetrahydro-5*H*-pyrano[3,2-c]pyridin-5-one (5g)

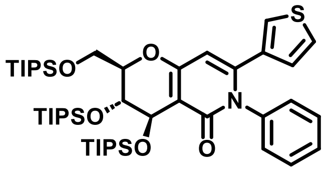

The product was obtained as a pale yellow solid (26.0 mg, 62 %). Eluent: hexane/DCM = 75/25 to 60/40. mp. 57-60 ºC. **[α]$_D$^{20}** = +12.4 (c = 0.1 in $CHCl_3$). **^{1}H NMR** (300 MHz, $(CD_3)_2CO$) δ 7.4-7.28 (m, 4H), 7.25-7.22 (m, 1H), 7.11-7.09 (m, 2H), 6.70 (dd, *J* = 5.0 Hz, 1.2 Hz, 1H), 6.08 (s, 1H), 4.91 (m, 1H), 4.60-4.58 (m, 1H), 4.44-4.43 (m, 1H), 4.26 (dd, *J* = 11.4 Hz, 8.2 Hz, 1H), 3.90 (dd, *J* = 11.4 Hz, 3.4 Hz 1H), 1.12-1.05 (m, 63H). **^{13}C NMR** (75 MHz, $CDCl_3$) δ 164.5, 161.0, 145.2, 140.5, 136.8, 130.5, 130.1, 129.4 (2C), 128.7, 128.6, 126.9, 126.3, 106.9, 102.0, 84.4, 70.0, 64.8, 64.7, 19.0-18.4 (18C), 13.3 (3C), 13.2 (3C), 12.9 (3C). **IR** (ν, cm^{-1}) = 2844; 2769; 1600; 1520; 1413; 1220; 1026; 983; 855; 763; 728; 659. **HRMS (ESI)** *m/z*: $[M + K]^+$ Calcd for $C_{46}H_{77}NO_5SSi_3K$ 878.4467; Found 878.4475.

(2*R*,3*R*,4*R*)-6-phenyl-7-propyl-3,4-bis((triisopropylsilyl)oxy)-2-(((triisopropylsilyl)oxy)methyl)-2,3,4,6-tetrahydro-5*H*-pyrano[3,2-c]pyridin-5-one (5h)

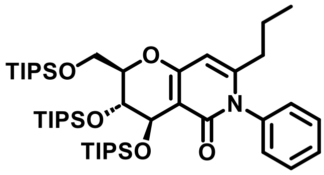

The product was obtained as a pale yellow oil (28.7 mg, 72 %). Eluent: hexane/DCM = 75/25 to 60/40. **[α]D[20]** = +4.8 (c = 0.1 in $CHCl_3$). **^{1}H NMR** (300 MHz, $(CD_3)_2CO$) δ 7.55 – 7.49 (m, 2H), 7.46-7.42 (m, 1H), 7.17 – 7.14 (m, 1H), 5.86 (s, 1 H), 4.84 (dd, *J* = 2.9 Hz, 1.9 Hz, 1H), 4.57 – 4.52 (m, 1H), 4.38 (dd, *J* = 3.0 Hz, 1.6 Hz, 1H), 4.23 (dd, *J* = 11.5 Hz, 8.2 Hz, 1H), 4.07 (dd, *J* = 11.5 Hz, 3.4 Hz, 1H), 2.21 (t, *J* = 7.1 Hz, 2H), 1.42-1.34 (m, 2H), 1.13 – 1.02 (m, 63 H), 0.77 (t, *J* = 7.2 Hz, 3H). **^{13}C NMR** (75 MHz, $(CD_3)_2CO$) δ 165.0, 161.2, 150.4, 140.0, 130.1, 130.0 (2C), 129.8, 129.0, 105.6, 99.6, 84.3, 70.0, 64.8, 64.7, 35.9, 21.8, 18.9 – 18.4 (18C), 13.6, 13.35 (3C), 13.30 (3C), 12.9 (3C). **IR** (ν, cm^{-1}) = 2844; 2769; 1601; 1521; 1413; 1357; 1220; 1026; 855; 728; 659. **HRMS (ESI)** *m/z*: $[M + Na]^+$ Calcd for $C_{45}H_{81}NO_5Si_3Na$ 822.5301; Found 822.5297.

(2*R*,3*R*,4*R*)-7-propyl-6-(4-(trifluoromethyl)phenyl)-3,4-bis((triisopropylsilyl)oxy)-2-(((triisopropylsilyl)oxy)methyl)-2,3,4,6-tetrahydro-5*H*-pyrano[3,2-c]pyridin-5-one (5i)

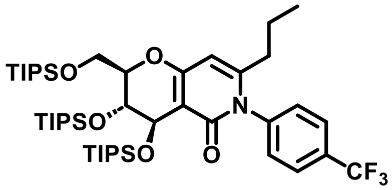

The product was obtained as a pale yellow oil (26.0mg, 60 %). Eluent: hexane/DCM = 75/25 to 60/40. **[α]D[20]** = +5.2 (c = 0.1 in $CHCl_3$). **^{1}H NMR** (300 MHz, $(CDCl_3)$) δ 7.76-7.72 (m, 2H), 7.32-7.20 (m, 2H), 5.85 (s, 1H), 4.84 (dd, *J* = 2.8 Hz, 1.7 Hz, 1H), 4.52 – 4.47 (m, 1H), 4.38 (dd, *J* = 3.2 Hz, 1.4 Hz, 1H), 4.16 (dd, *J* = 11.5 Hz, 8.1 Hz, 1H), 3.96 (dd, *J* = 11.5 Hz, 3.3 Hz, 1H), 2.09 (m, 2H), 1.45-1.37 (m, 2H), 1.09 – 1.00(m, 63 H), 0.80 (t, *J* = 7.2 Hz, 3H). **^{13}C NMR** (75 MHz, $(CDCl_3)$) δ 164.3, 161.1, 148.3, 142.2, 130.7 (q, *J* = 32.5 Hz, C-F), 129.7, 129.6, 126.8 (q, *J* = 3.4 Hz, C-F), 126.6 (q, *J* = 3.4 Hz, C-F), 123.9 (q, *J* = 270 Hz, C-F), 105.5, 100.0, 84.0, 69.0, 64.2, 63.4, 35.3, 20.9, 18.6 – 18.1 (18C), 13.4, 12.6 (6C), 12.2 (3C). **IR** (ν, cm^{-1}) = 2846; 2771; 1601; 1527; 1413; 1281; 1028; 855; 657. **HRMS (ESI)** *m/z*: $[M + Na]^+$ Calcd for $C_{46}H_{80}F_3NO_5Si_3Na$ 890.5194; Found 890.5144.

(2*R*,3*R*,4*R*)-6-ethyl-7-propyl-3,4-bis((triisopropylsilyl)oxy)-2-(((triisopropylsilyl)oxy)methyl)-2,3,4,6-tetrahydro-5*H*-pyrano[3,2-c]pyridin-5-one (5j)

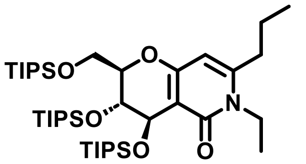

The product was obtained as a pale yellow oil (26.3 mg, 35 %). Hexane/DCM = 90/10 to 75/25. **[α]D[20]** = +14 (c = 0.1 in $CHCl_3$). **^{1}H NMR** (300 MHz, $(CD_3)_2CO$) 5.73 (s, 1H), 4.85 (m, 1H), 4.49 – 4.47 (m, 1H), 4.34 (m, 1H), 4.16 (dd, *J* = 11.5 Hz, 8.3 Hz, 1H), 4.10-3.95 (m, 3H), 2.64 (t, *J* = 7.3 Hz, 2H), 1.66 (sext, *J* = 7.3 Hz, 2H), 1.40-1.35 (m, 3H), 1.19 (t, *J* = 7.3 Hz, 3H), 1.11 – 1.04 (m, 63H). **^{13}C NMR** (75 MHz, $(CD_3)_2CO$) δ 164.4, 160.4, 150.1, 105.3, 99.6, 84.0, 70.1, 64.9, 64.8, 38.6, 35.0, 22.6, 19.0 – 18.4 (18C), 14.5, 13.7, 13.4 (3C), 13.2 (3C) 12.8 (3C). **IR** (ν, cm^{-1}) = 2844; 2771; 1594; 1529; 1413; 1134; 1074; 1048; 1026; 855; 730; 657. **HRMS (ESI)** *m/z*: $[M + Na]^+$ Calcd for $C_{41}H_{81}NO_5Si_3Na$ 774.5320; Found 774.5322.

(2*R*,3*R*,4*R*)-6-ethyl-7-(4-fluorophenyl)-3,4-bis((triisopropylsilyl)oxy)-2-(((triisopropylsilyl)oxy)methyl)-2,3,4,6-tetrahydro-5*H*-pyrano[3,2-c]pyridin-5-one (5k)

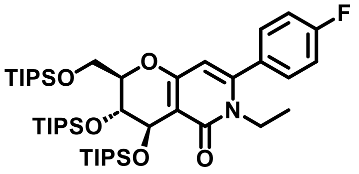

The product was obtained as a yellow oil (28.9 mg, 72 %). Eluent: hexane/DCM = 70/30 to 60/40. **[α]D[20]** = +3.4 (c = 0.1 in $CHCl_3$). **^{1}H NMR** (300 MHz, $(CD_3)_2CO$) δ 7.50 – 7.45 (m, 2H), 7.36 – 7.32 (m, 2H), 5.73 (s, 1H), 4.92 (dd, *J* = 2.8 Hz, 1.9 Hz, 1H), 4.54 – 4.51 (m, 1H), 4.40 (dd, *J* = 2.9 Hz, 1.5 Hz, 1H), 4.20 (dd, *J* = 11.5 Hz, 8.2 Hz, 1H), 4.05 (dd, *J* = 11.5 Hz, 3.4 Hz, 1H), 3.96-3.87 (m, 1H), 3.77-3.68 (m, 1H), 1.39 (t, *J* = 7.6 Hz, 3H), 1.13 – 1.06 (m, 63 H). **^{13}C NMR** (75 MHz, $(CD_3)_2CO$) δ 164.2, 164.1 (d, *J* = 246 Hz, C-F), 160.3, 149.5, 133.2 (d, *J* = 3.2 Hz, C-F), 132.9 (d, *J* = 8.4 Hz, C-F, 2C), 116.7 (d, *J*= 21.8 Hz, C-F, 2C), 107.2, 102.5, 84.4, 70.3, 65.2, 64.8, 40.9, 19.2 – 18.6 (18C), 14.7, 13.7 (3C), 13.5 (3C), 13.1 (3C). **IR** (ν, cm^{-1}) = 2844; 2771; 1594; 1557; 1527; 1460; 1413; 1026; 855; 733; 657. **HRMS (ESI)** *m/z*: $[M + Na]^+$ Calcd for $C_{44}H_{78}FNO_5Si_3Na$ 826,5070; Found 826.5035.

(2*R*,3*R*,4*R*)-6-ethyl-7-(4-methoxyphenyl)-3,4-bis((triisopropylsilyl)oxy)-2-(((triisopropylsilyl)oxy)ethyl)-2,3,4,6-tetrahydro-5*H*-pyrano[3,2-c]pyridin-5-one (5l)

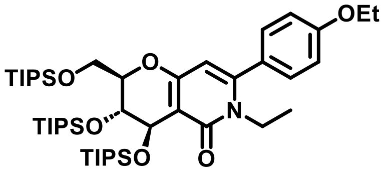

The product was obtained as a yellow oil (19.5 mg, 47 %). Eluent: hexane/DCM = 70/30 to 60/40. **[α]D[20]** = +3.7 (c = 0.1 in $CHCl_3$). **^{1}H NMR** (300 MHz, $CDCl_3$) δ 7.31

(d, J = 8.6 Hz, 2H), 7.03 (d, J = 8.6 Hz, 2H), 5.70 (s, 1 H), 4.91 (m, 1H), 4.53 – 4.50 (m, 1H), 4.39 (m, 1H), 4.20 (dd, J = 11.5 Hz, 8.3 Hz, 1 H), 4.13 (q, J = 6.9 Hz, 2H), 4.05 (dd, J = 11.5 Hz, 3.3 Hz, 1 H), 3.94 (q, J = 7.0 Hz, 1H), 3.75 (q, J = 7.0 Hz, 1H), 1.40 (t, J = 7.0 Hz, 3H), 1.13 – 1.06 (m, 63 H). **^{13}C NMR** (75 MHz, $CDCl_3$) δ 164.1, 160.6, 160.1, 150.3, 130.6 (2C), 128.7, 115.3 (2C), 106.5, 102.1, 84.0, 70.1, 64.9, 64.6, 64.2, 40.6, 19.0 – 18.4 (18C), 15.0, 14.5, 13.4 (3C), 13.3 (3C), 12.9 (3C). **IR** (ν, cm^{-1}) = 2844; 2769; 1592; 1527; 1462; 1207; 1080; 1048; 1026; 855; 730; 659. **HRMS (ESI)** *m/z*: $[M + Na]^+$ Calcd for $C_{46}H_{83}NO_6Si_3Na$ 852.5426; Found 852.5255.

2.5 Procedure for Deprotection (6).[3]

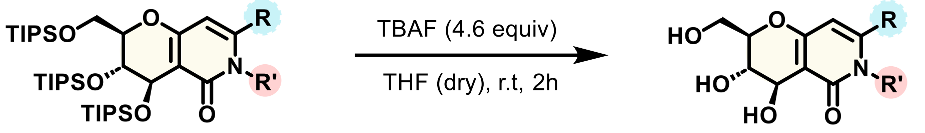

4 or 5 → 6a-b

A solution of TBAF (1 M in THF, 0.324 mmol, 3.6 equiv.) was added to a solution of **4a** or **5i** (0.09 mmol, 1.0 equiv.) in anhydrous THF (500 µL) at room temperature, under N_2 atmosphere. The mixture was stirred at room temperature for 2 hours, and then quenched with water (200 µL). To the crude mixture was added silica gel for column chromatography and the solvent removed under reduced pressure. The product was purified by flash column chromatography using EtOAc/MeOH = 100/0 to 90/10 as eluent.

(2*R*,3*S*,4*R*)-3,4-dihydroxy-2-(hydroxymethyl)-6,7-diphenyl-2,3,4,6-tetrahydro-5*H*-pyrano[3,2-c]pyridin-5-one (6a)

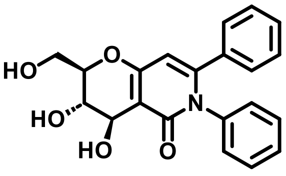

The product was obtained as a white solid (28.0 mg, 85 %) mp. 200-203°C. **$[\alpha]_D^{20}$** = +8.2 (c = 0.1 in MeOH). **^{1}H NMR** (300 MHz, DMSO-*d6*) δ 7.26-7.10 (m, 10H), 5.96 (s, 1 H), 5.34-5.33 (m, 2H), 4.85 (t, J = 5.5 Hz, 1H), 4.47-4.45 (m, 1H), 4.23 (dd, J = 9.5 Hz, 4.6 Hz, 1 H), 3.87-3.72 (m, 3H). **^{13}C NMR** (75 MHz, $(CD_3)_2CO$) δ 163.2, 160.0, 148.4, 138.4, 135.4, 129.5 (2C), 128.8 (2C), 128.3, 128.2 (2C), 127.7 (2C), 127.5, 106.2, 101.2, 81.1, 67.1, 64.0, 60.2. **IR** (ν, cm^{-1}) = 3287; 2833; 2790; 1579; 1510; 1443; 1406; 1368; 1339; 1168; 1130; 981; 743; 678. **HRMS (ESI)** *m/z*: $[M + K]^+$ Calcd for $C_{21}H_{19}NO_5K$ 404.0900.; Found 404.0885.

[3] Yi, D.; Zhu, F.; Walczak, M. A. *Org. Lett.* **2018**, 20, 4627-4631.

(2*R*,3*S*,4*R*)-3,4-dihydroxy-2-(hydroxymethyl)-7-propyl-6-(4-(trifluoromethyl)phenyl)-2,3,4,6-tetrahydro-5*H*-pyrano[3,2-c]pyridin-5-one (6b)

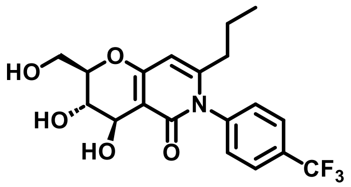

The product was obtained as a white solid (29.1 mg, 81 %) mp. 151-153°C. **[α]D^20** = +7.9 (c = 0.1 in MeOH). **^{1}H NMR** (300 MHz, DMSO-*d6*) δ 7.89 (d, *J* = 8.1 Hz, 2H), 7.52 (d, *J* = 7.2 Hz, 2H), 5.94 (s, 1H), 5.29 (m, 1H), 5.23 (m, 1H), 4.83 (m, 1H), 4.38 (m, 1H), 4.15 (dd, *J* = 9.0 Hz, 4.3 Hz, 1H), 3.78 (m, 1H), 3.71 (m, 2H), 2.12 (t, *J* = 7.3 Hz, 2H), 1.35 (sext, *J* = 7.2 Hz, 3H), 0.72 (t, *J* = 7.3 Hz, 3H). **^{13}C NMR** (75 MHz, ($CDCl_3$) δ 163.6, 160.7, 148.5, 141.7, 130.2, 130.1, 128.8 (q, *J* = 31.9 Hz, C-F), 126.2 (q, *J* = 3.6 Hz, C-F), 123.9 (q, *J* = 270 Hz, C-F), 104.9, 98.9, 81.0, 67.1, 64.0, 60.2, 34.4, 20.2, 13.2. **IR** (ν, cm^{-1}) = 3257; 2834; 1585; 1510; 1406; 1279; 1069; 1007; 983. **HRMS (ESI)** *m/z*: $[M + Na]^+$ Calcd for $C_{19}H_{20}F_3NO_5Na$ 422.1191; Found 422.1231.

3. NMR (^{1}H, ^{13}C) Spectra of Products

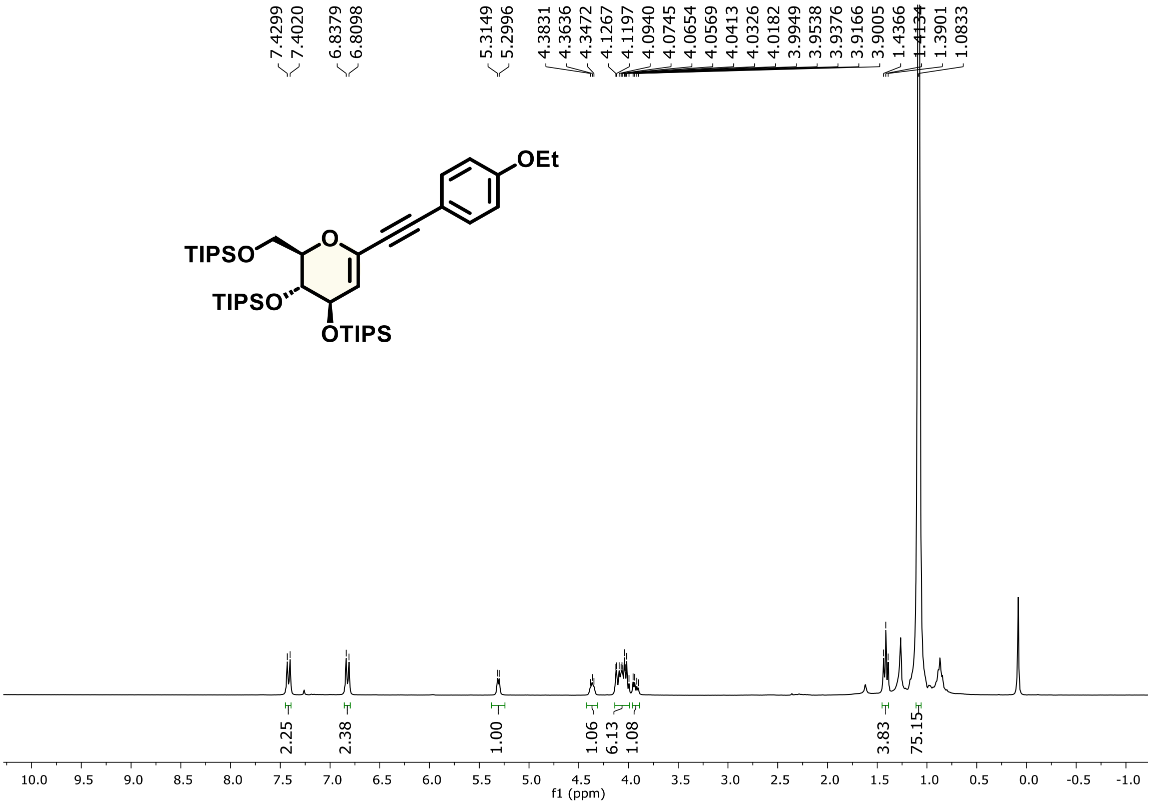

Figure S1. ^{1}H NMR spectra (300 MHz, $CDCl_3$) of **2c**

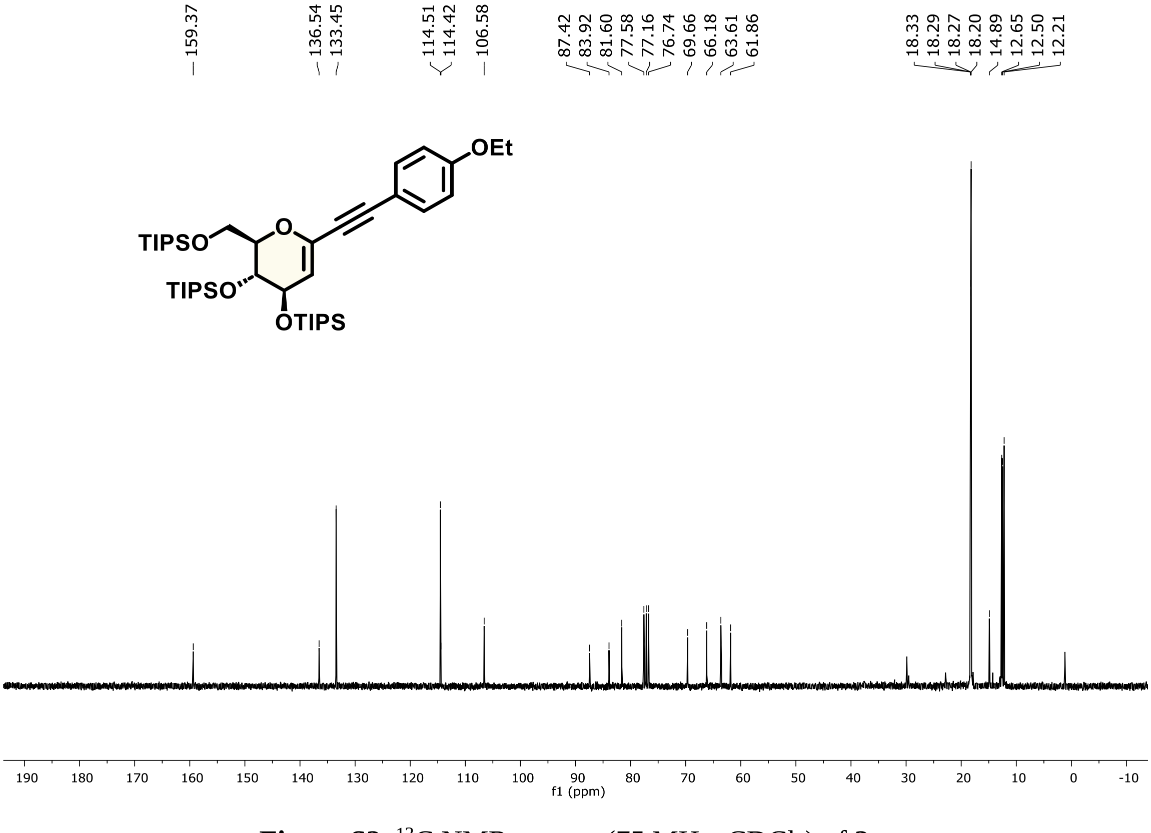

Figure S2. ^{13}C NMR spectra (75 MHz, $CDCl_3$) of **2c**

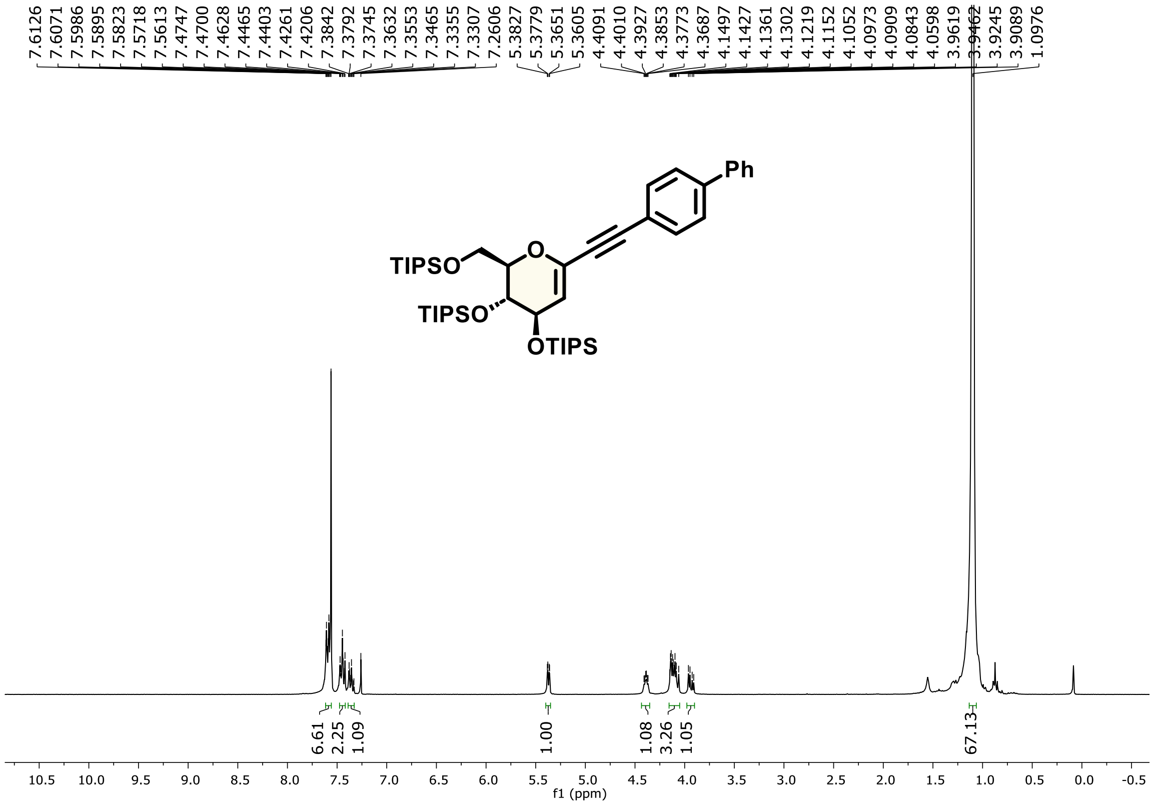

Figure S3. ^{1}H NMR spectra (300 MHz, $CDCl_3$) of **2d**

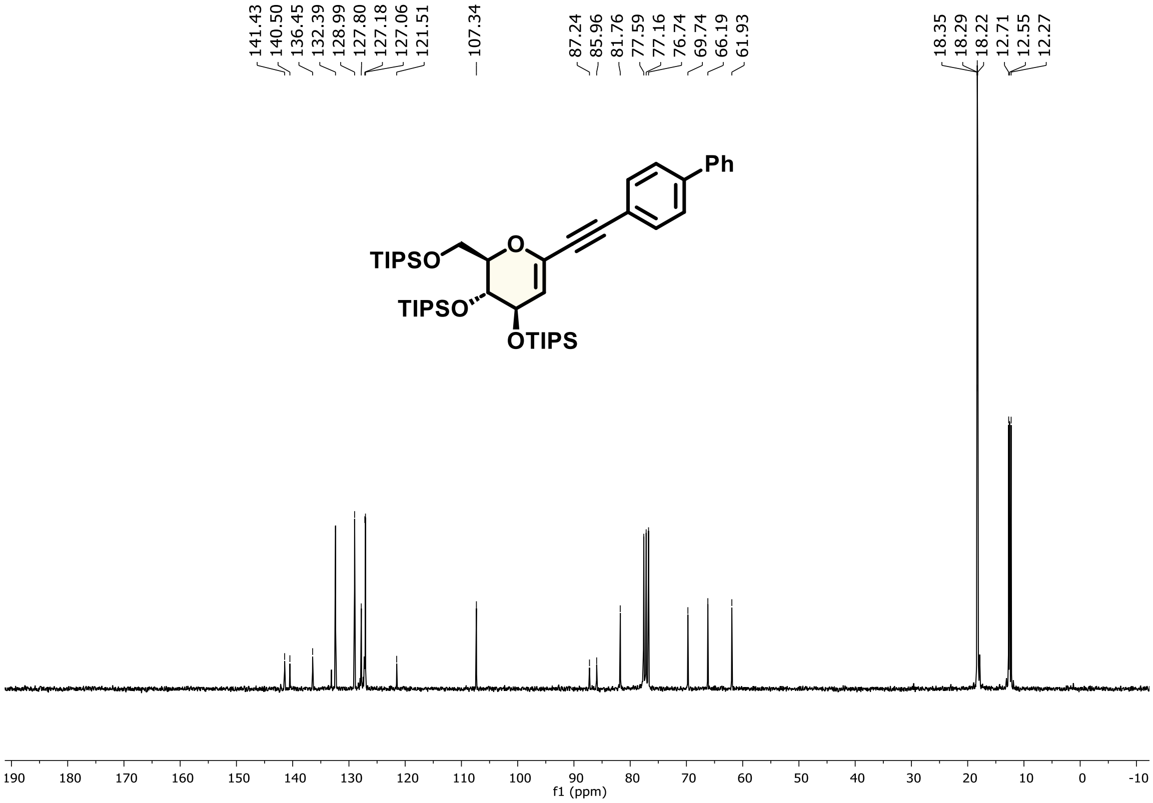

Figure S4. ^{13}C NMR spectra (75 MHz, $CDCl_3$) of **2d**

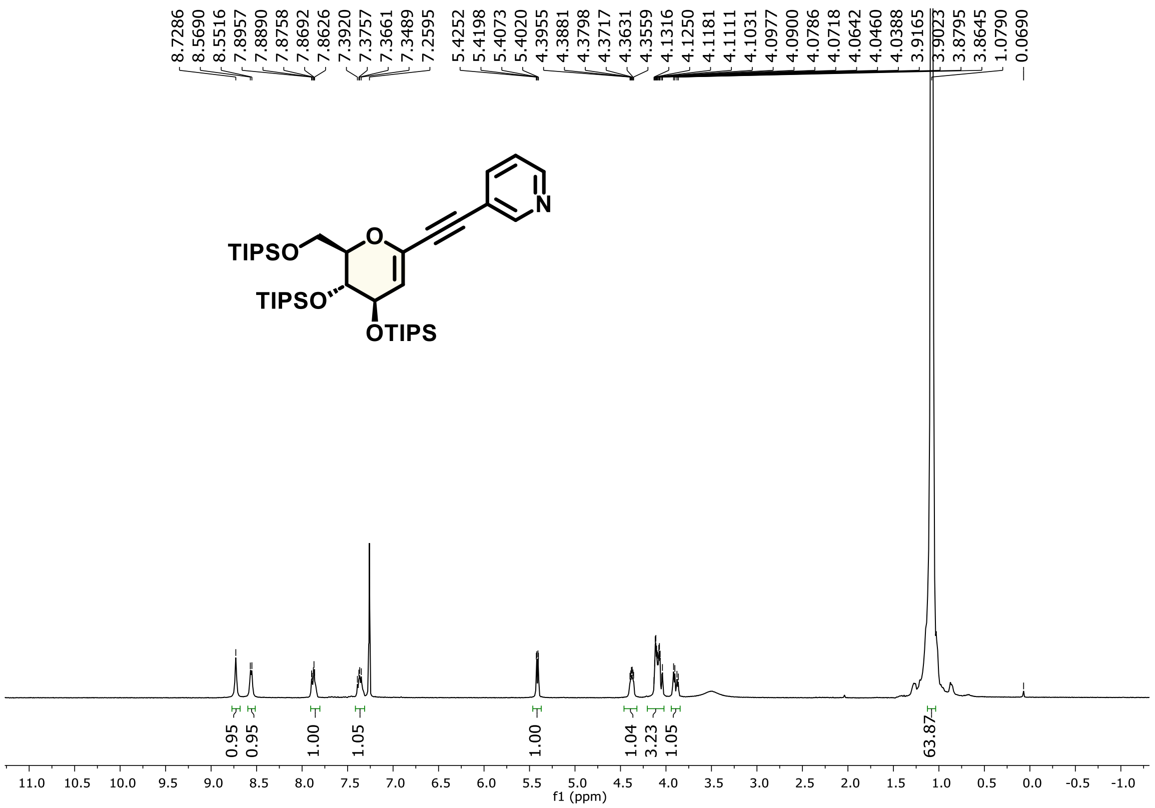

Figure S5. ^{1}H NMR spectra (300 MHz, $CDCl_3$) of **2e**

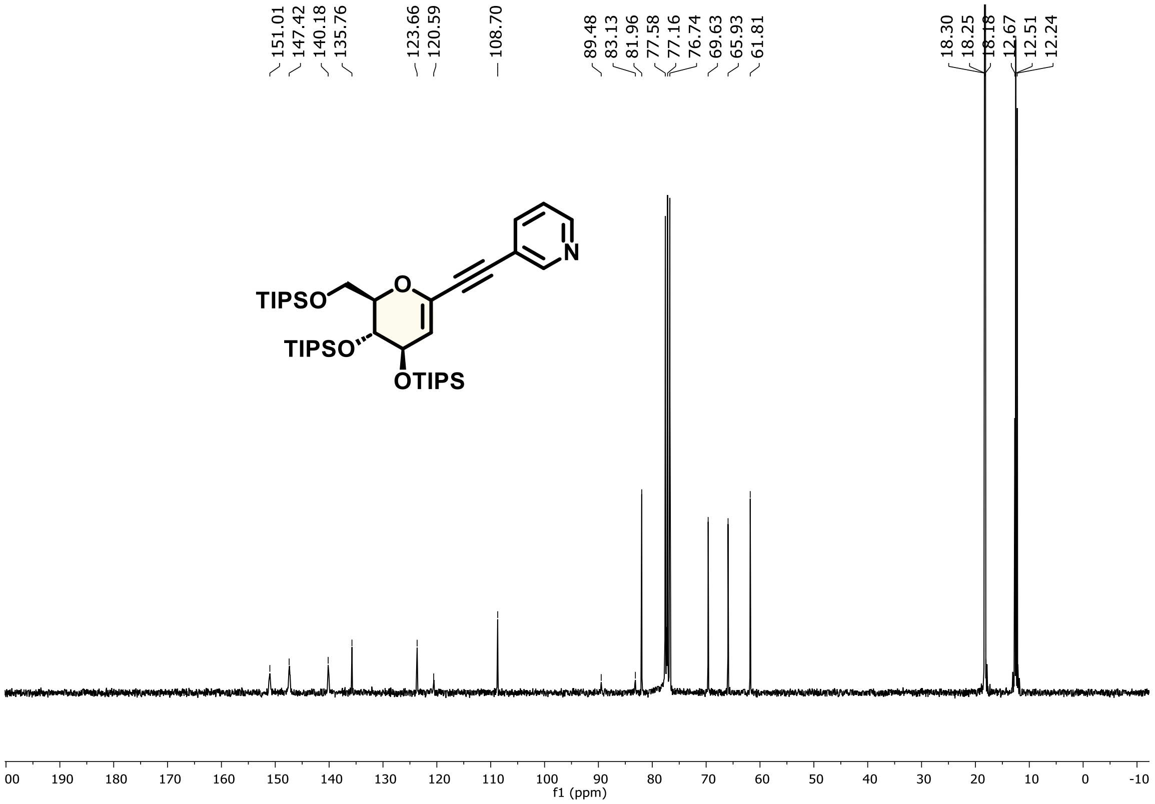

Figure S6. ^{13}C NMR spectra (75 MHz, $CDCl_3$) of **2e**

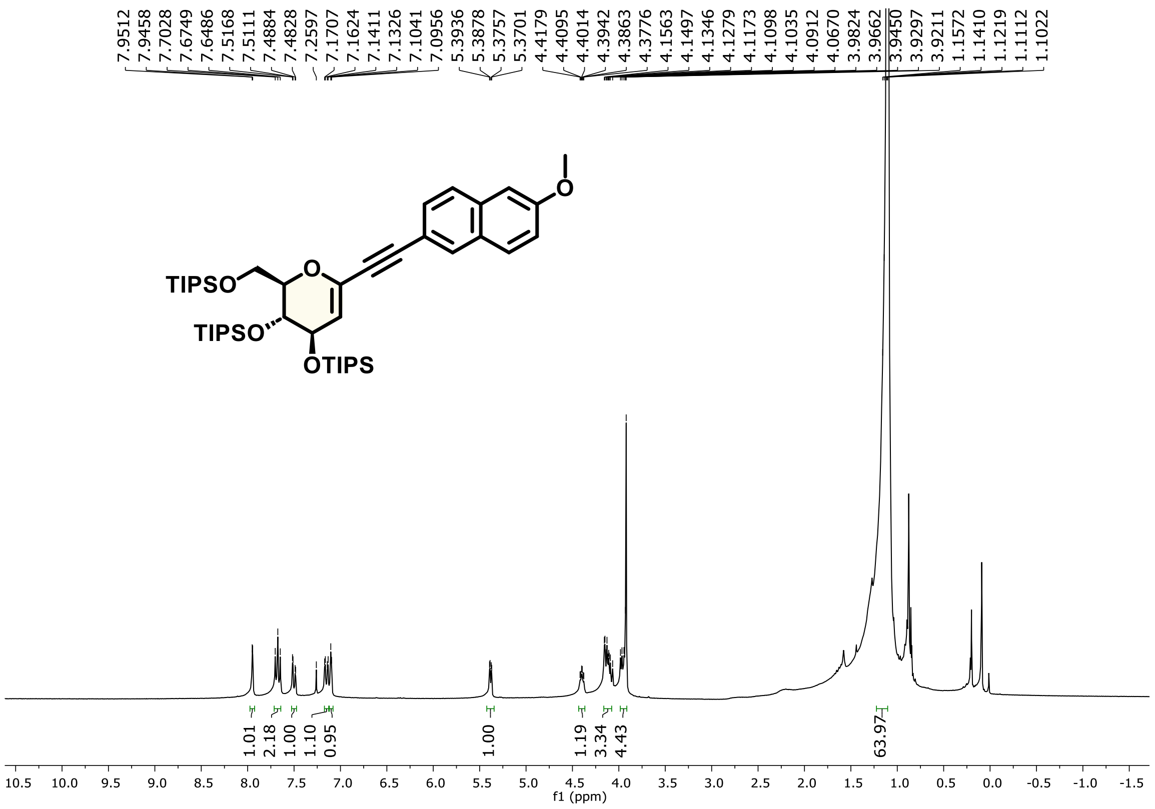

Figure S7. ^{1}H NMR spectra (300 MHz, $CDCl_3$) of **2f**

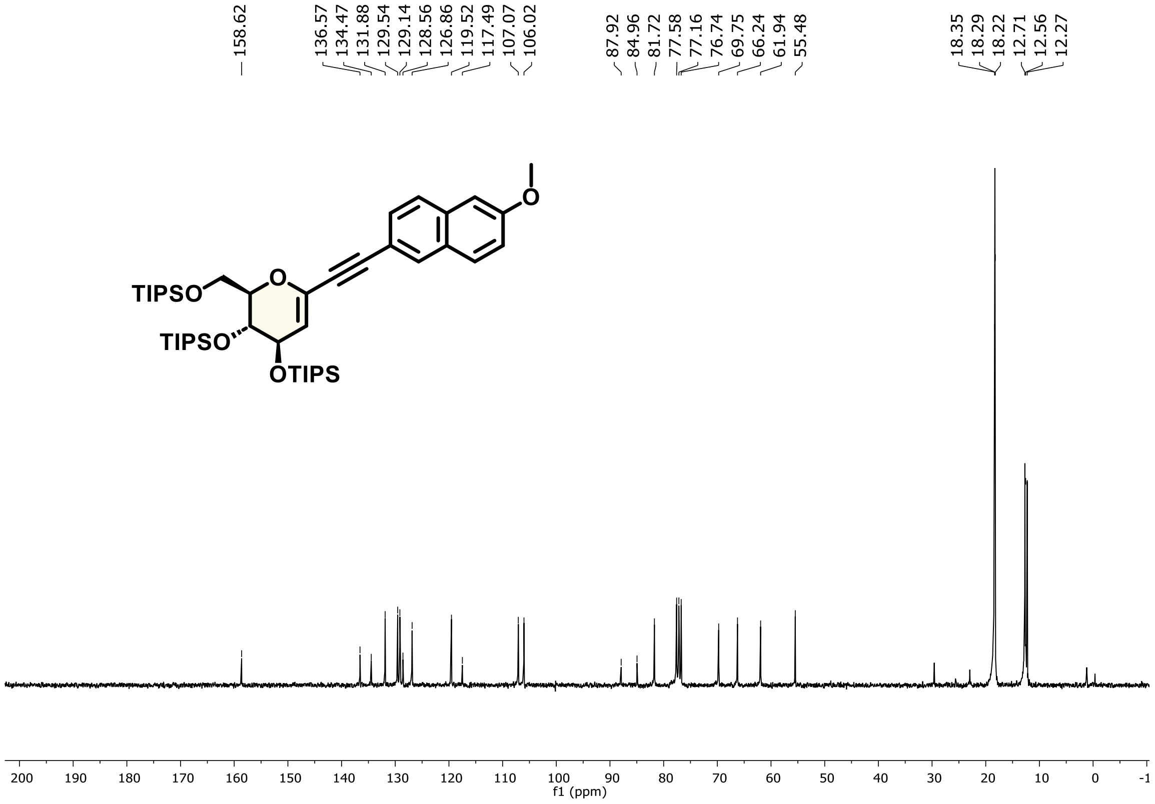

Figure S8. ^{13}C NMR spectra (75 MHz, $CDCl_3$) of **2f**

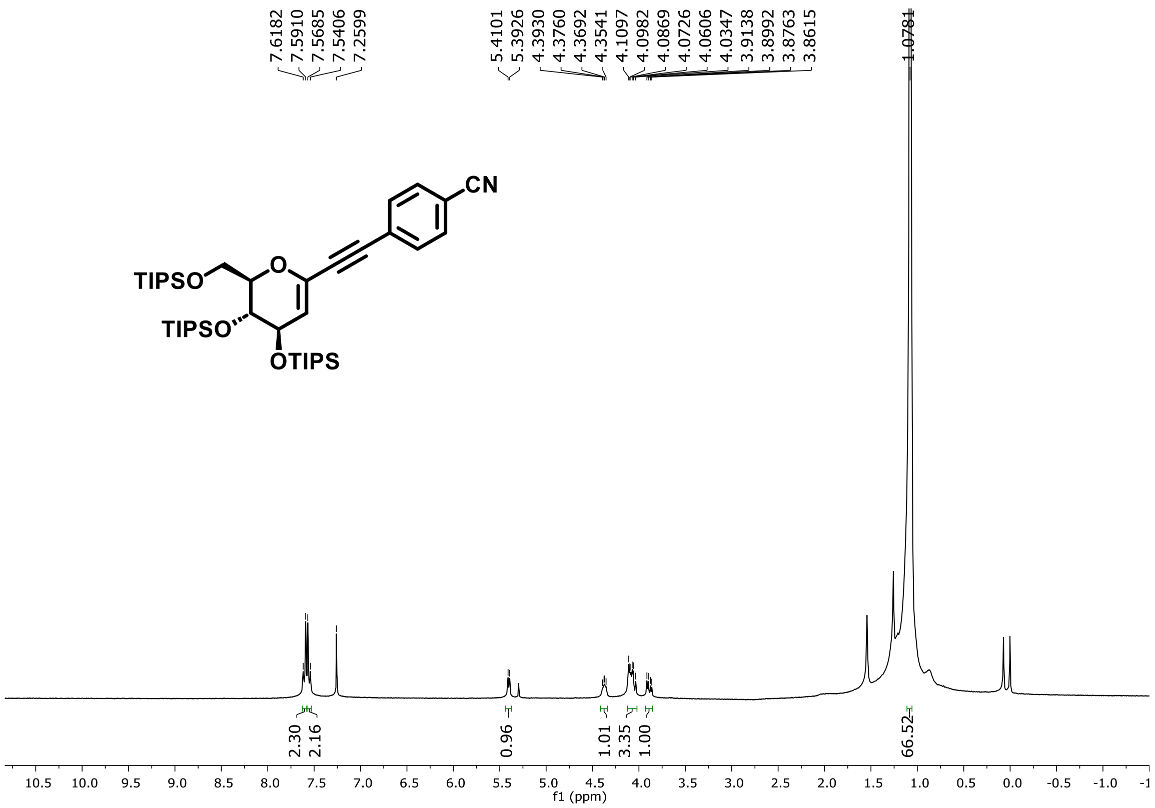

Figure S9. ^{1}H NMR spectra (300 MHz, $CDCl_3$) of **2g**

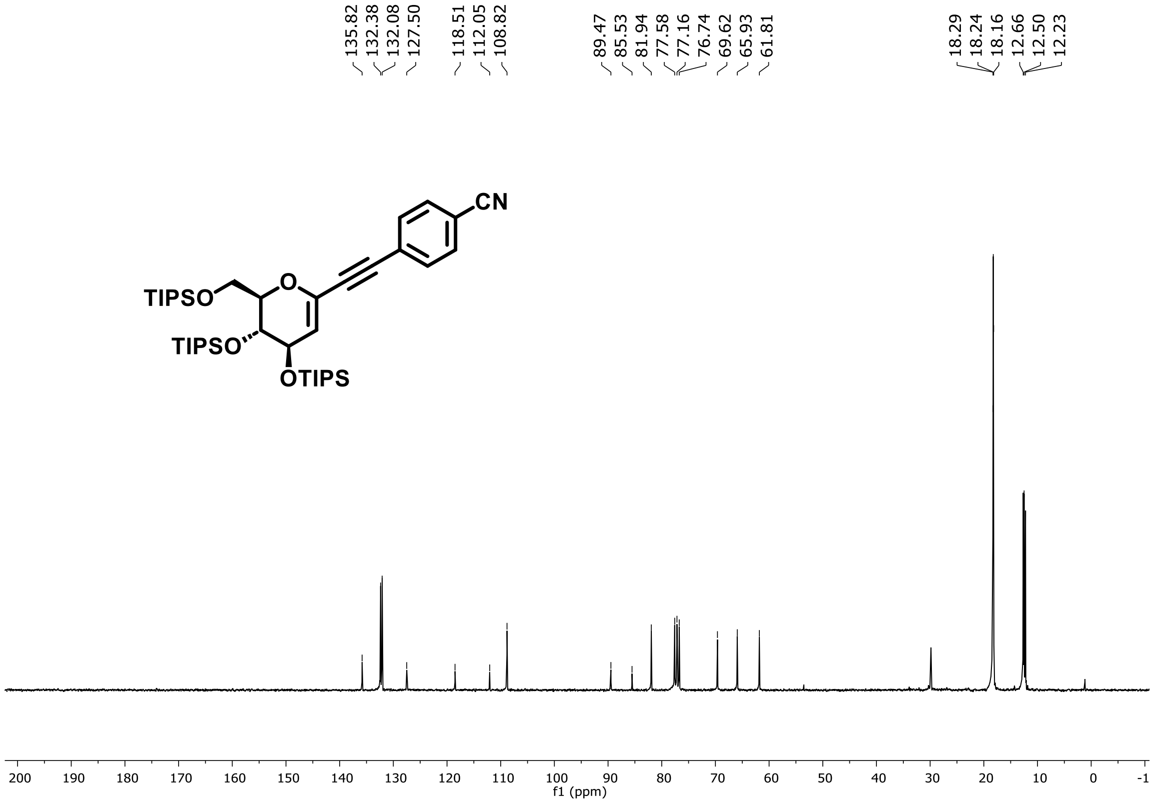

Figure S10. ^{13}C NMR spectra (75 MHz, $CDCl_3$) of **2g**

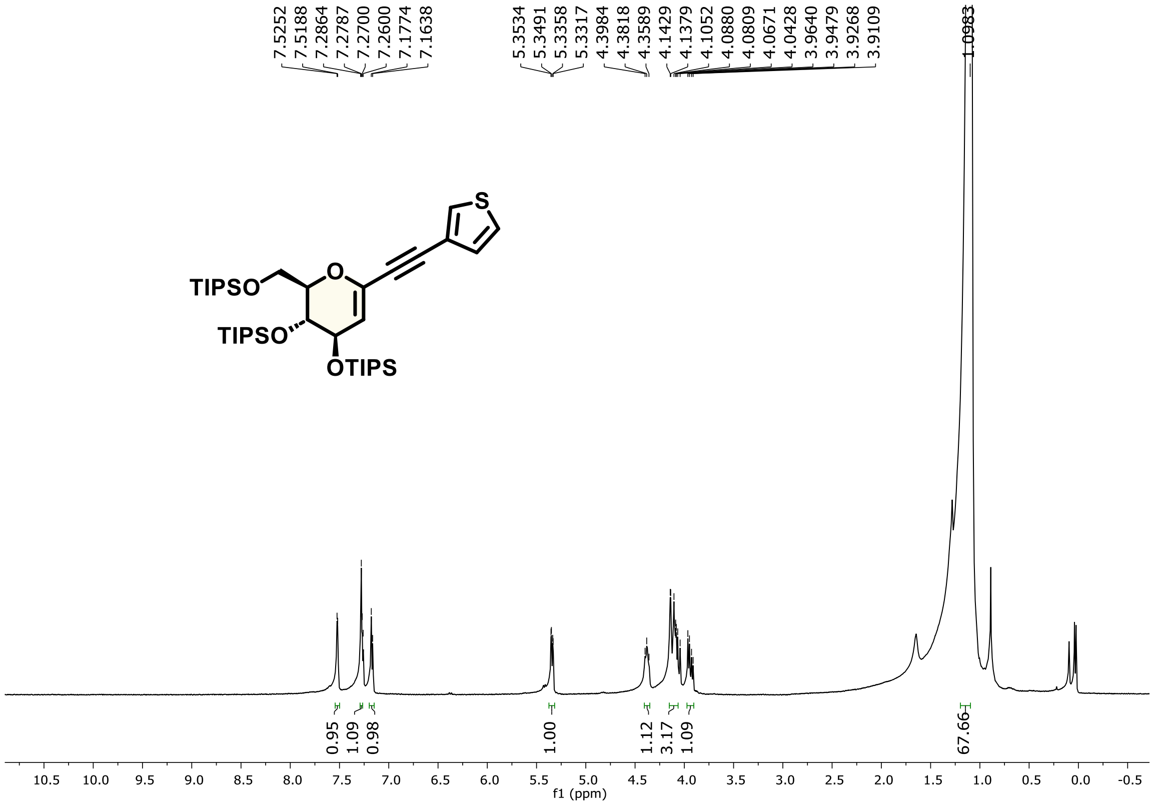

Figure S11. ^{1}H NMR spectra (300 MHz, $CDCl_3$) of **2h**

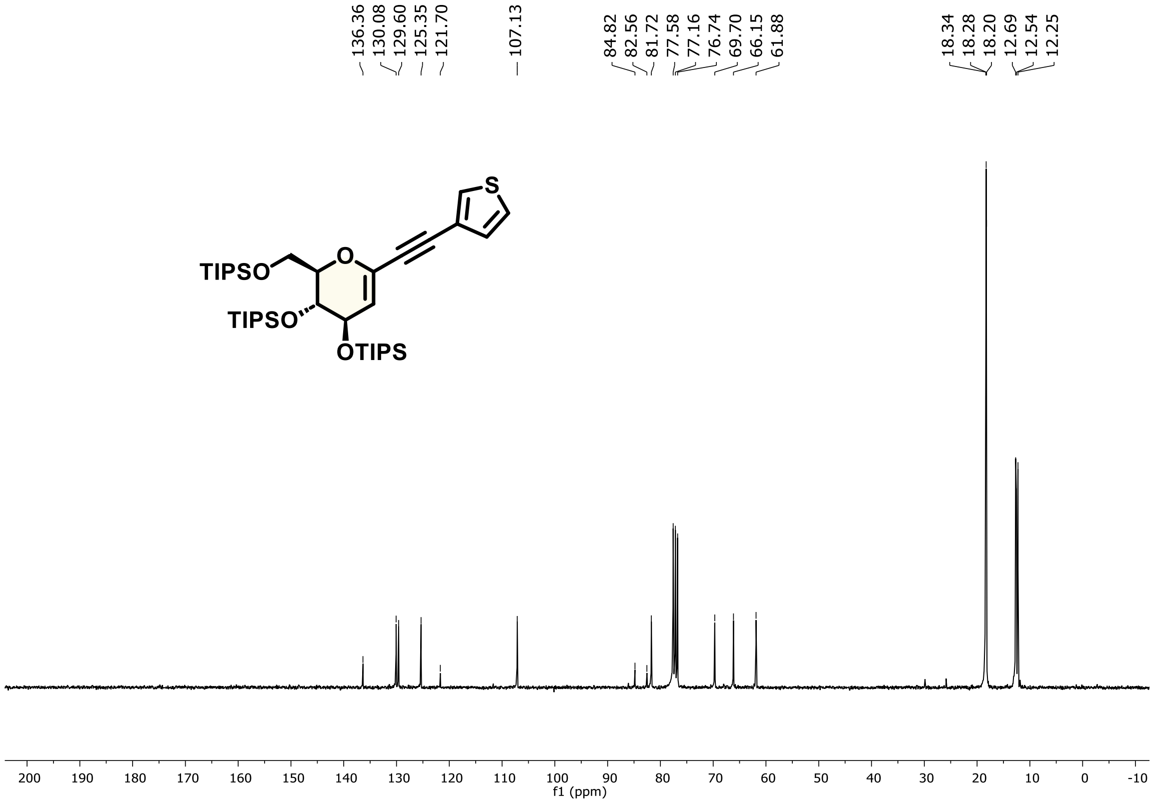

Figure S12. ^{13}C NMR spectra (75 MHz, $CDCl_3$) of **2h**

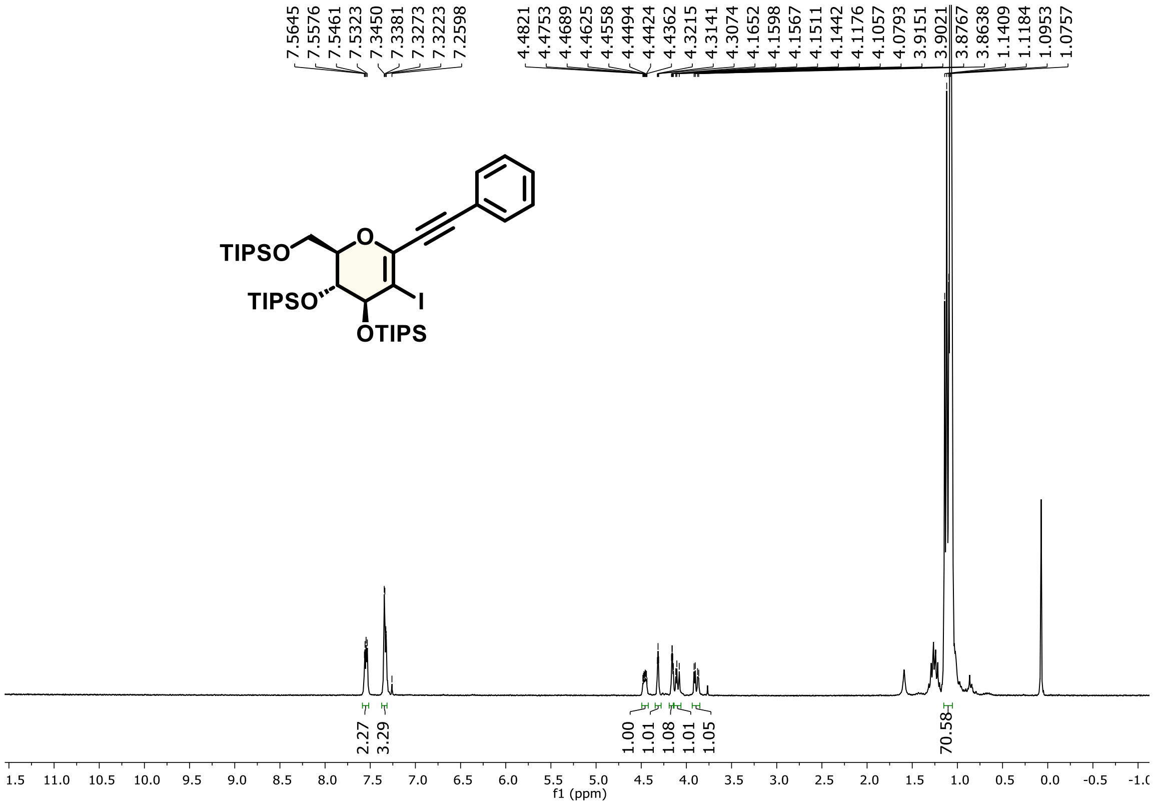

Figure S13. ^{1}H NMR spectra (300 MHz, $CDCl_3$) of **3a**

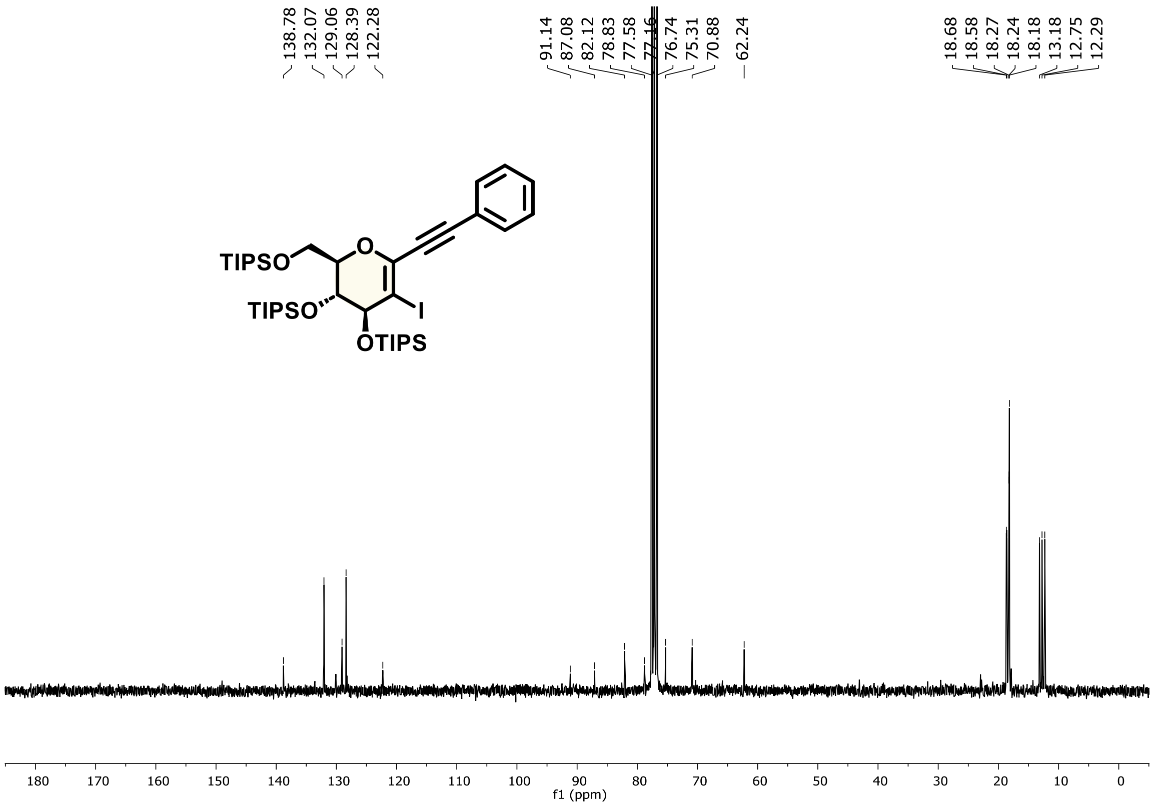

Figure S14. ^{13}C NMR spectra (75 MHz, $CDCl_3$) of **3a**

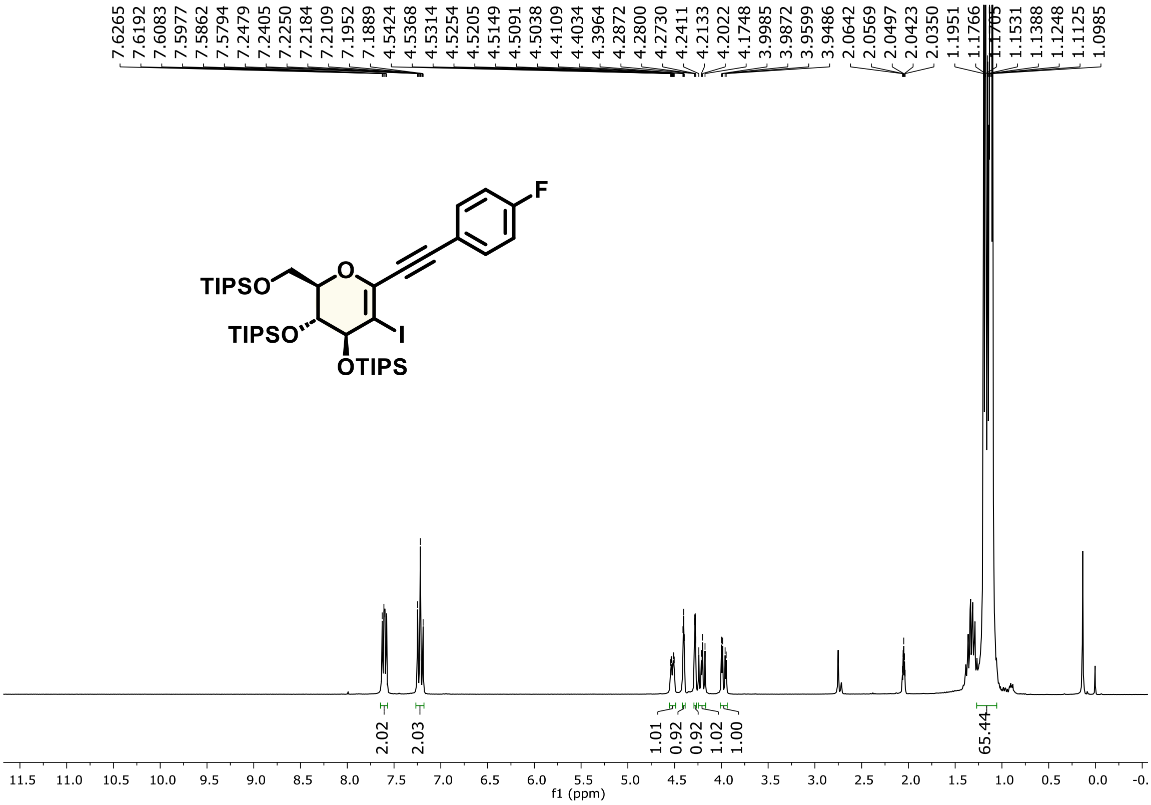

Figure S15. ^{1}H NMR spectra (300 MHz, $(CD_3)_2CO$) of **3b**

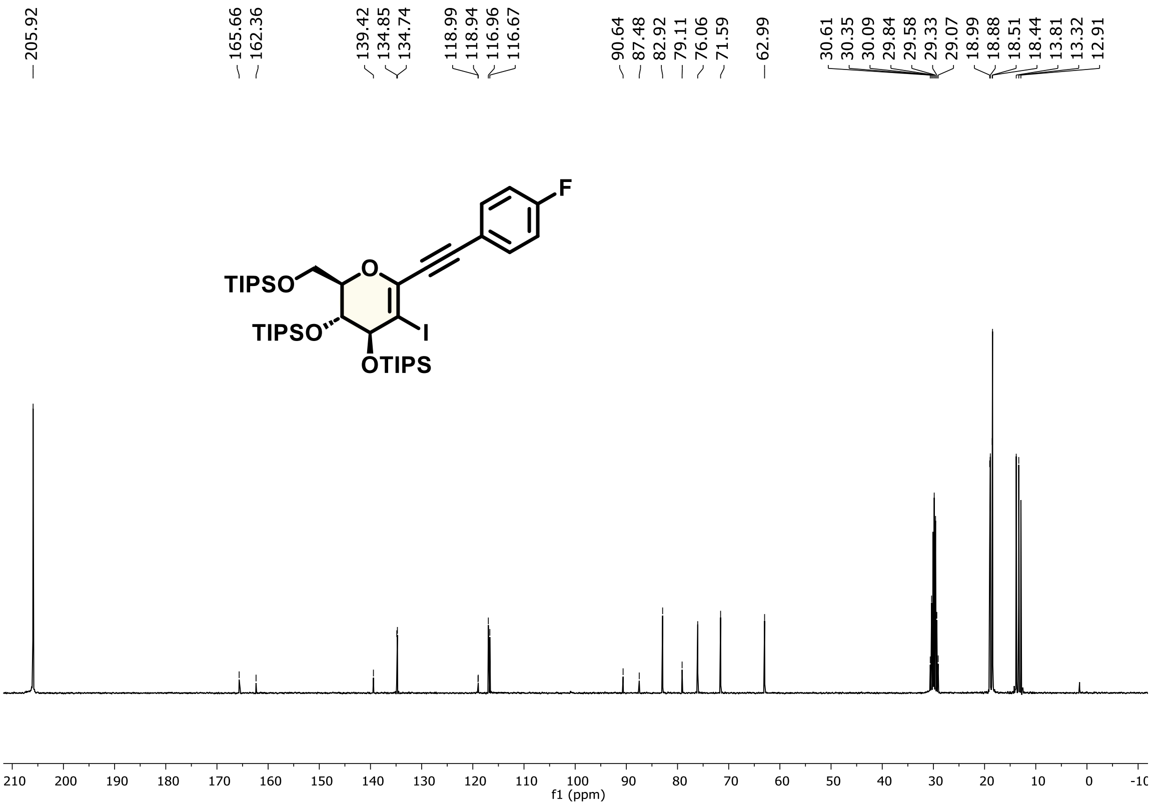

Figure S16. ^{13}C NMR spectra (75 MHz, $(CD_3)_2CO$) of **3b**

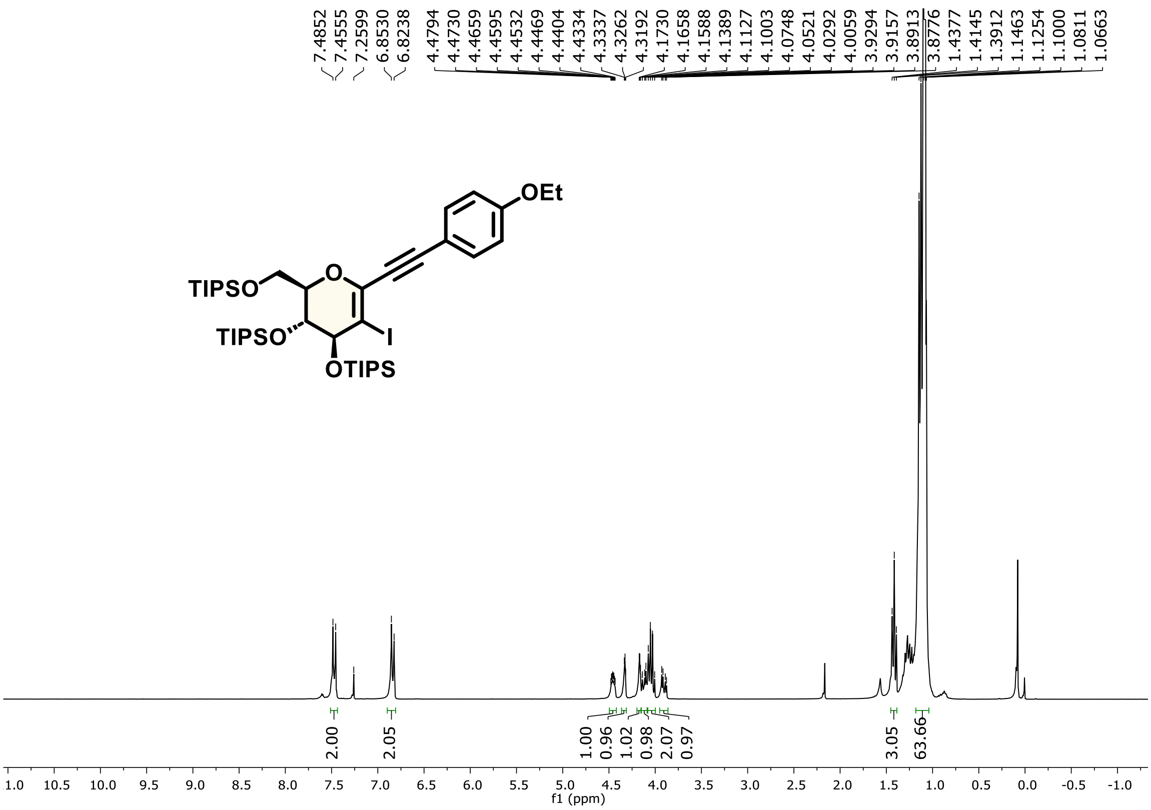

Figure S17. ^{1}H NMR spectra (300 MHz, $CDCl_3$) of **3c**

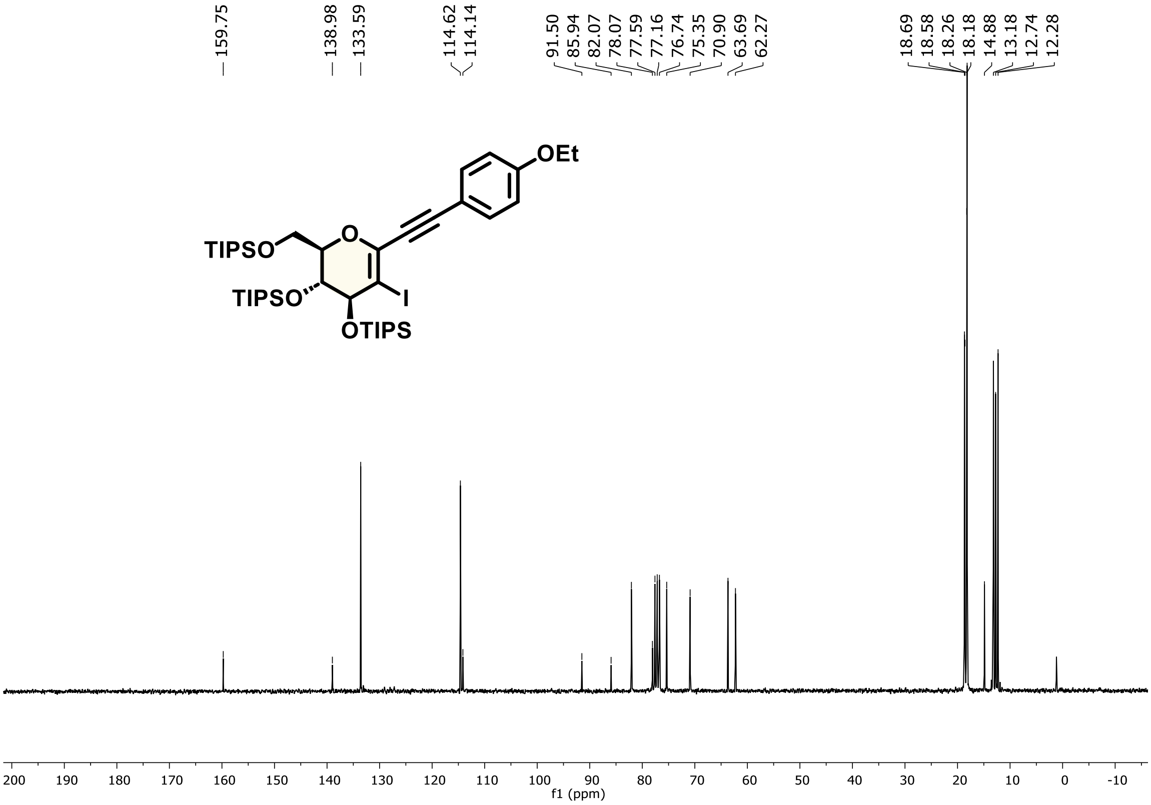

Figure S18. ^{13}C NMR spectra (75 MHz, $CDCl_3$) of **3c**

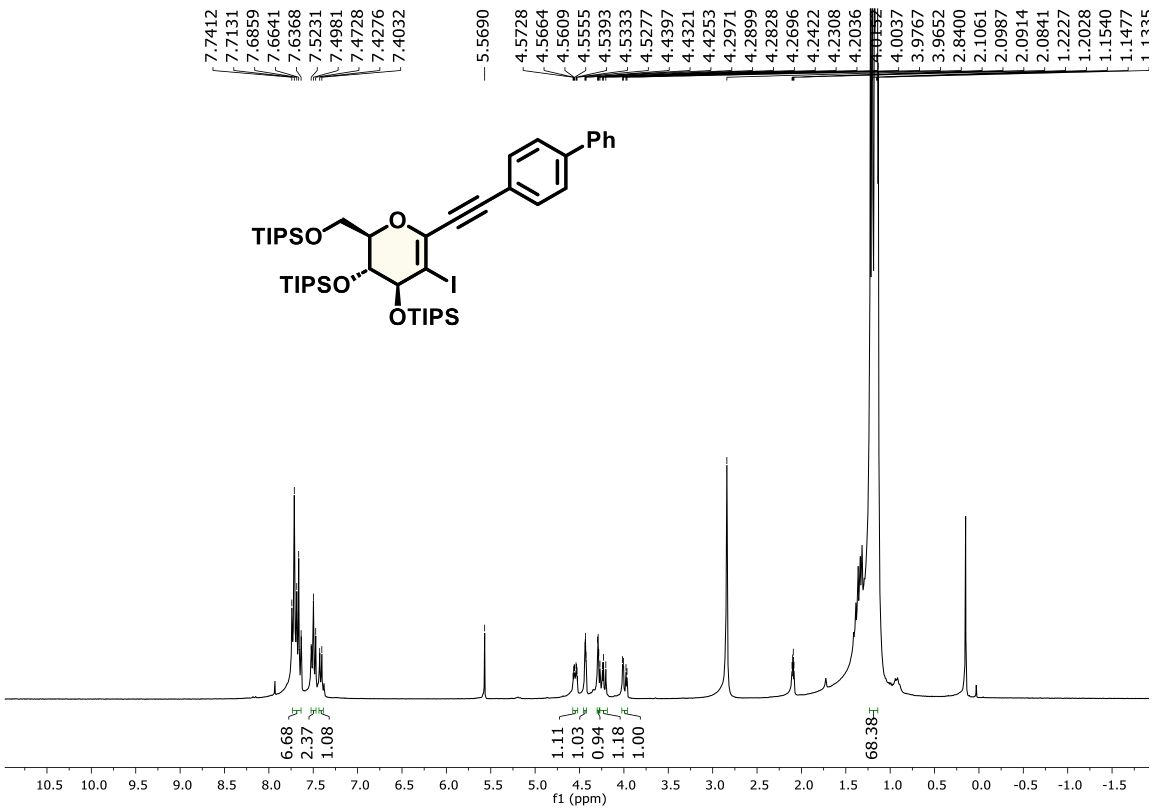

Figure S19. ^{1}H NMR spectra (300 MHz, $(CD_3)_2CO$) of **3d**

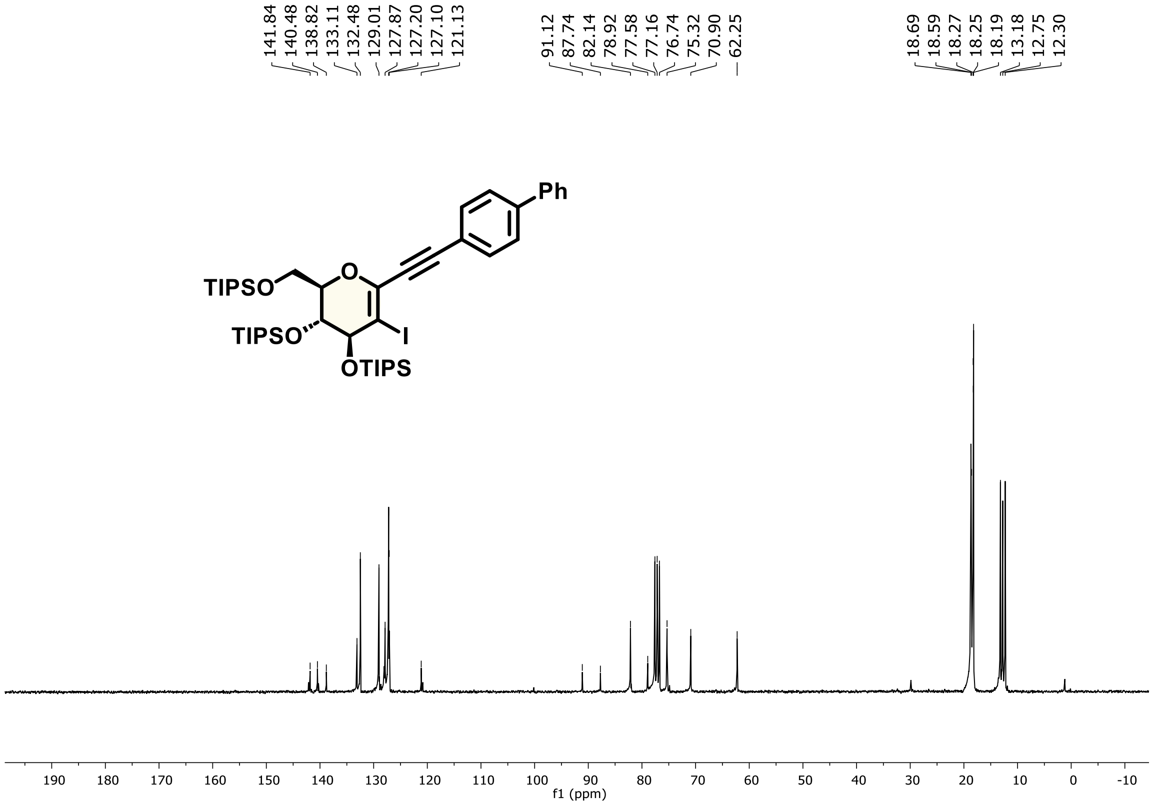

Figure S20. ^{13}C NMR spectra (75 MHz, $CDCl_3$) of **3d**

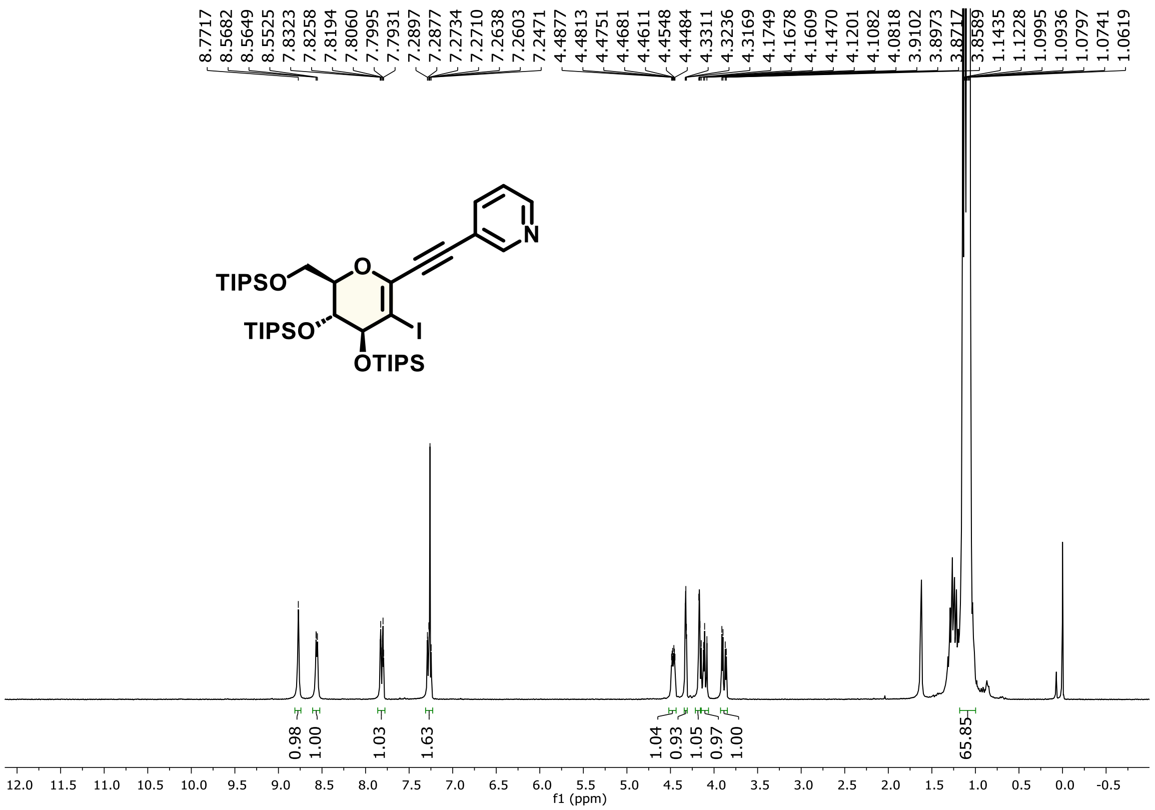

Figure S21. ^{1}H NMR spectra (300 MHz, $CDCl_3$) of **3e**

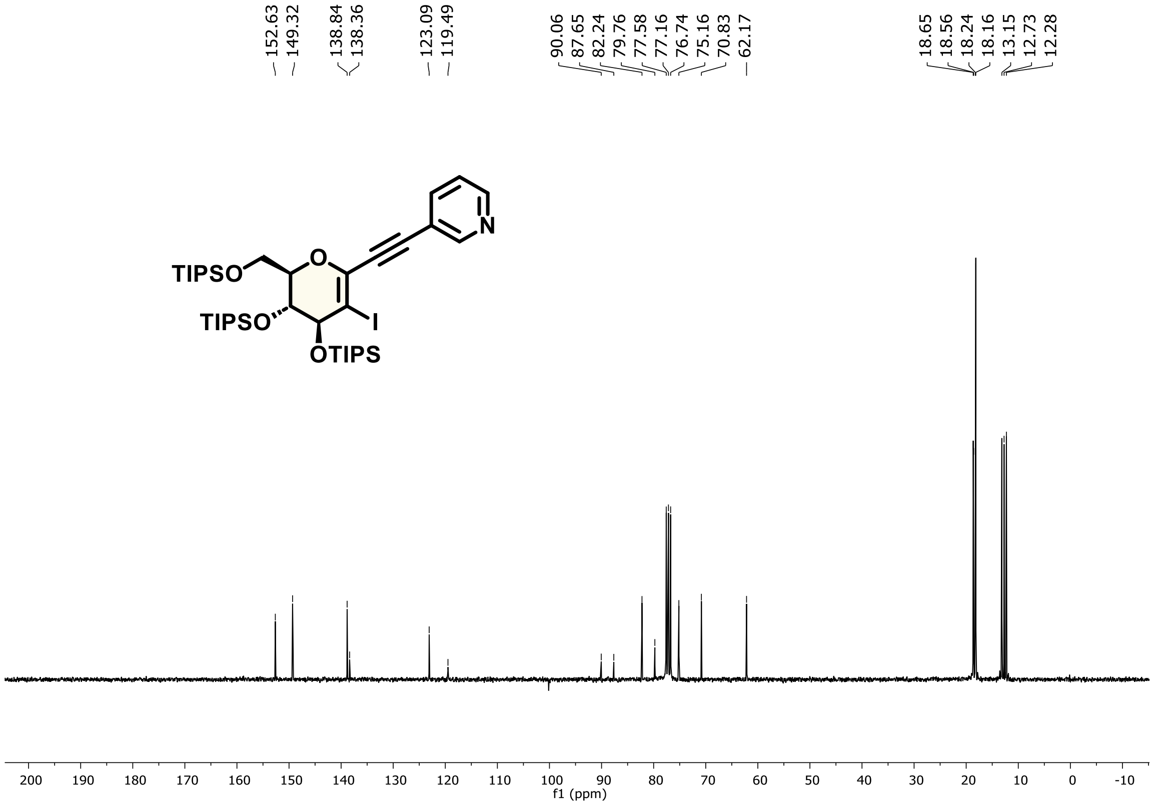

Figure S22. ^{13}C NMR spectra (75 MHz, $CDCl_3$) of **3e**

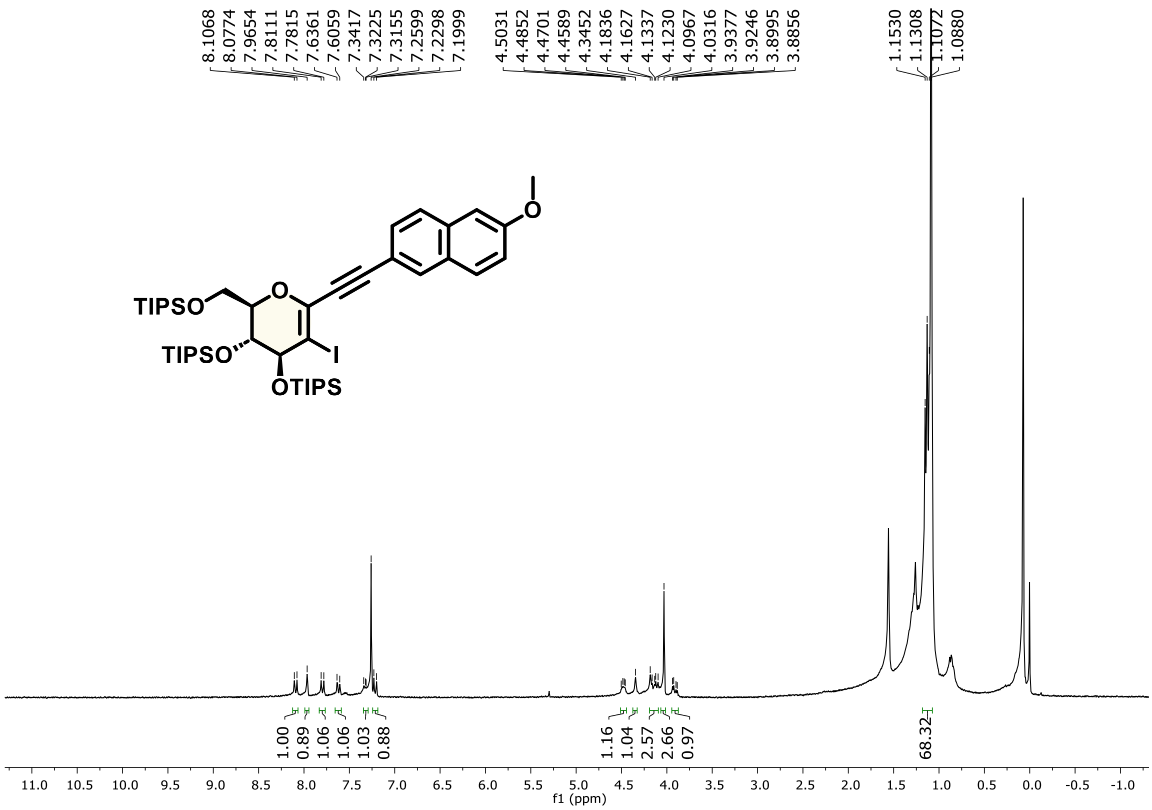

Figure S23. ^{1}H NMR spectra (300 MHz, $CDCl_3$) of **3f**

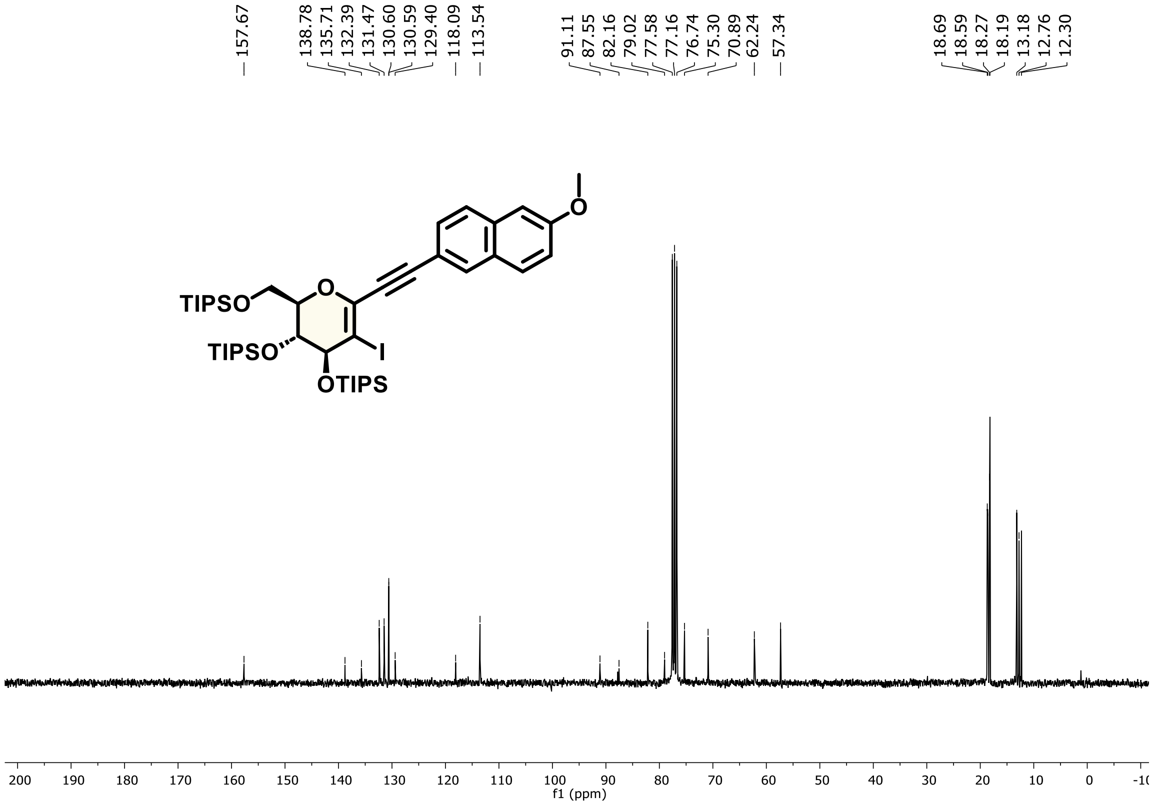

Figure S24. ^{13}C NMR spectra (75 MHz, $CDCl_3$) of **3f**

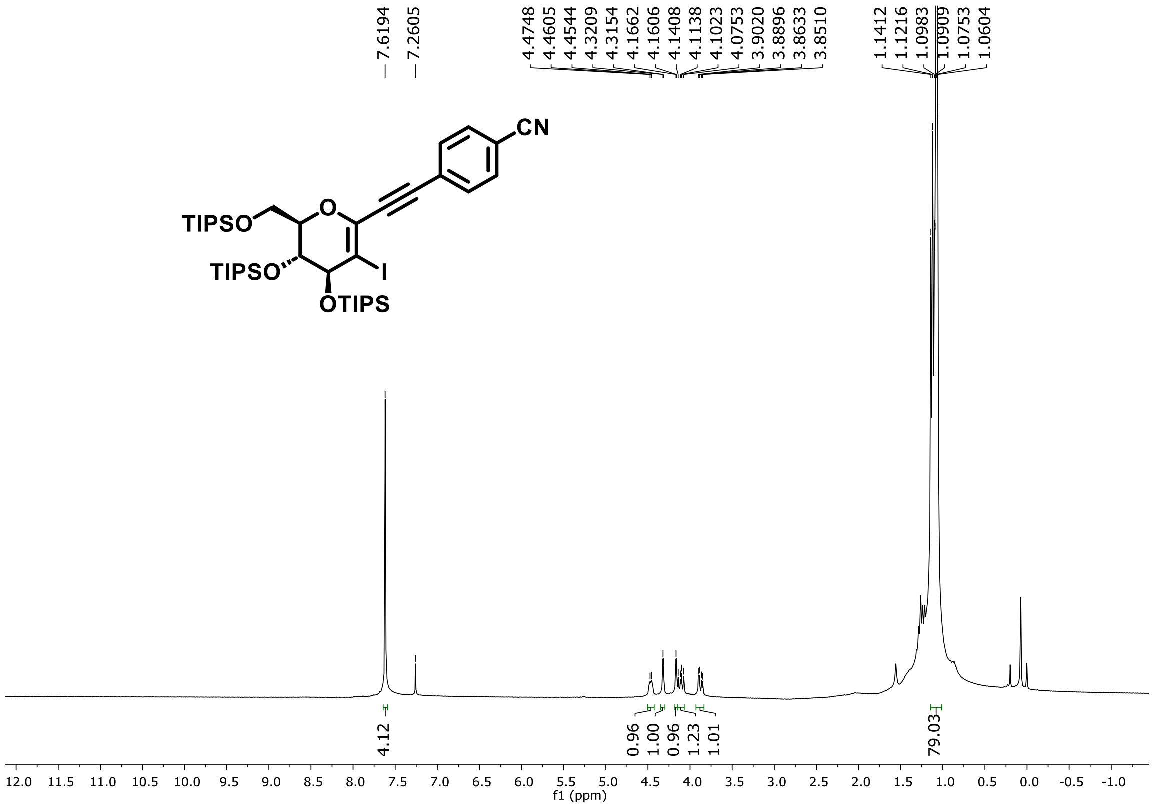

Figure S25. ^{1}H NMR spectra (300 MHz, $CDCl_3$) of **3g**

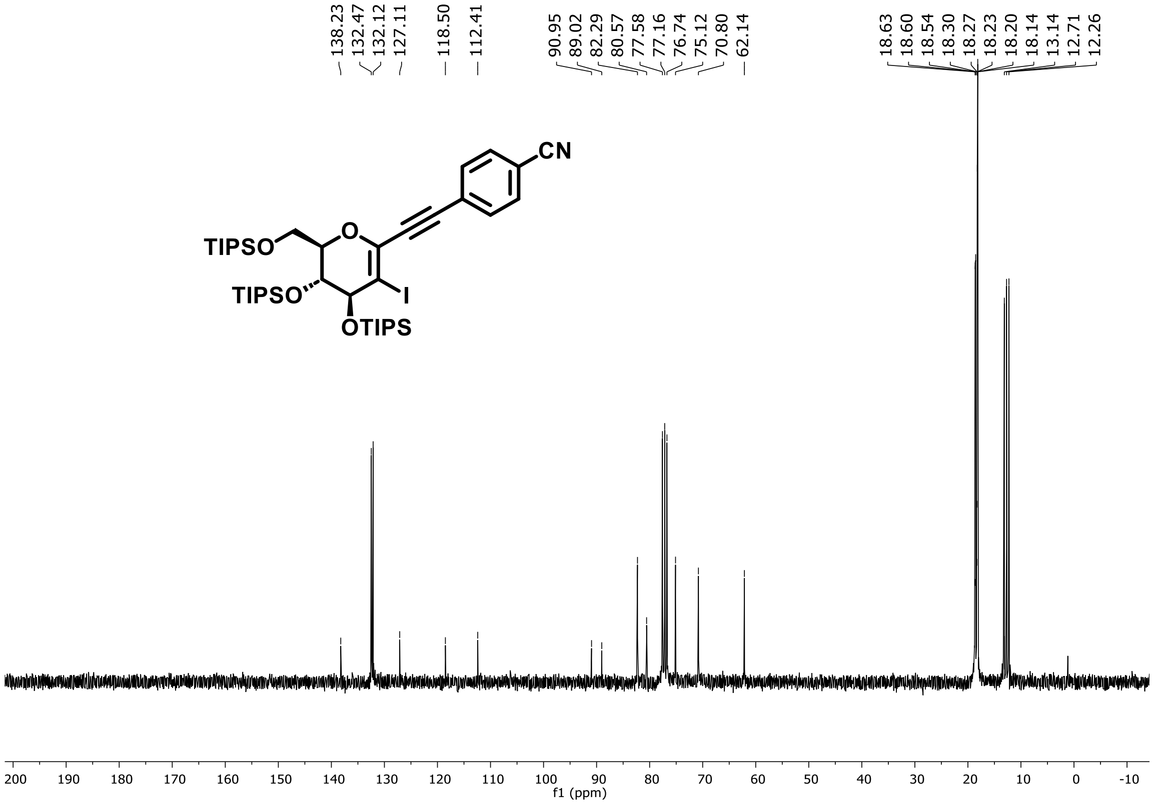

Figure S26. ^{13}C NMR spectra (75 MHz, $CDCl_3$) of **3g**

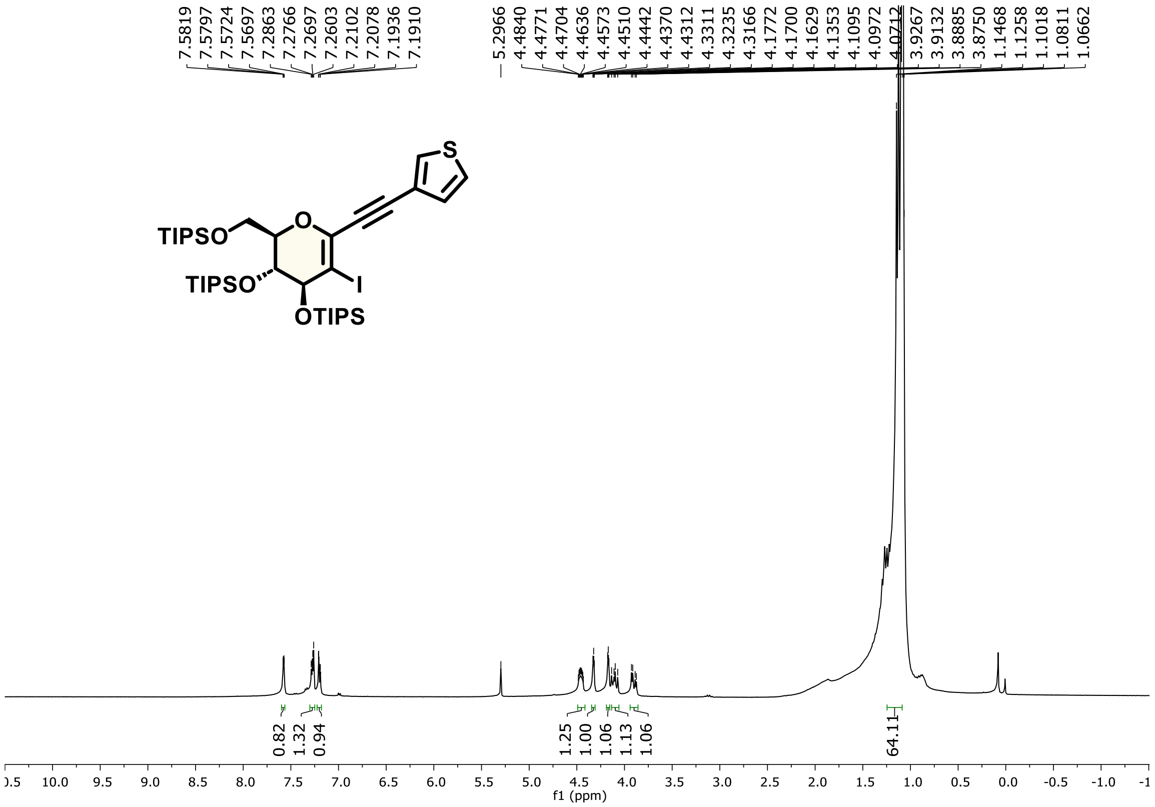

Figure S27. ^{1}H NMR spectra (300 MHz, $CDCl_3$) of **3h**

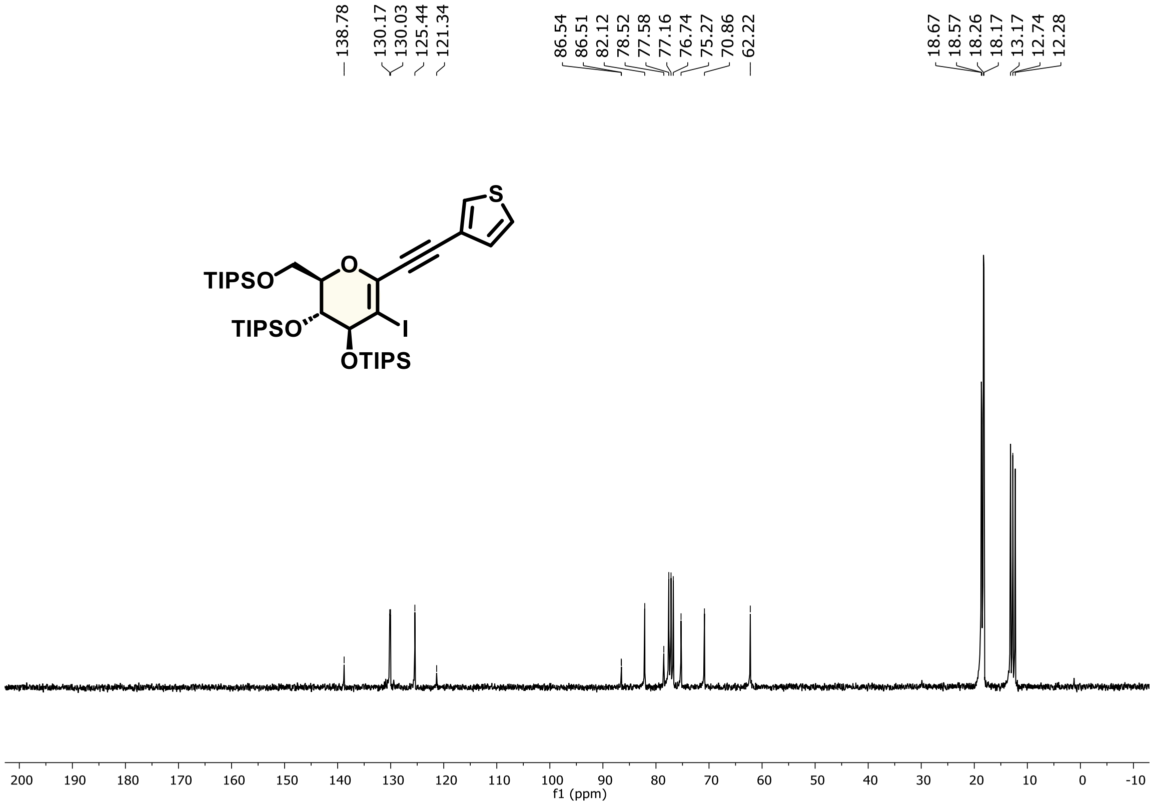

Figure S28. ^{13}C NMR spectra (75 MHz, $CDCl_3$) of **3h**

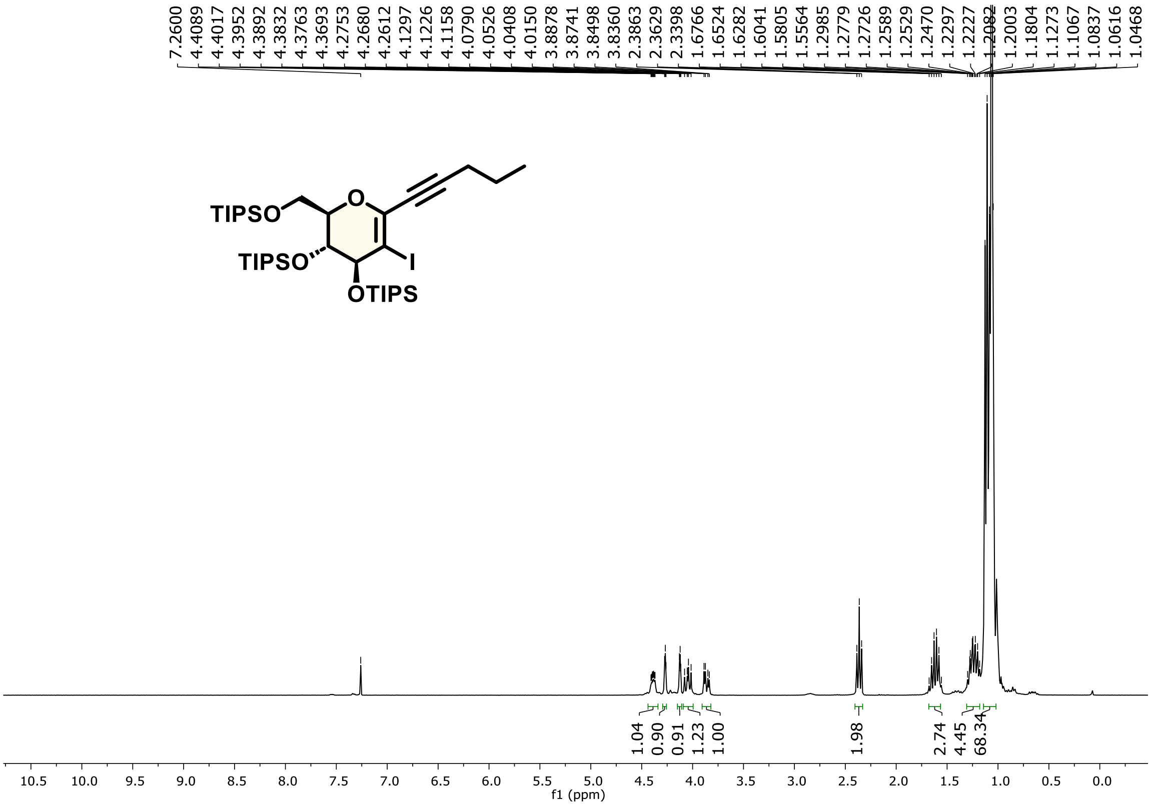

Figure S29. ^{1}H NMR spectra (300 MHz, $CDCl_3$) of **3i**

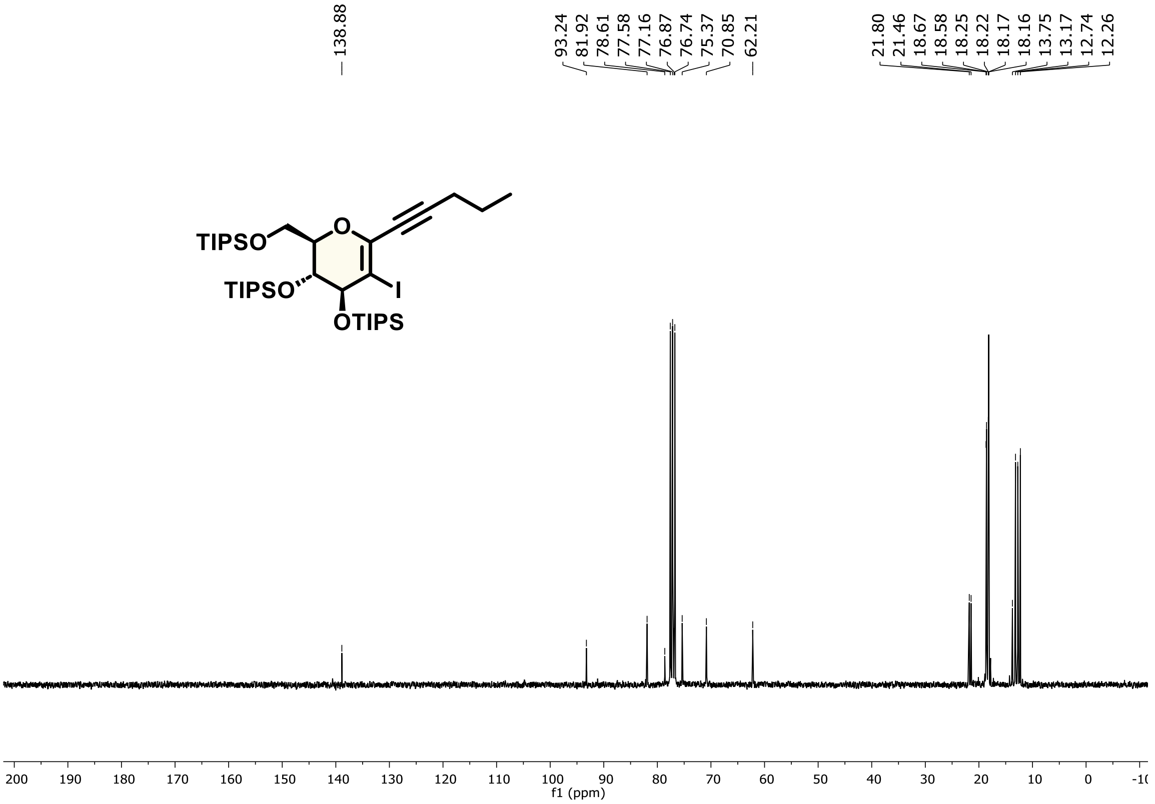

Figure S30. ^{13}C NMR spectra (75 MHz, $CDCl_3$) of **3i**

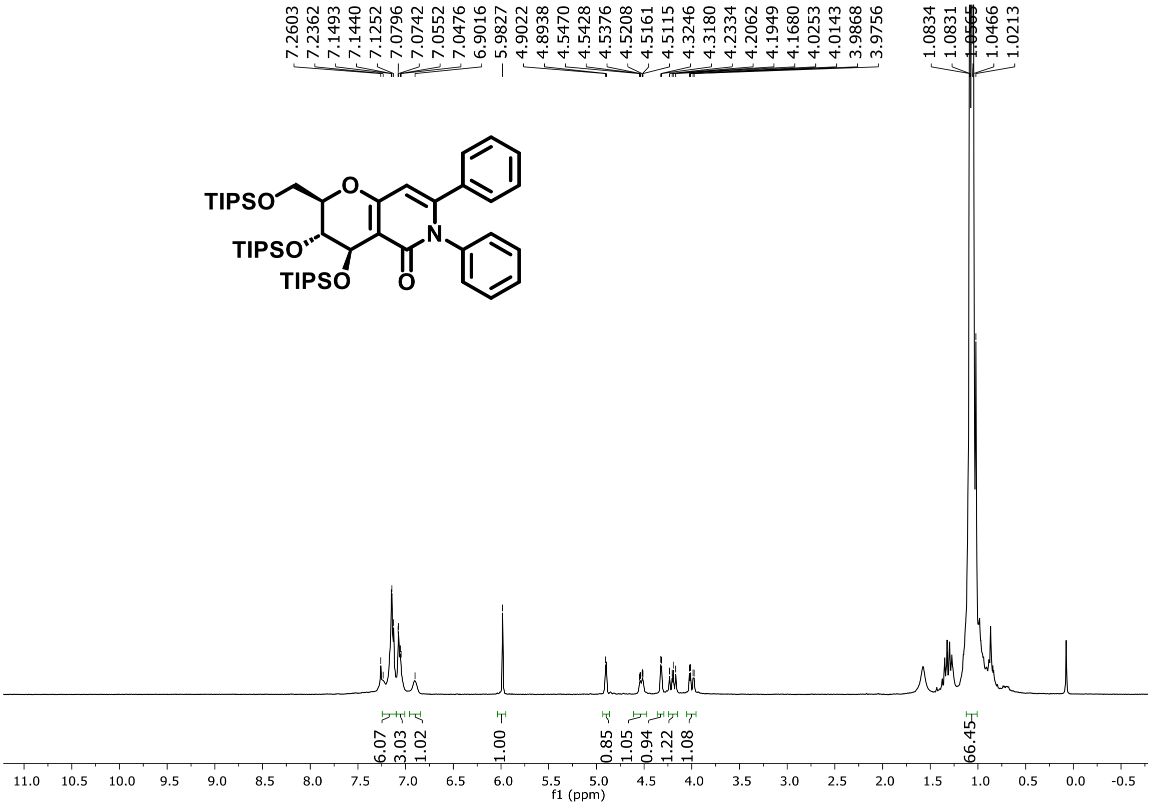

Figure S31. ^{1}H NMR spectra (300 MHz, $CDCl_3$) of **4a**

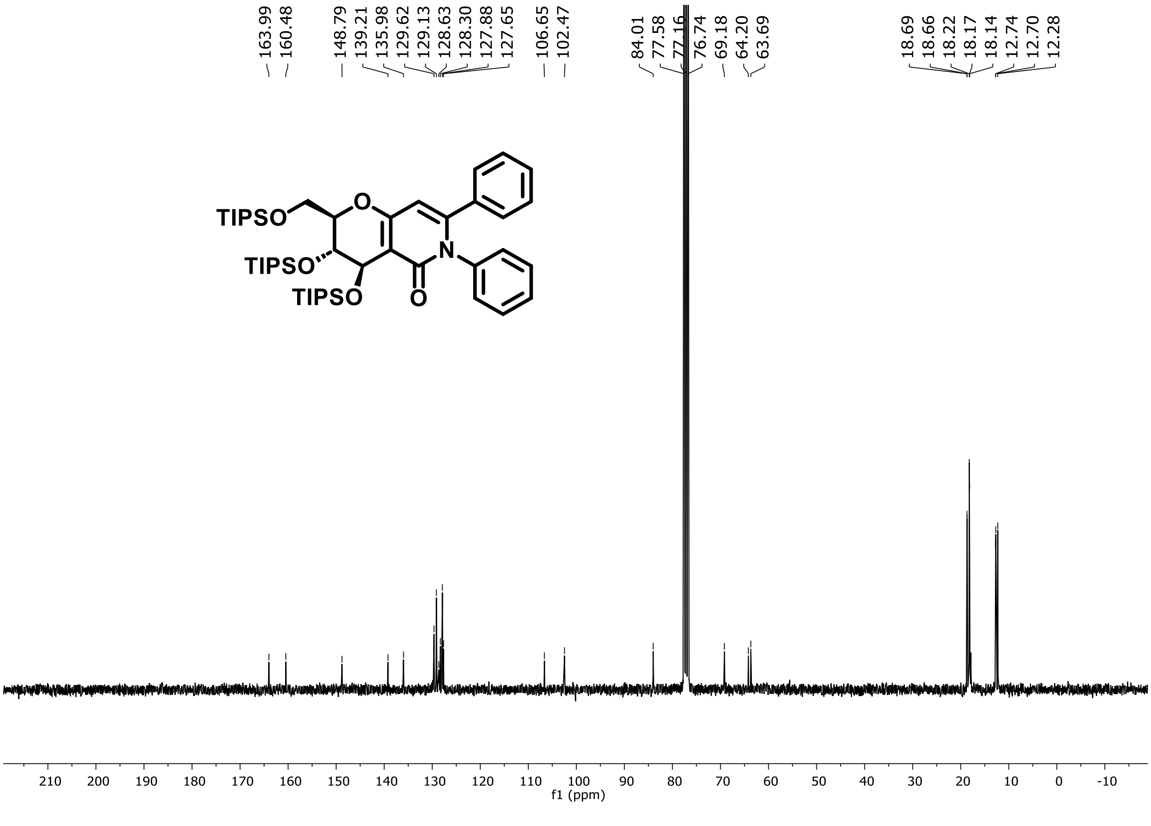

Figure S32. ^{13}C NMR spectra (75 MHz, $CDCl_3$) of **4a**

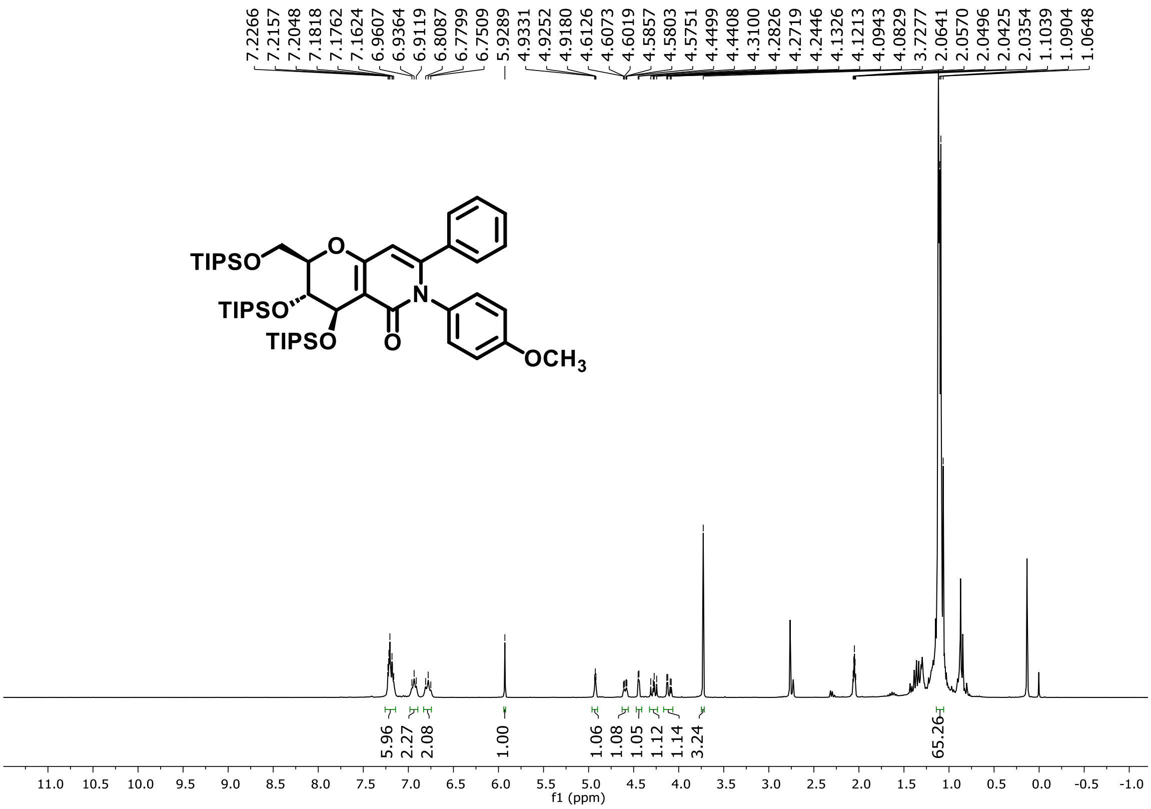

Figure S33. ^{1}H NMR spectra (300 MHz, $(CD_3)_2CO$) of **4b**

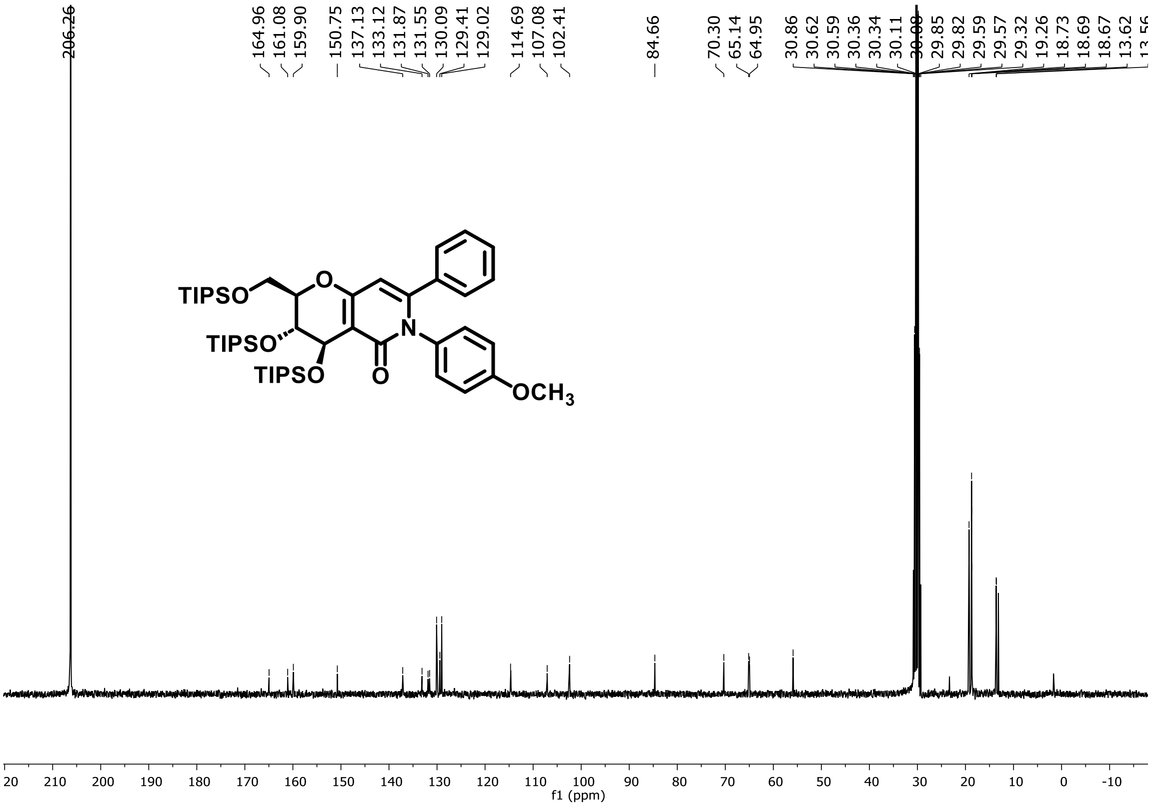

Figure S34. ^{13}C NMR spectra (75 MHz, $(CD_3)_2CO$) of **4b**

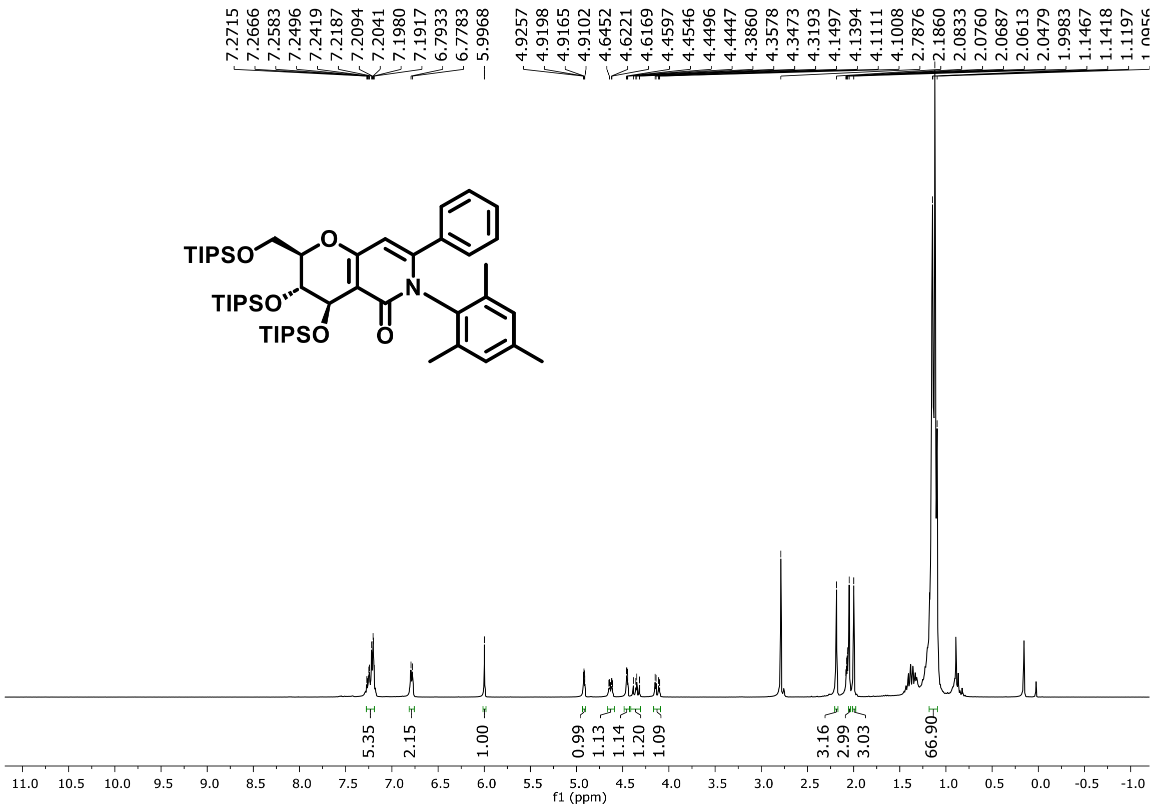

Figure S35. ^{1}H NMR spectra (300 MHz, $(CD_3)_2CO$) of **4c**

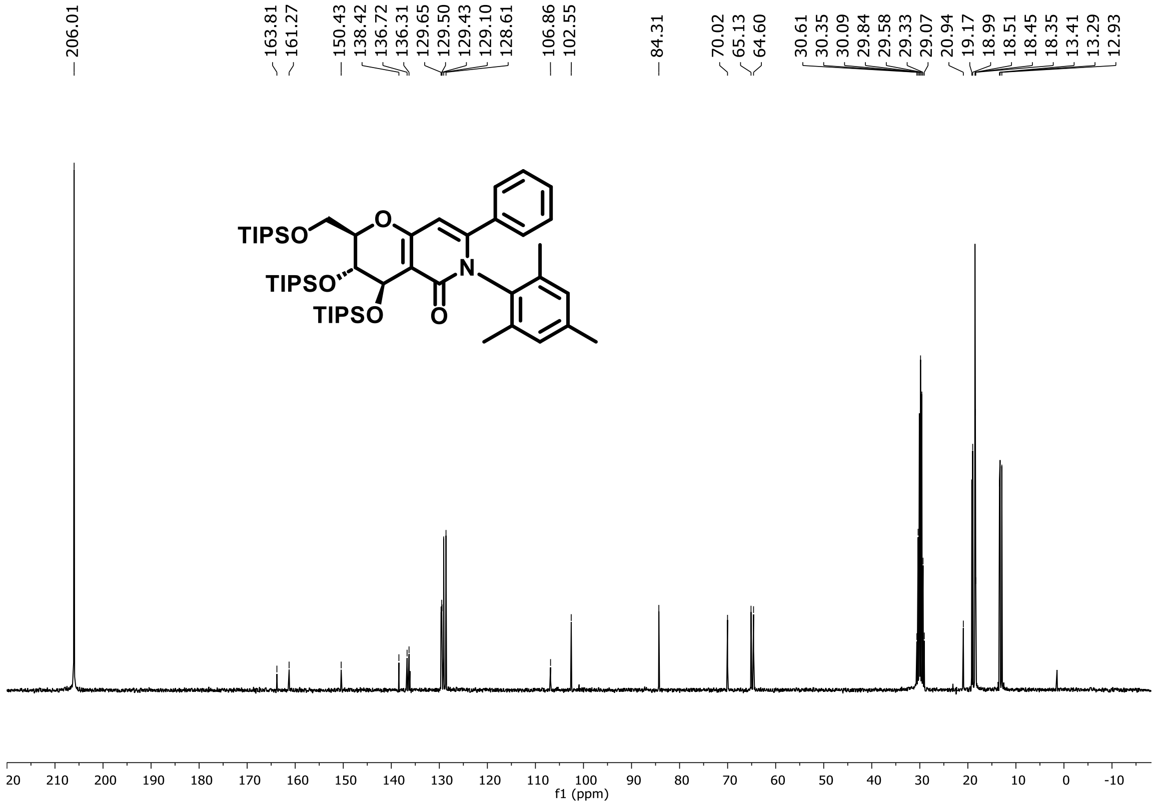

Figure S36. ^{13}C NMR spectra (75 MHz, $(CD_3)_2CO$) of **4c**

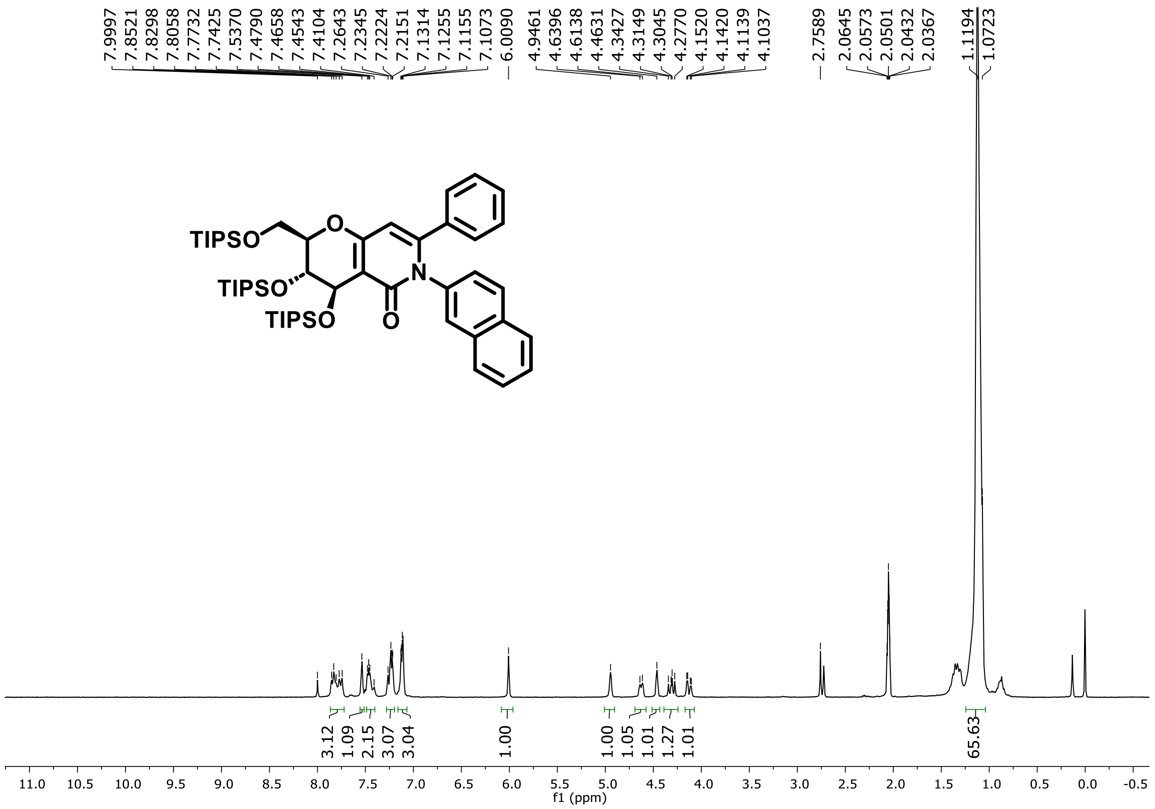

Figure S37. ^{1}H NMR spectra (300 MHz, $(CD_3)_2CO$) of **4d**

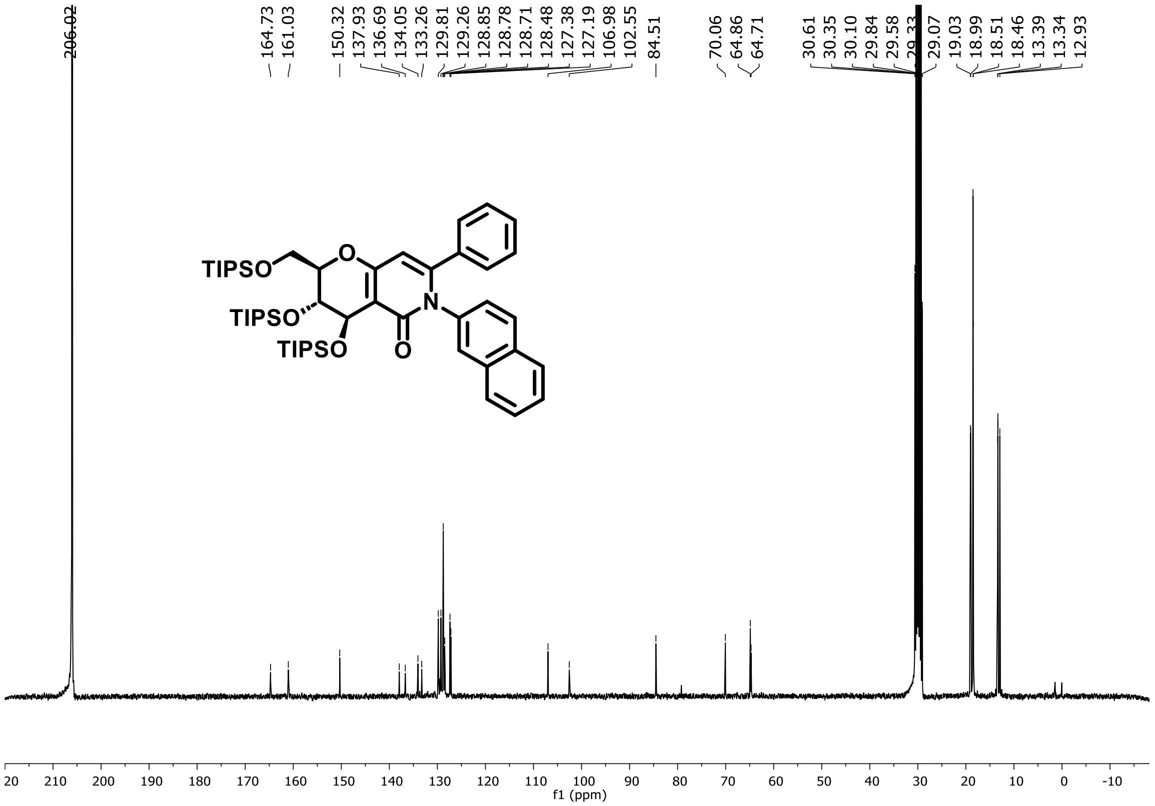

Figure S38. ^{13}C NMR spectra (75 MHz, $(CD_3)_2CO$) of **4d**

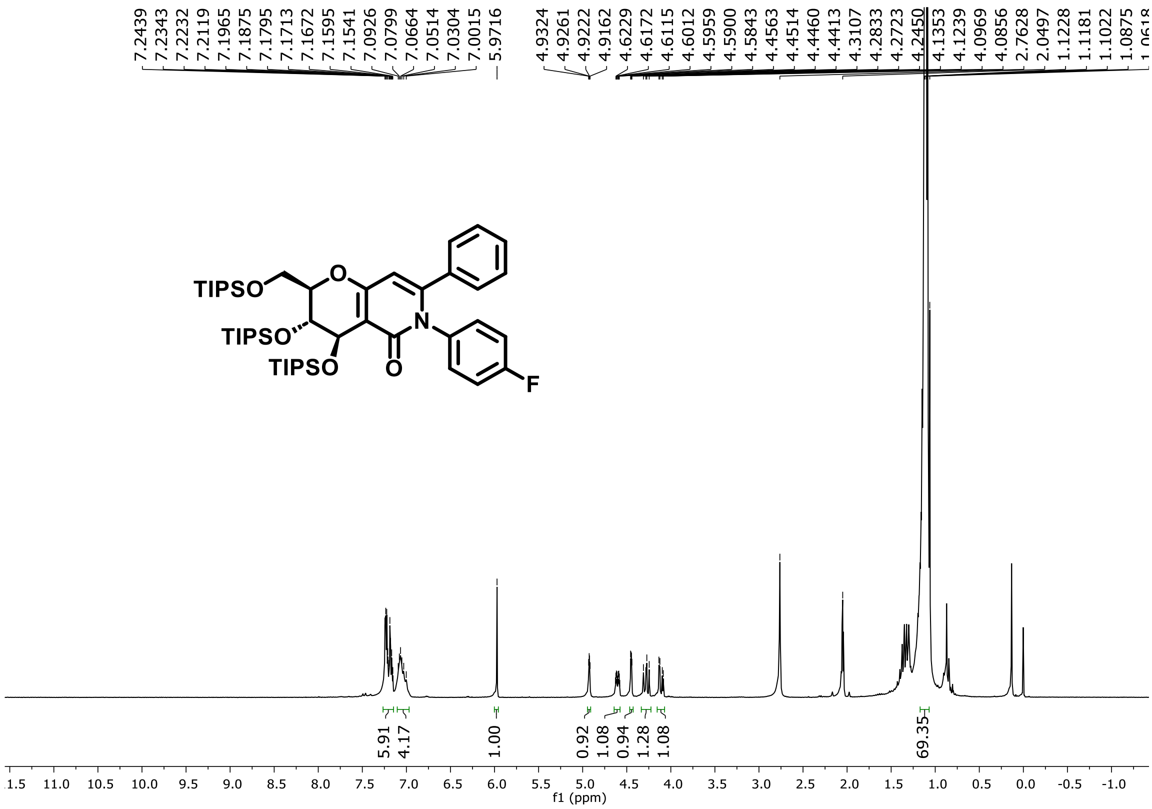

Figure S39. ^{1}H NMR spectra (300 MHz, $(CD_3)_2CO$) of **4e**

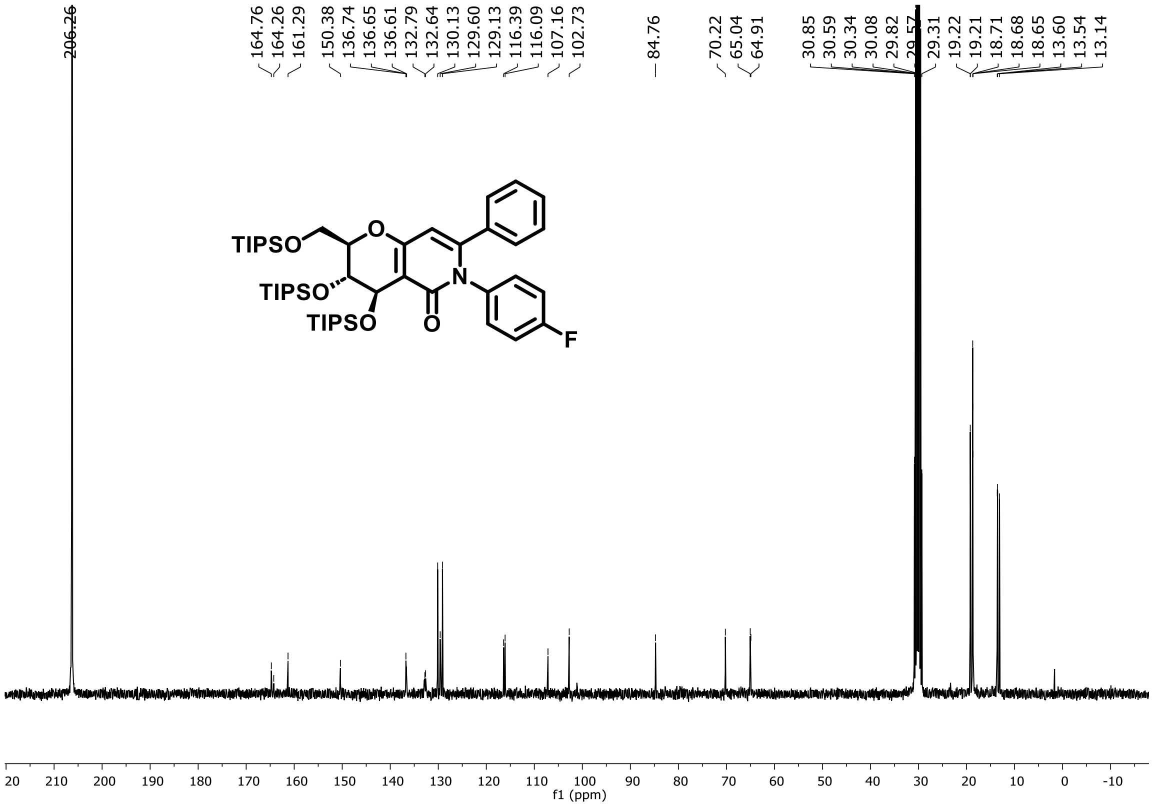

Figure S40. ^{13}C NMR spectra (75 MHz, $(CD_3)_2CO$) of **4e**

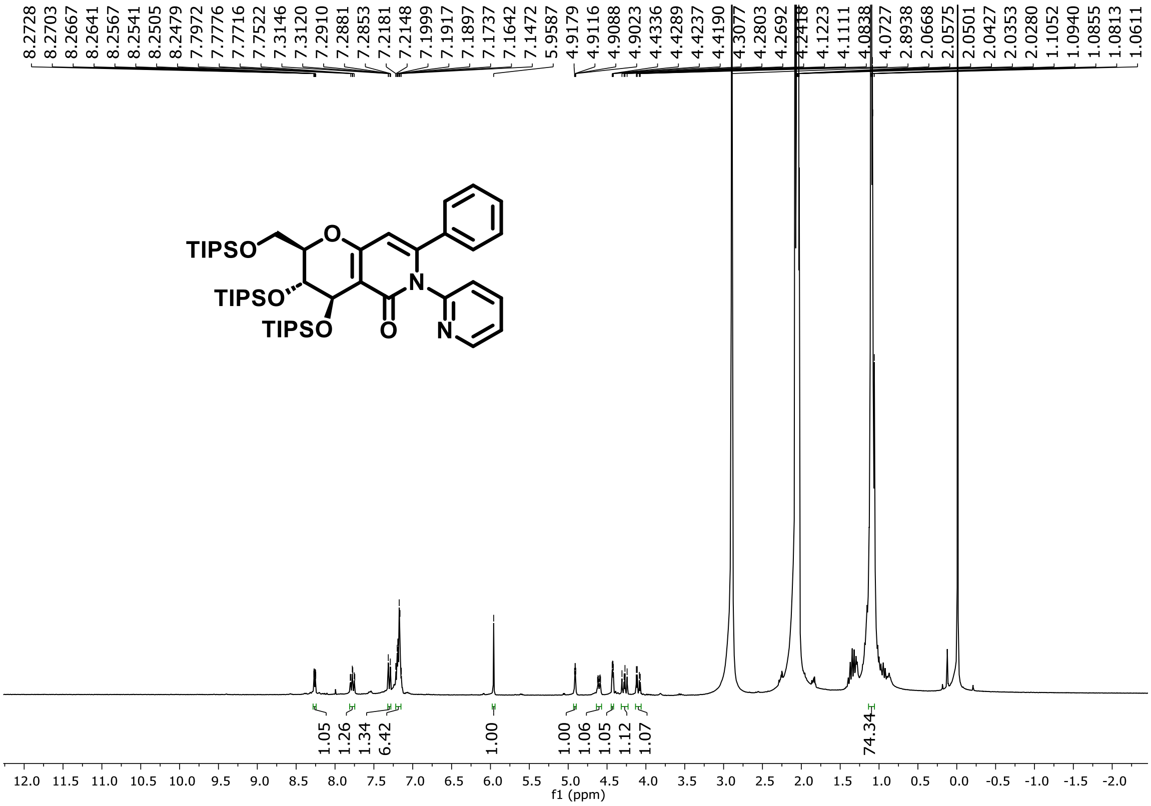

Figure S41. ^{1}H NMR spectra (300 MHz, $(CD_3)_2CO$) of **4g**

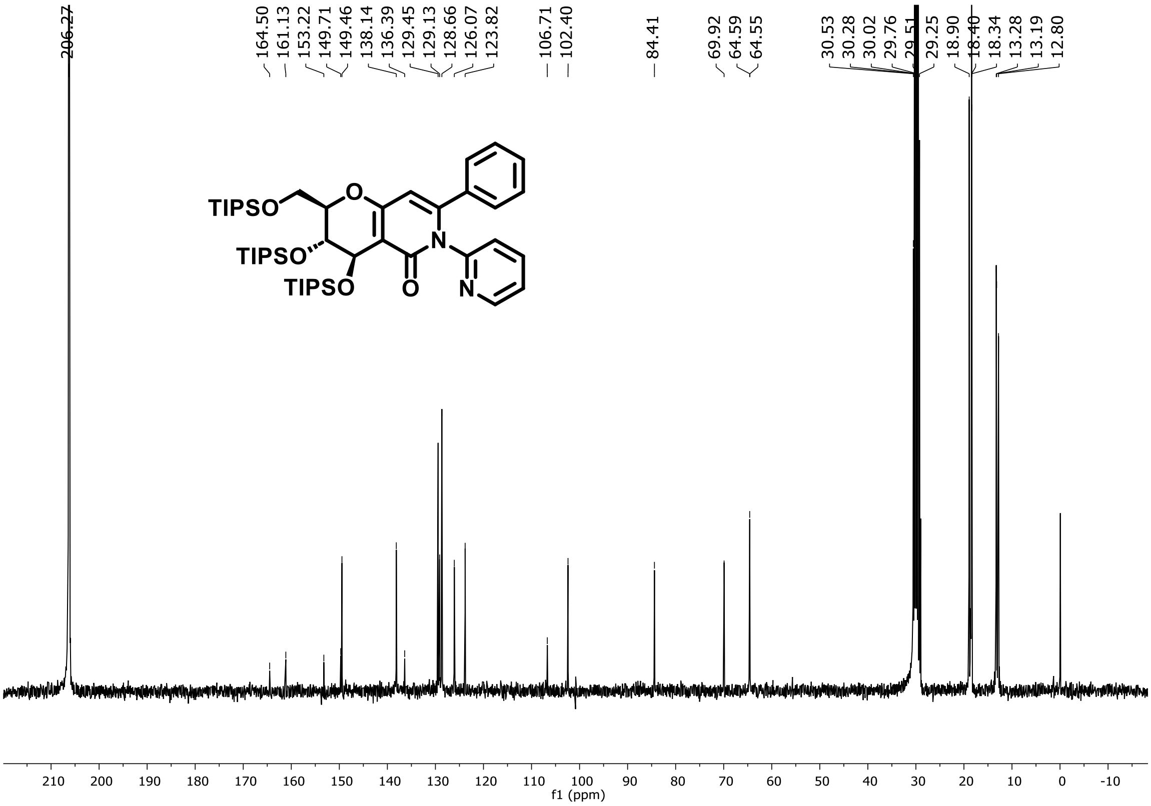

Figure S42. ^{13}C NMR spectra (75 MHz, $(CD_3)_2CO$) of **4g**

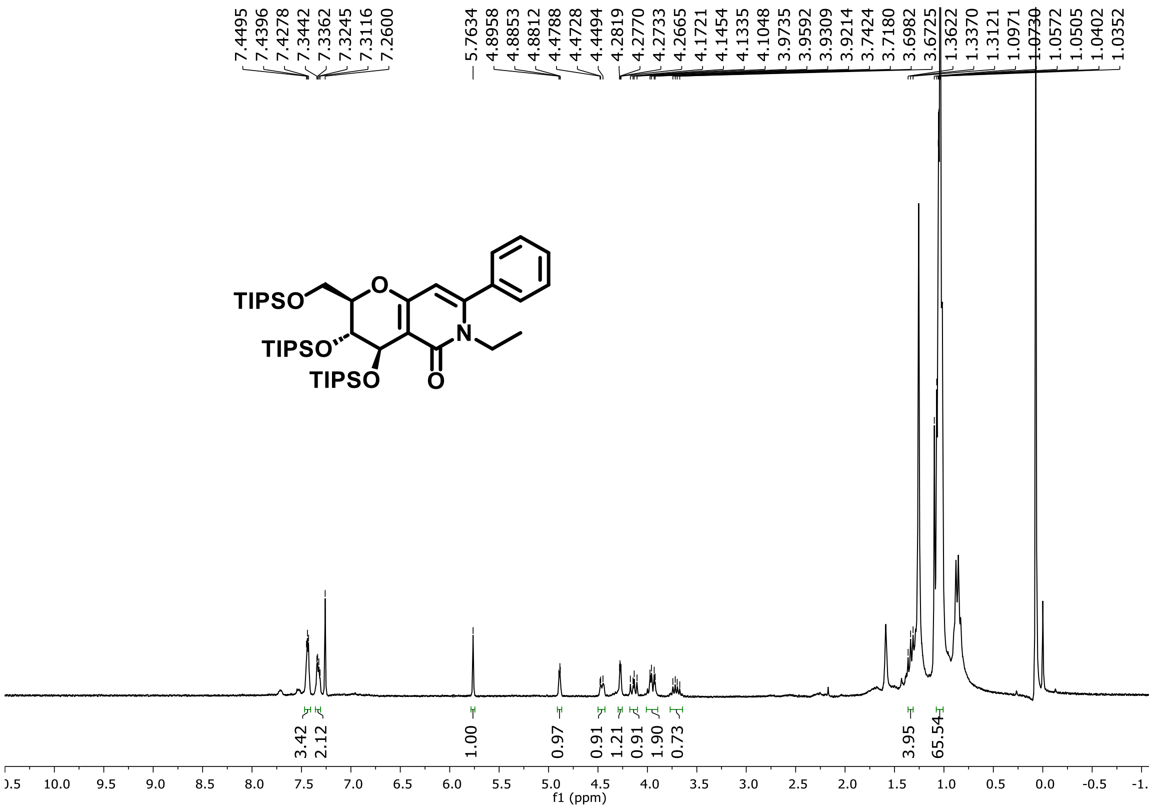

Figure S43. ^{1}H NMR spectra (300 MHz, $CDCl_3$) of **4h**

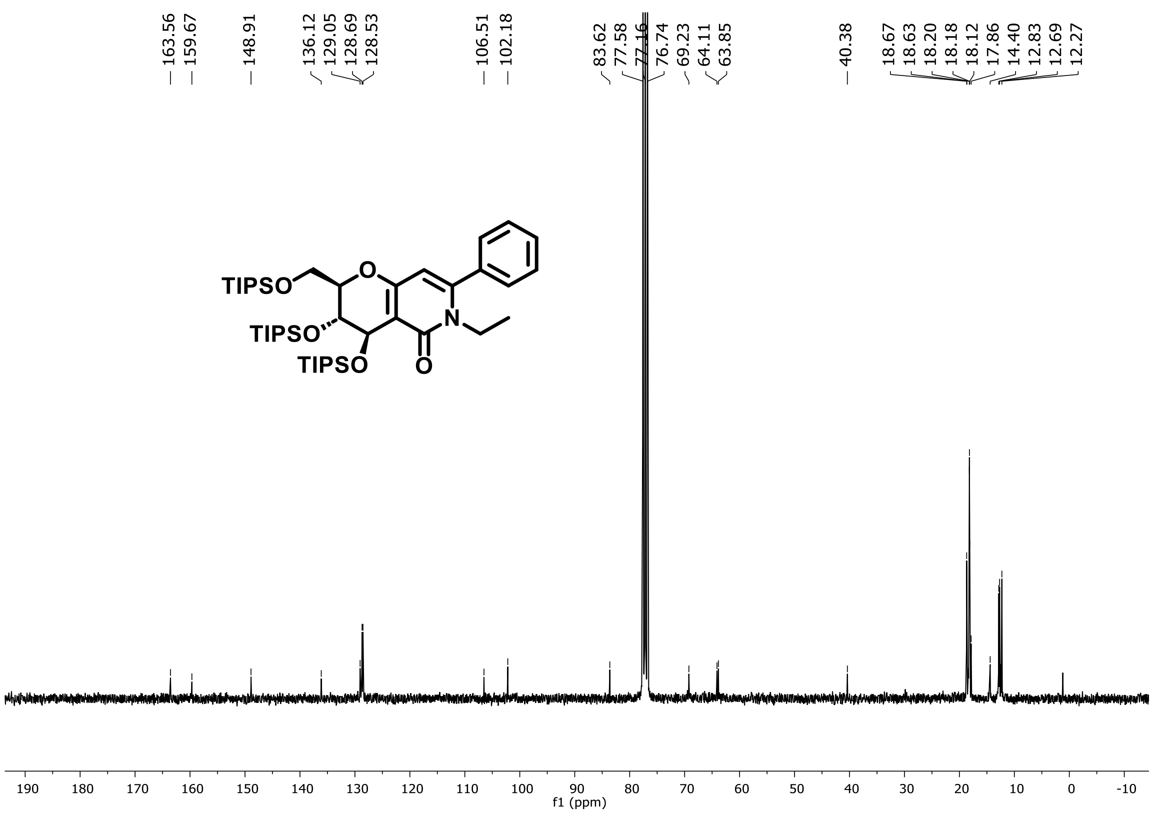

Figure S44. ^{13}C NMR spectra (75 MHz, $CDCl_3$) of **4h**

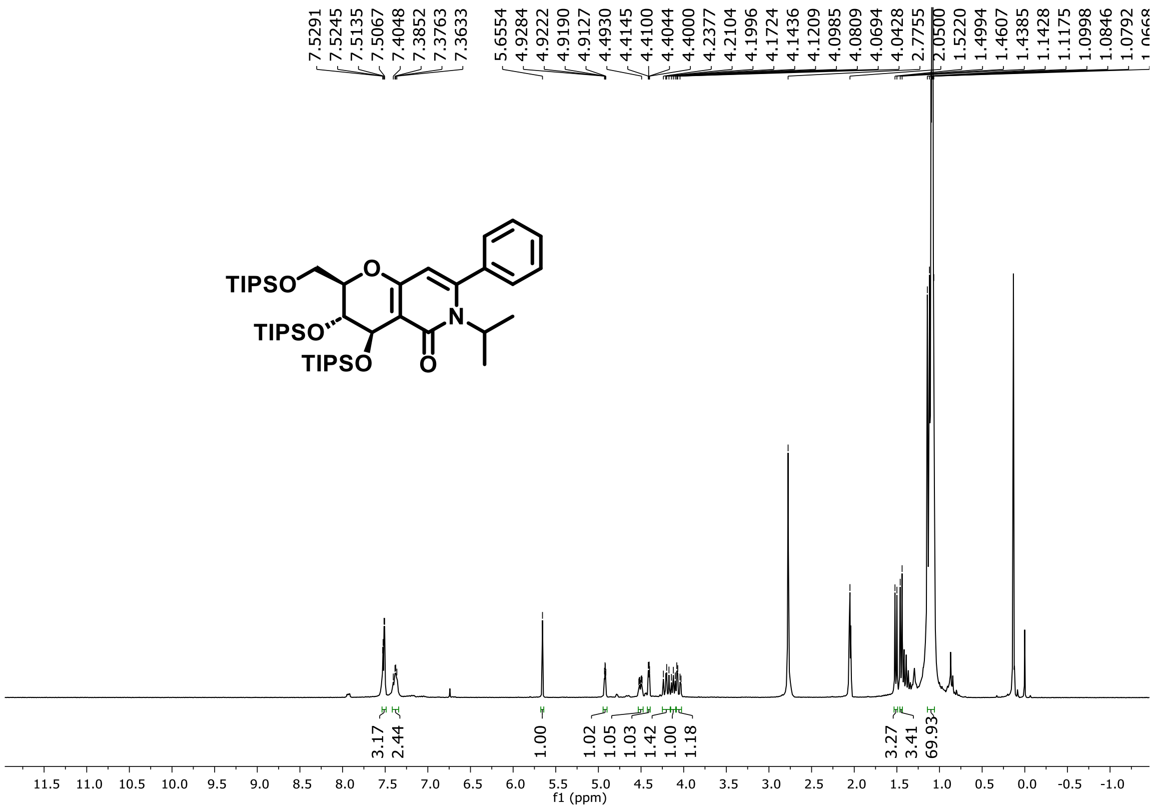

Figure S45. ^{1}H NMR spectra (300 MHz, $(CD_3)_2CO$) of **4i**

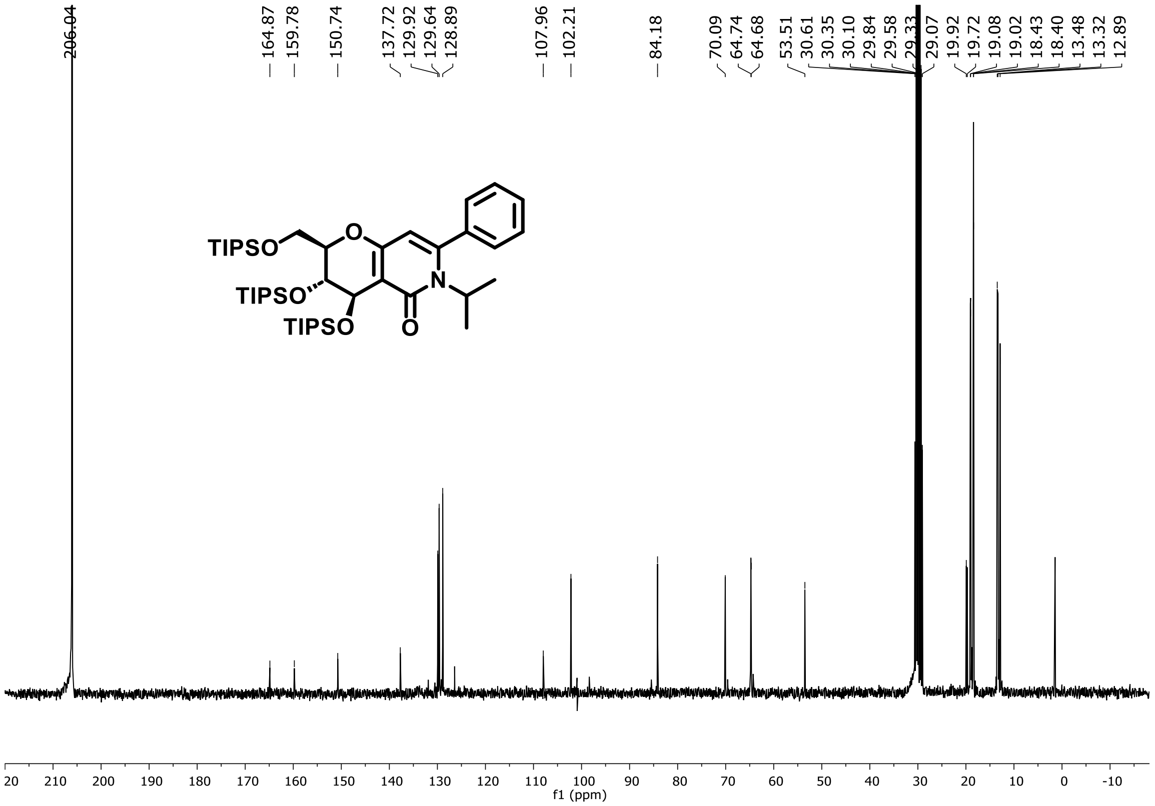

Figure S46. ^{13}C NMR spectra (75 MHz, $(CD_3)_2CO$) of **4i**

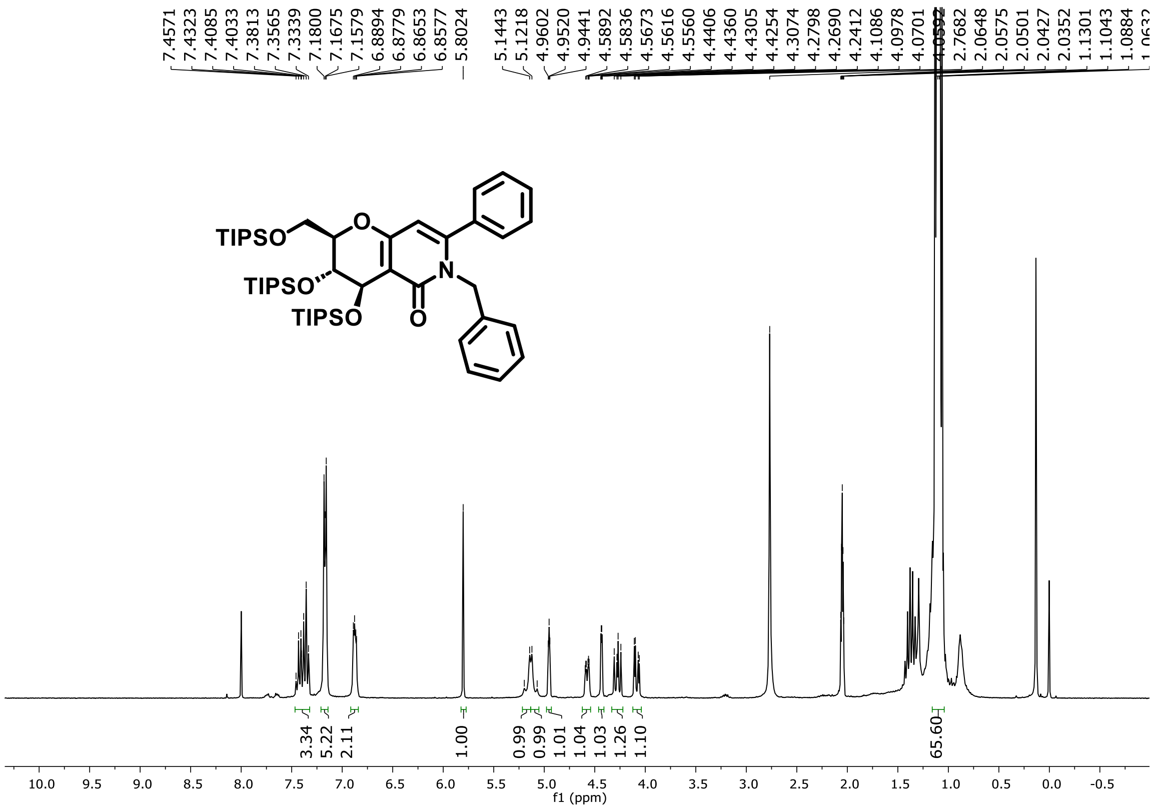

Figure S47. ^{1}H NMR spectra (300 MHz, $(CD_3)_2CO$) of **4j**

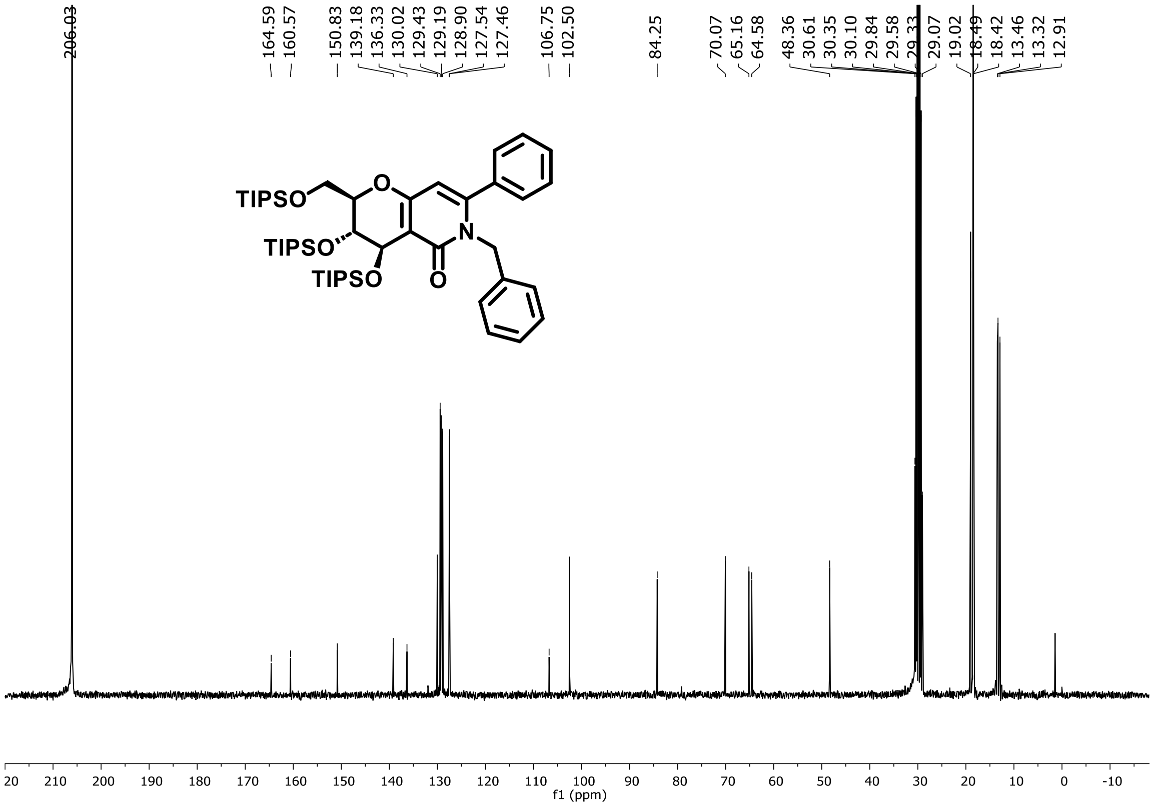

Figure S48. ^{13}C NMR spectra (75 MHz, $(CD_3)_2CO$) of **4j**

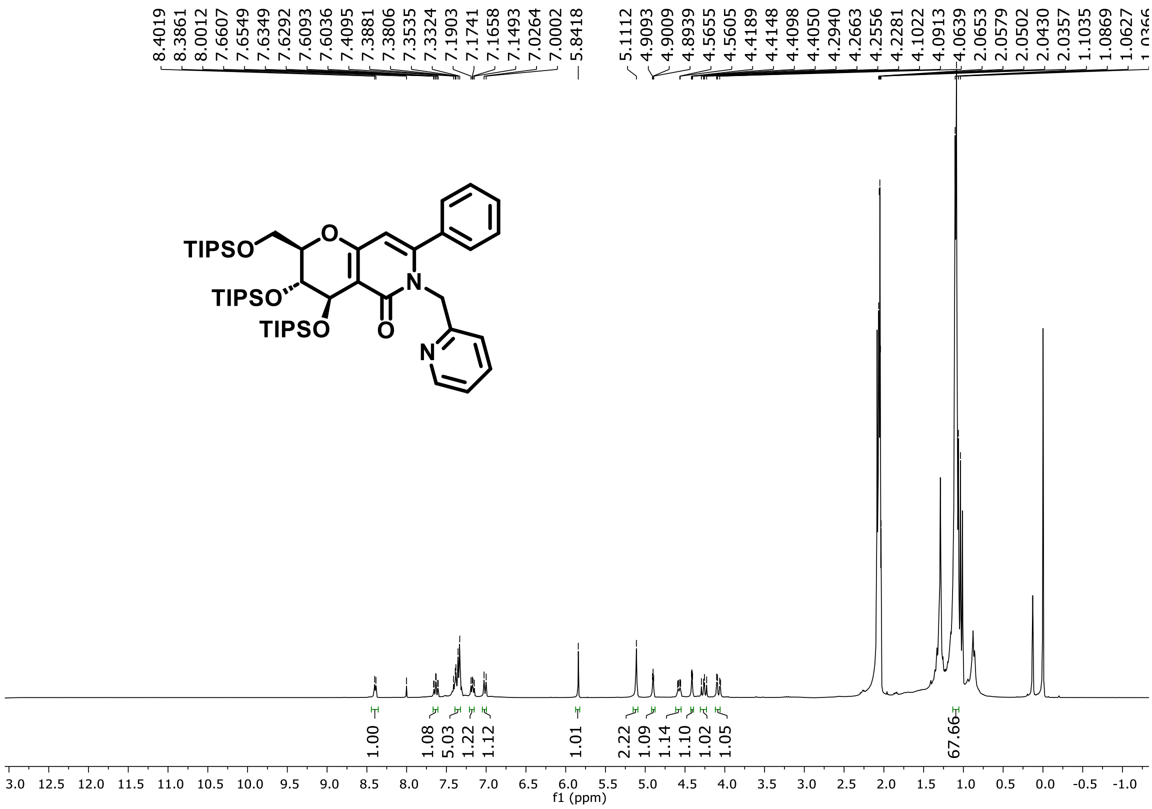

Figure S49. ^{1}H NMR spectra (300 MHz, $(CD_3)_2CO$) of **4k**

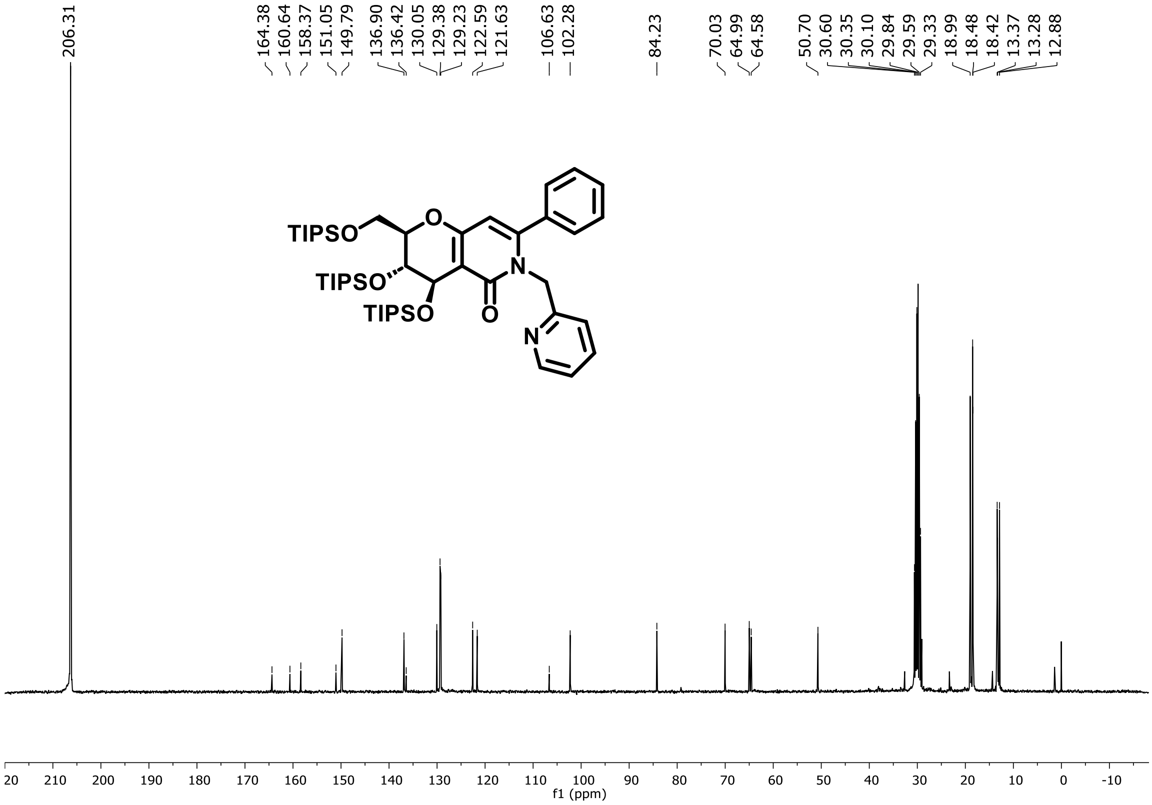

Figure S50. ^{13}C NMR spectra (75 MHz, $(CD_3)_2CO$) of **4k**

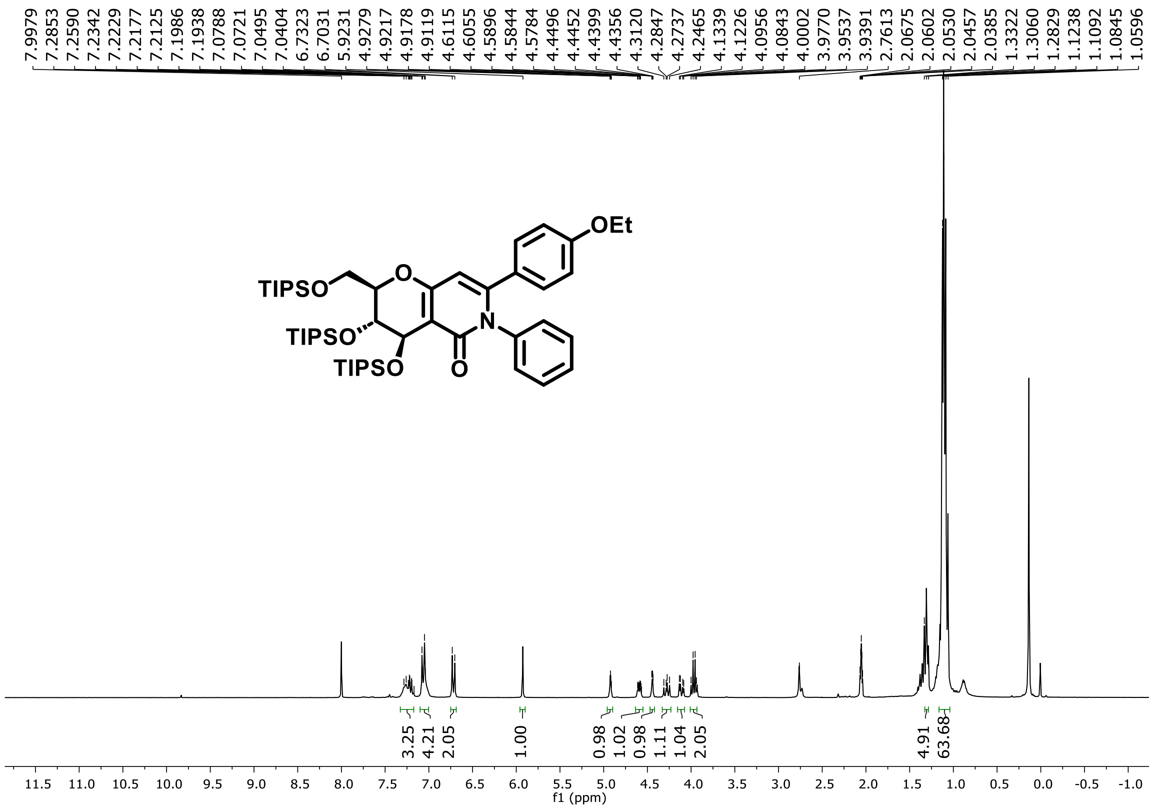

Figure S51. ^{1}H NMR spectra (300 MHz, $(CD_3)_2CO$) of **5a**

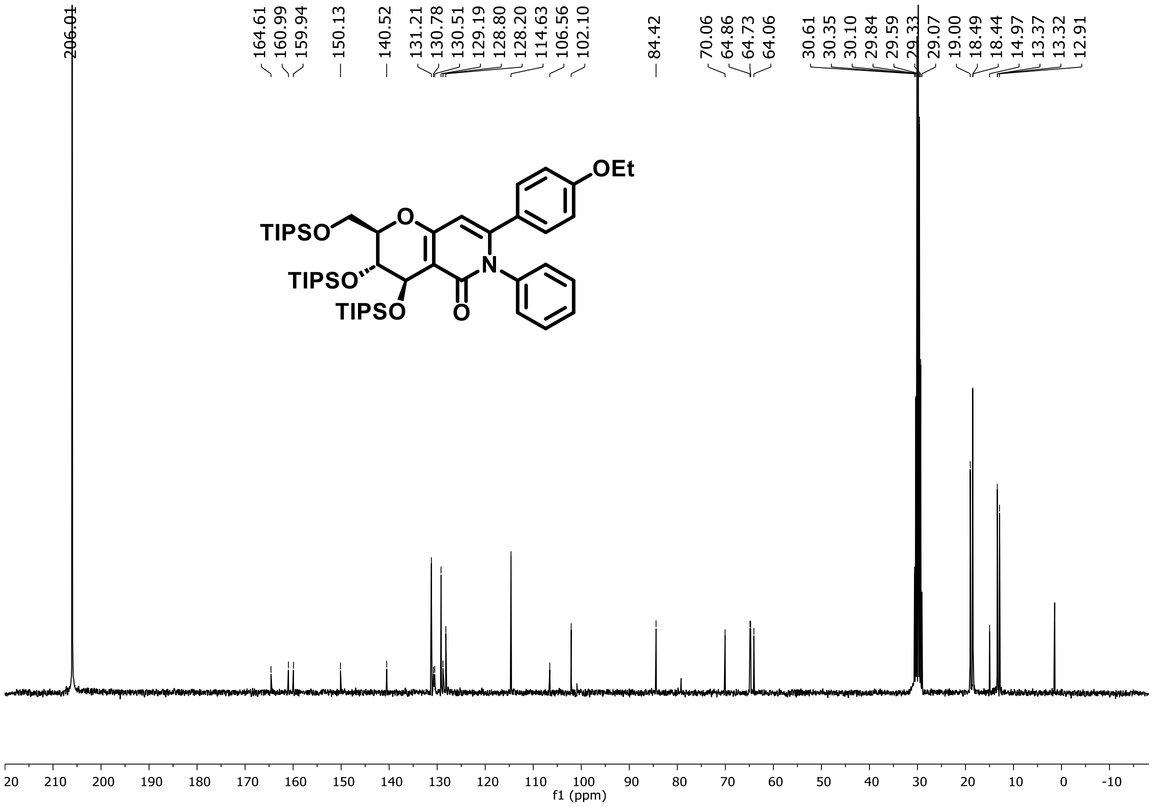

Figure S52. ^{13}C NMR spectra (75 MHz, $(CD_3)_2CO$) of **5a**

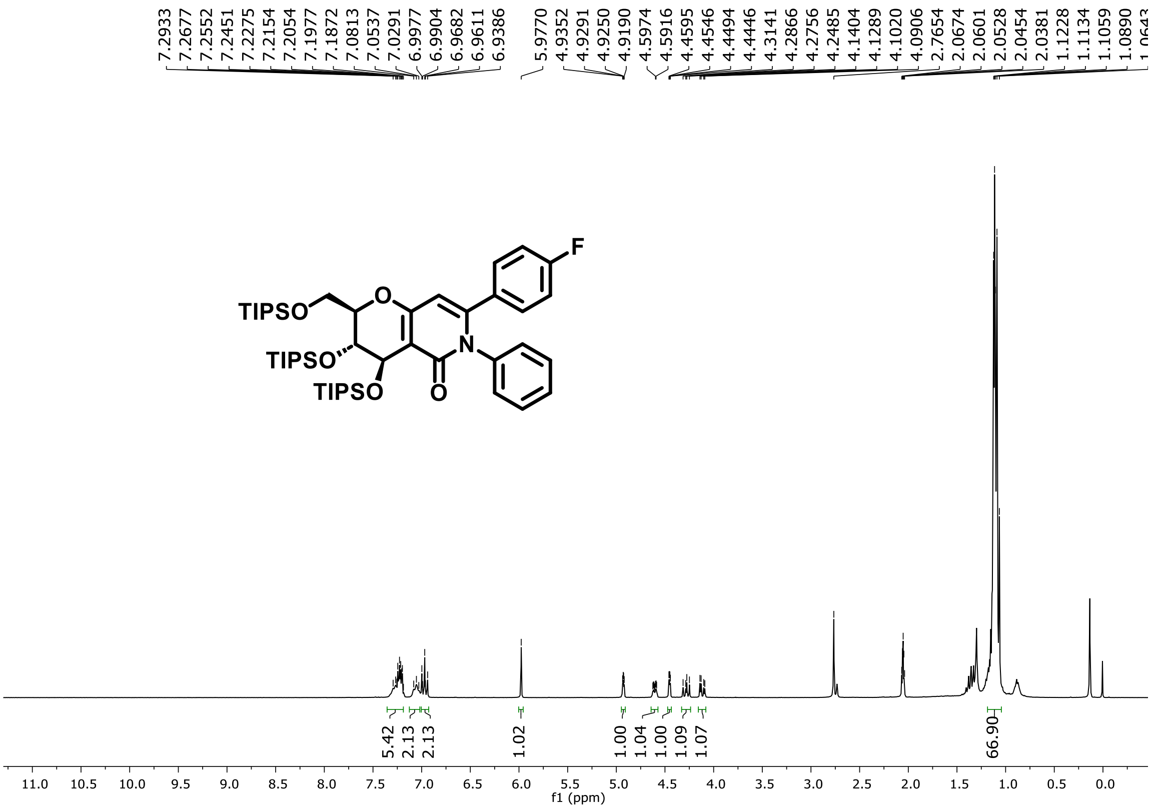

Figure S53. ^{1}H NMR spectra (300 MHz, $(CD_3)_2CO$) of **5b**

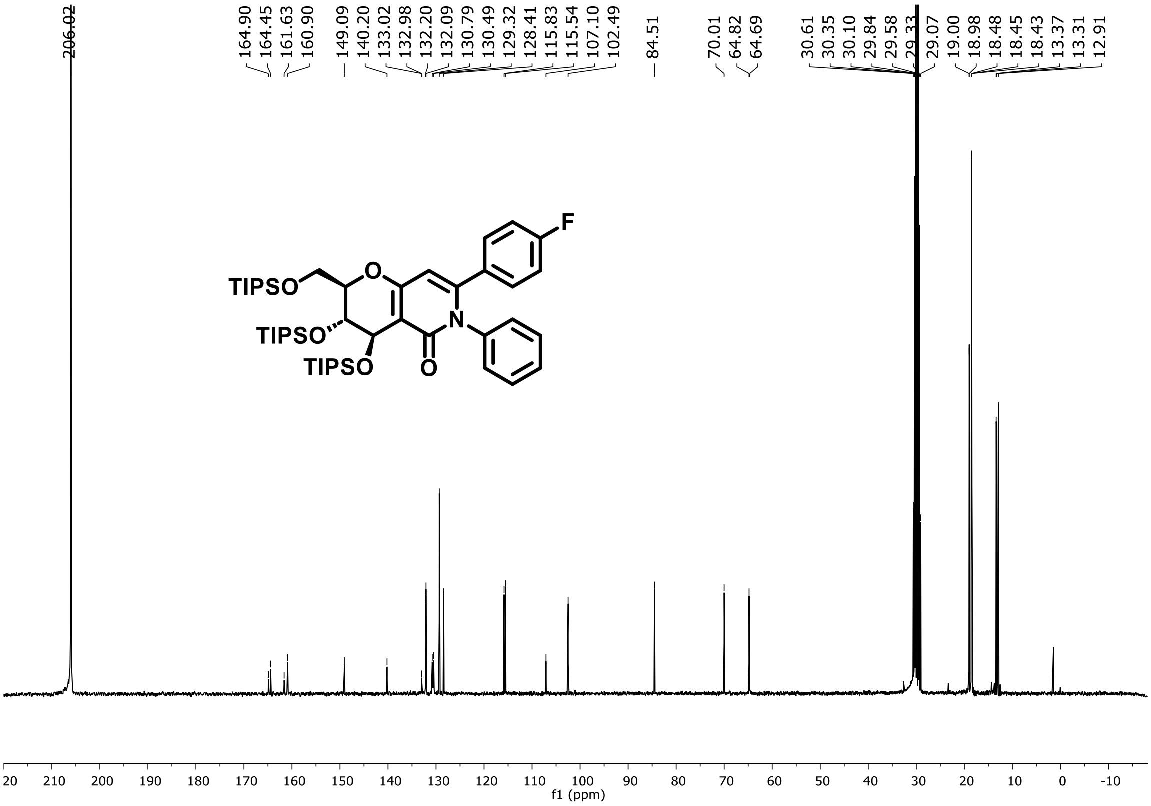

Figure S54. ^{13}C NMR spectra (75 MHz, $(CD_3)_2CO$) of **5b**

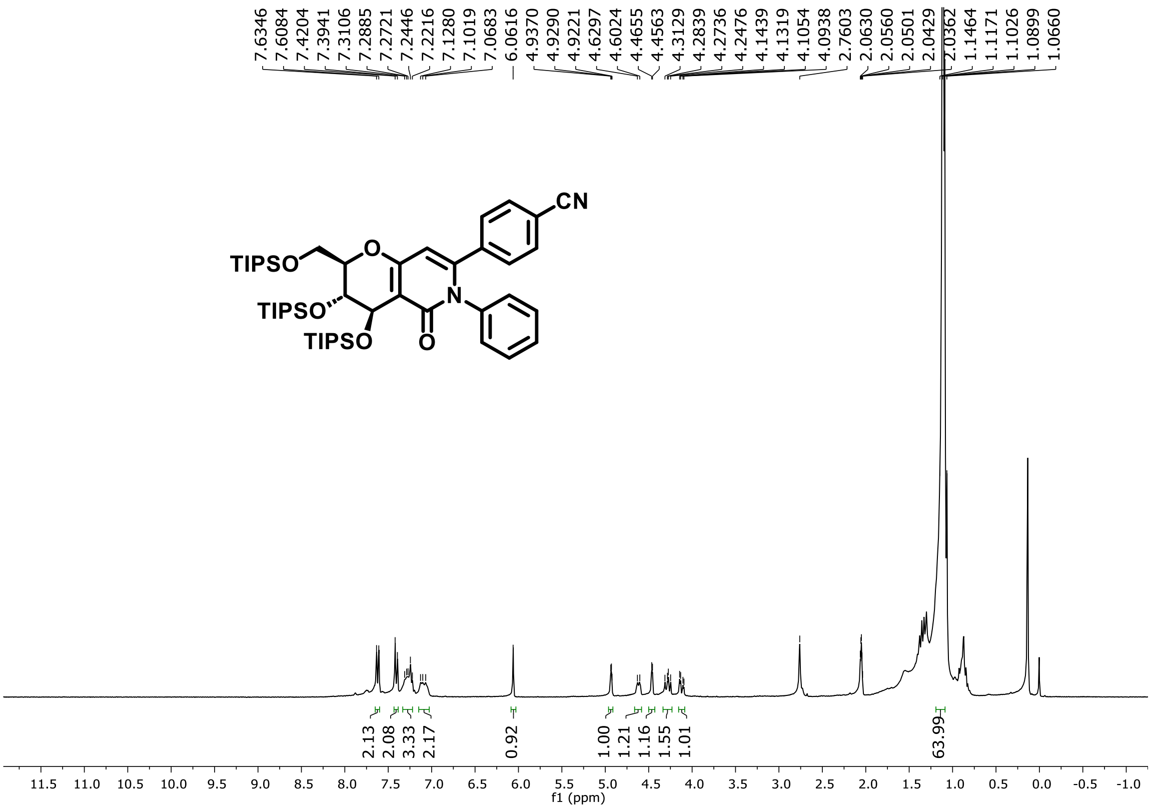

Figure S55. ^{1}H NMR spectra (300 MHz, $(CD_3)_2CO$) of **5c**

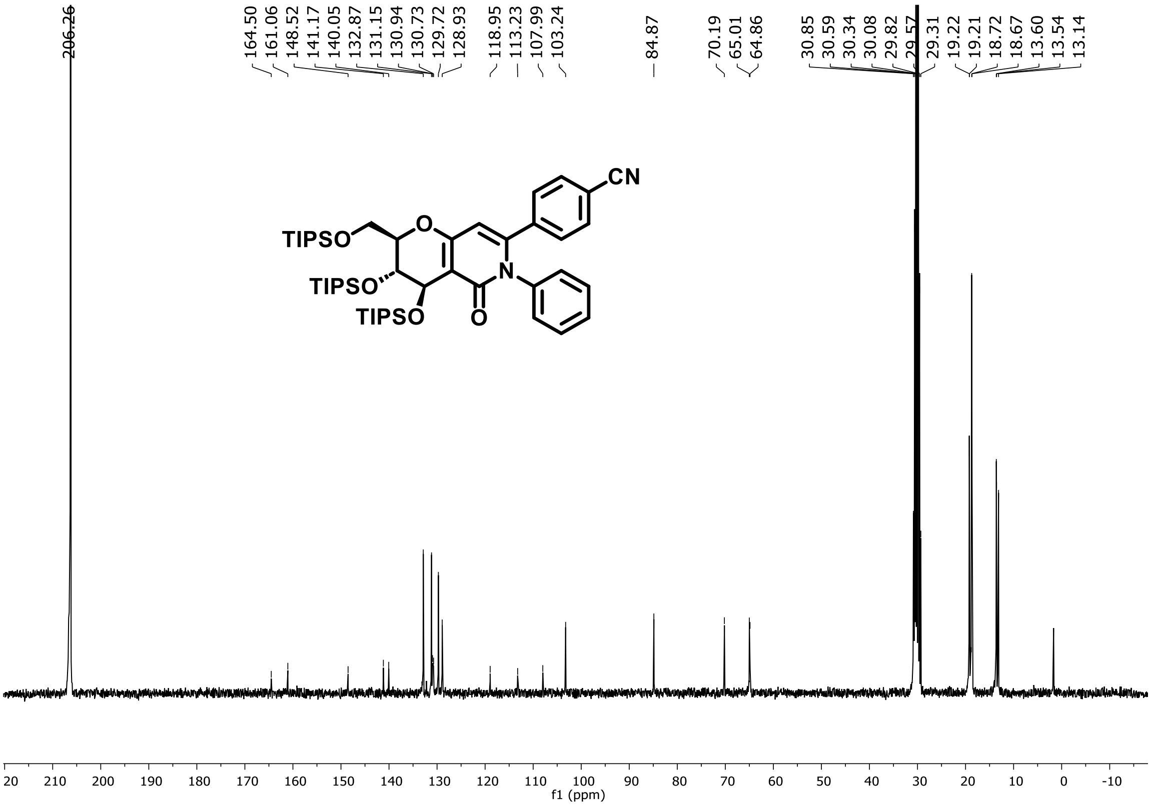

Figure S56. ^{13}C NMR spectra (75 MHz, $(CD_3)_2CO$) of **5c**

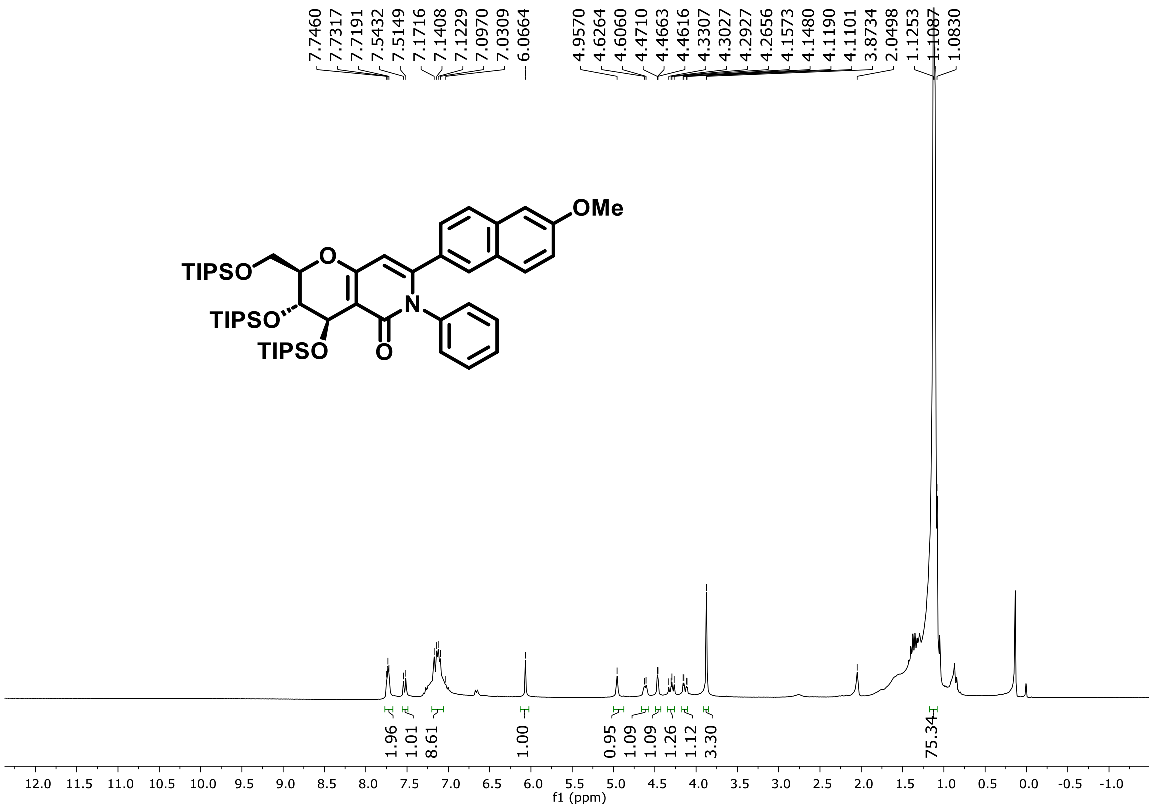

Figure S57. ^{1}H NMR spectra (300 MHz, $(CD_3)_2CO$) of **5d**

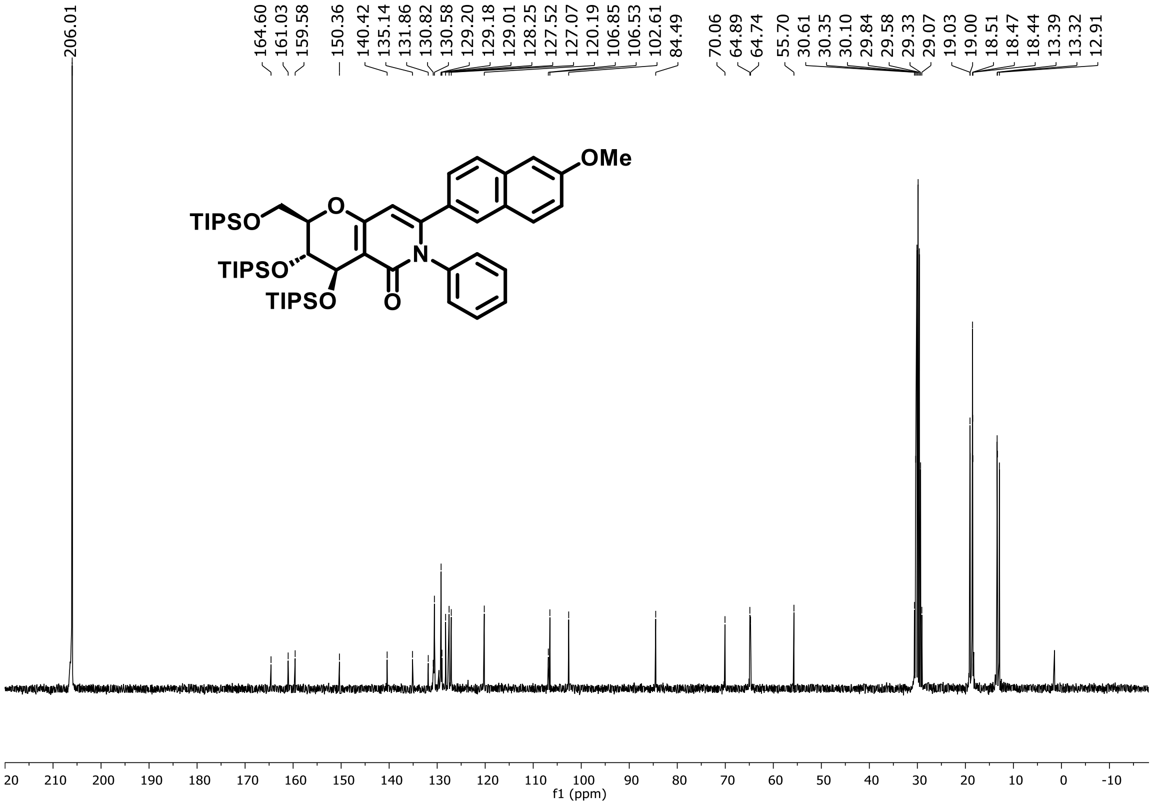

Figure S58. ^{13}C NMR spectra (75 MHz, $(CD_3)_2CO$) of **5d**

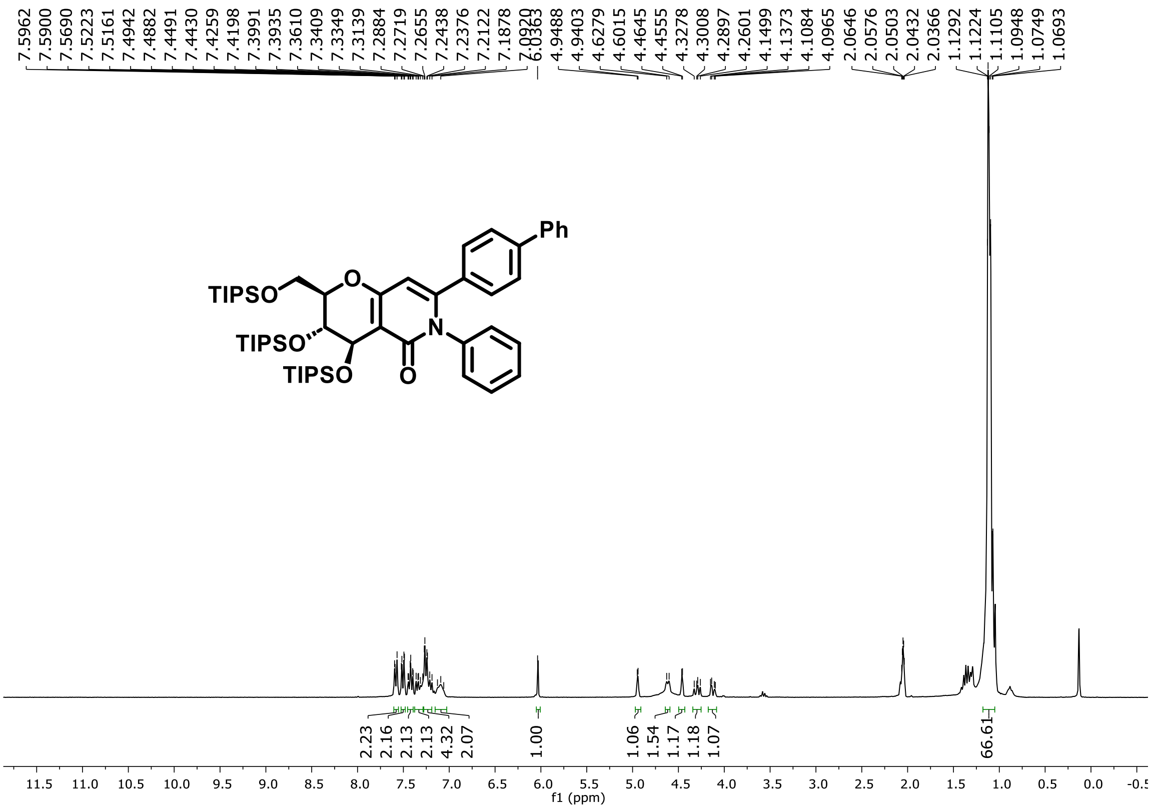

Figure S59. ^{1}H NMR spectra (300 MHz, $(CD_3)_2CO$) of **5e**

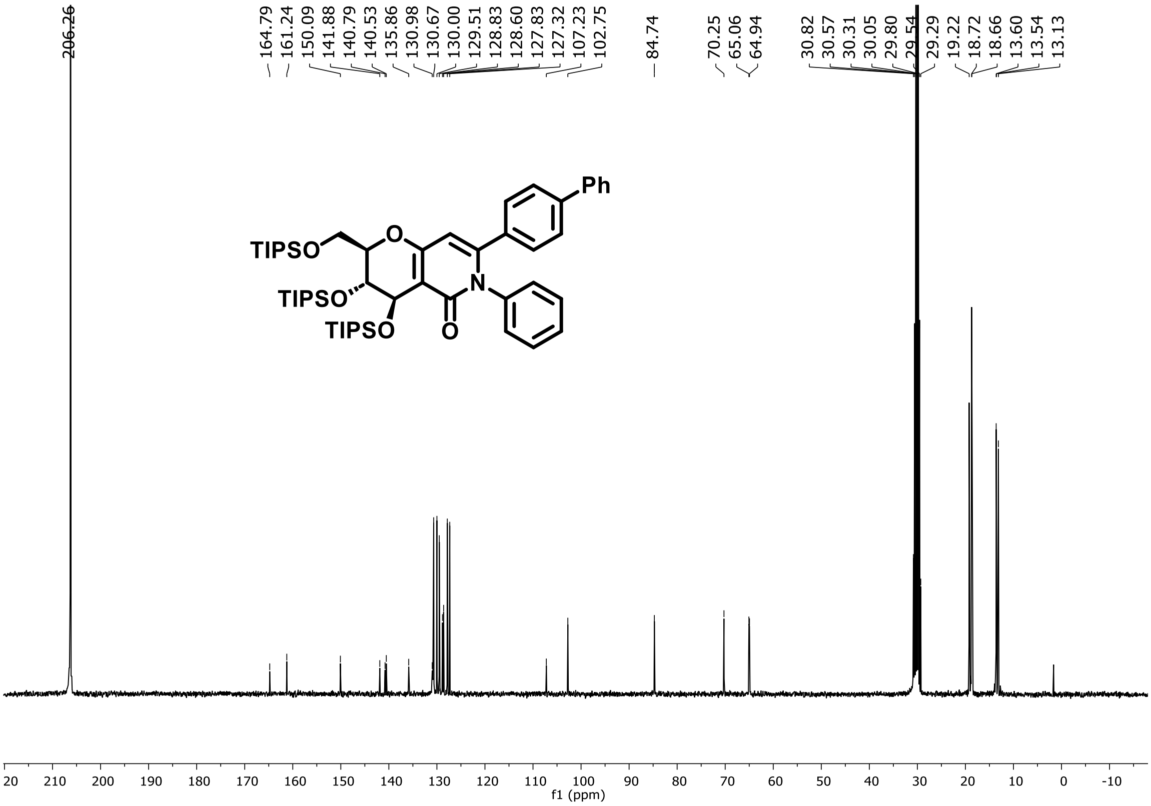

Figure S60. ^{13}C NMR spectra (75 MHz, $(CD_3)_2CO$) of **5e**

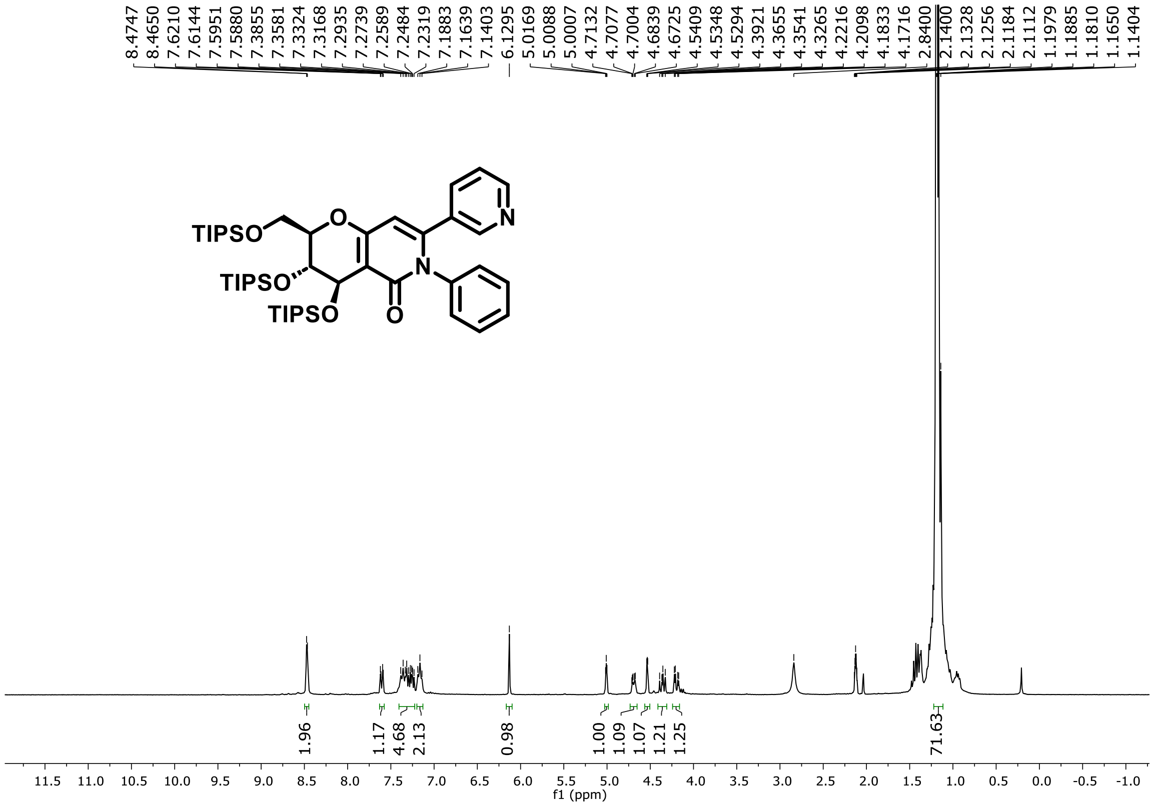

Figure S61. ^{1}H NMR spectra (300 MHz, $(CD_3)_2CO$) of **5f**

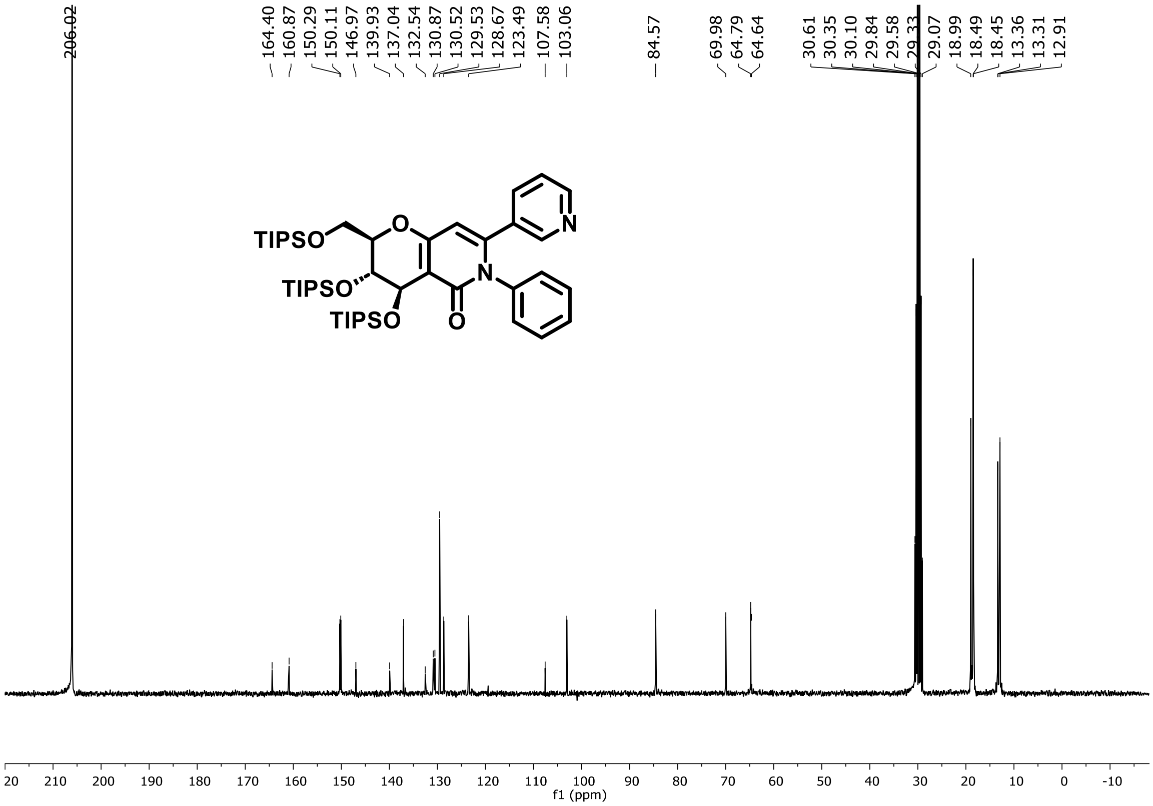

Figure S62. ^{13}C NMR spectra (75 MHz, $(CD_3)_2CO$) of **5f**

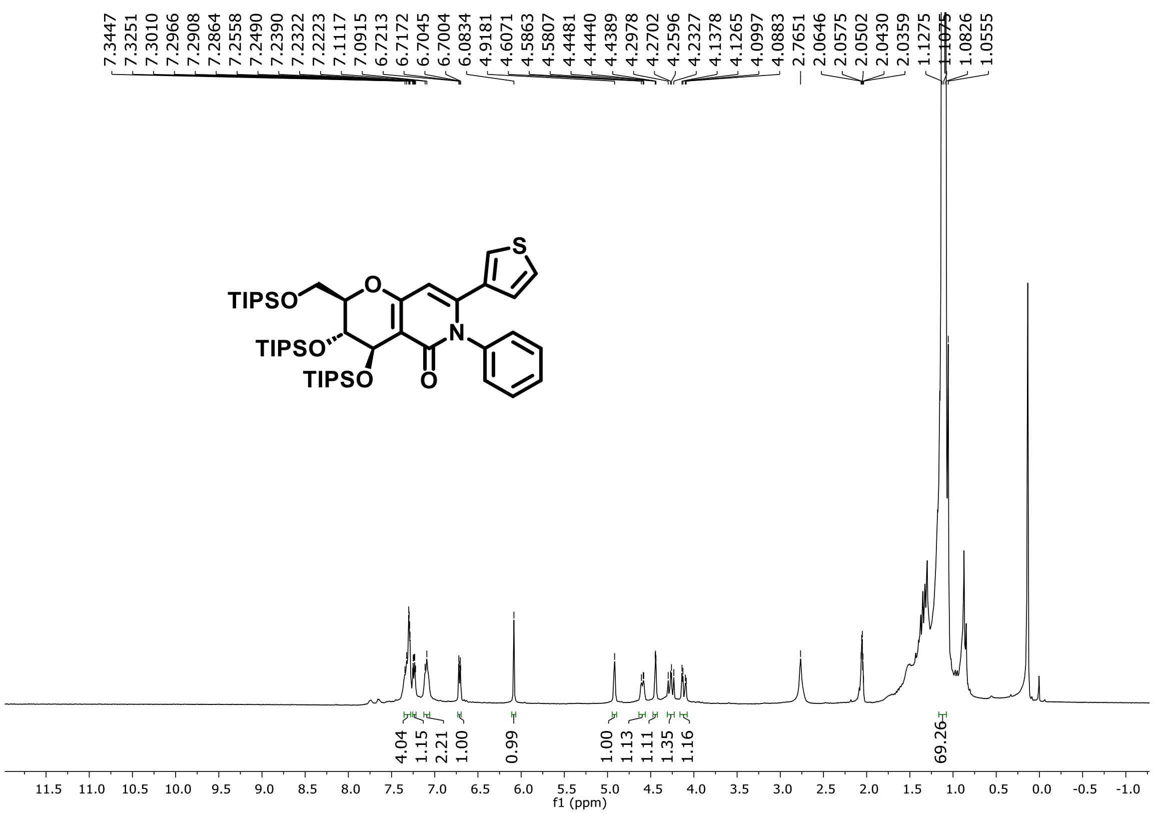

Figure S63. ^{1}H NMR spectra (300 MHz, $(CD_3)_2CO$) of **5g**

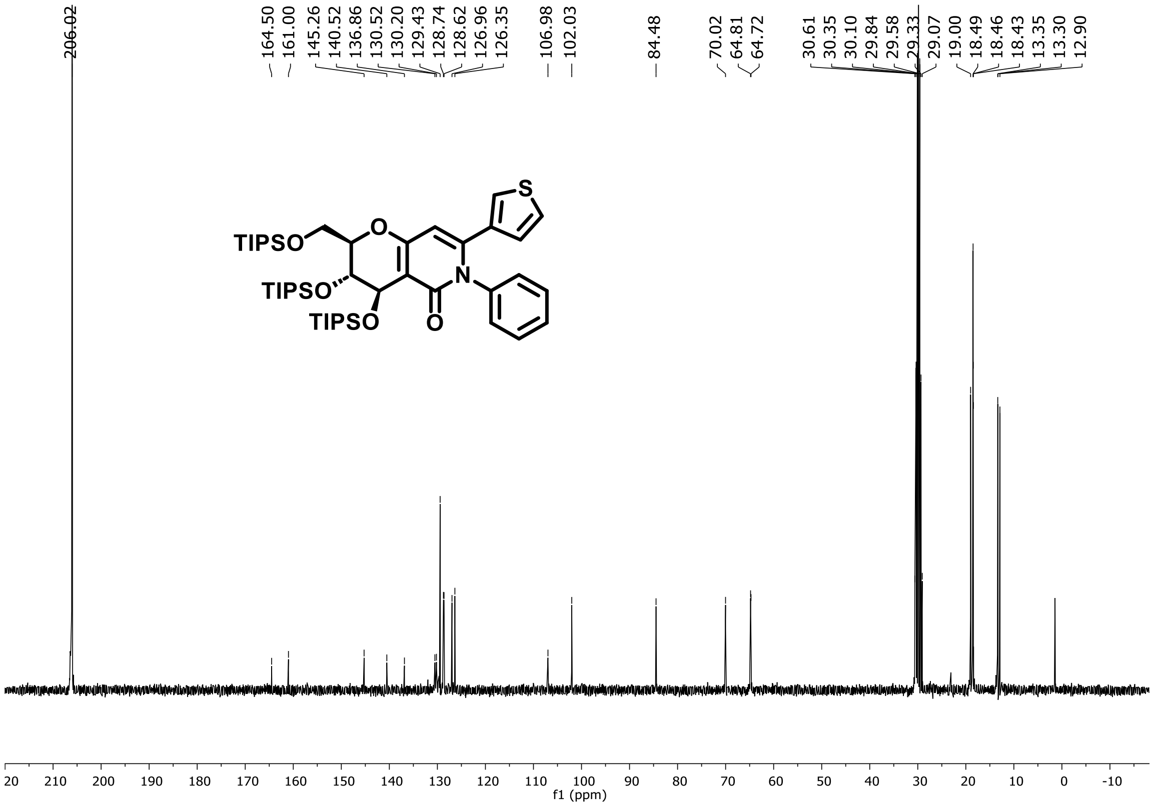

Figure S64. ^{13}C NMR spectra (75 MHz, $(CD_3)_2CO$) of **5g**

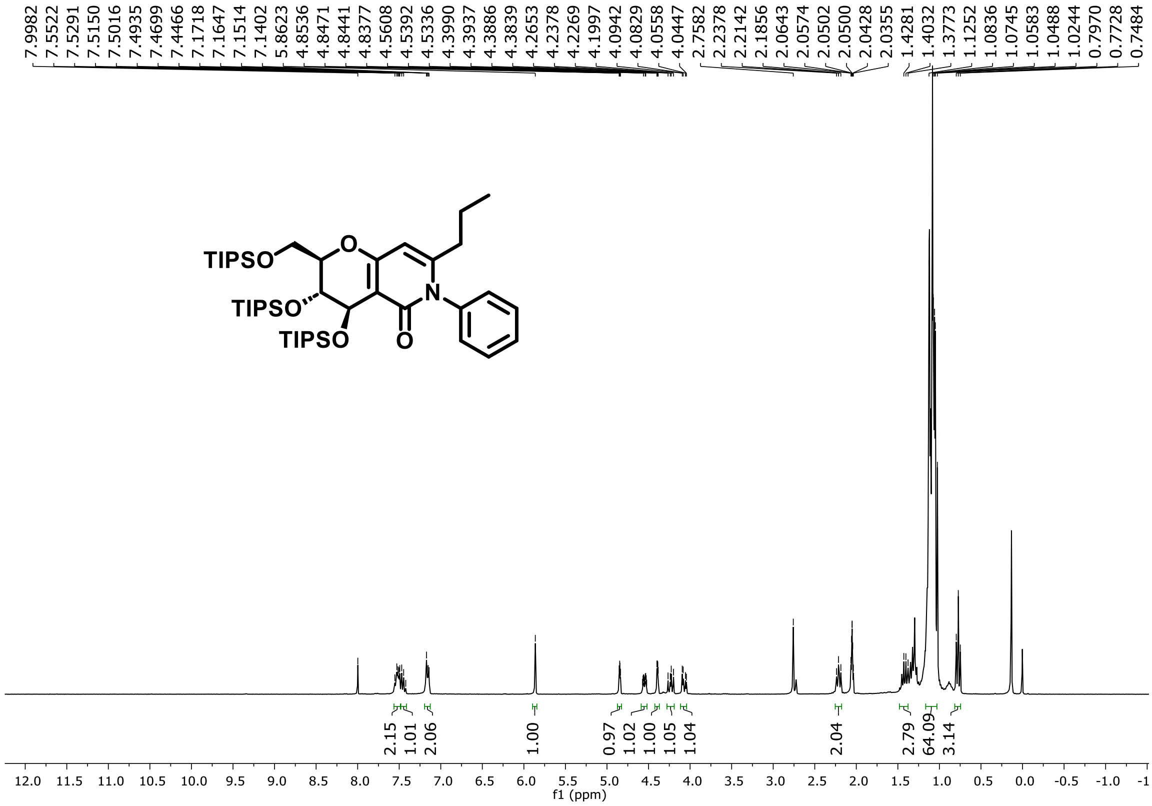

Figure S65. ^{1}H NMR spectra (300 MHz, $(CD_3)_2CO$) of **5h**

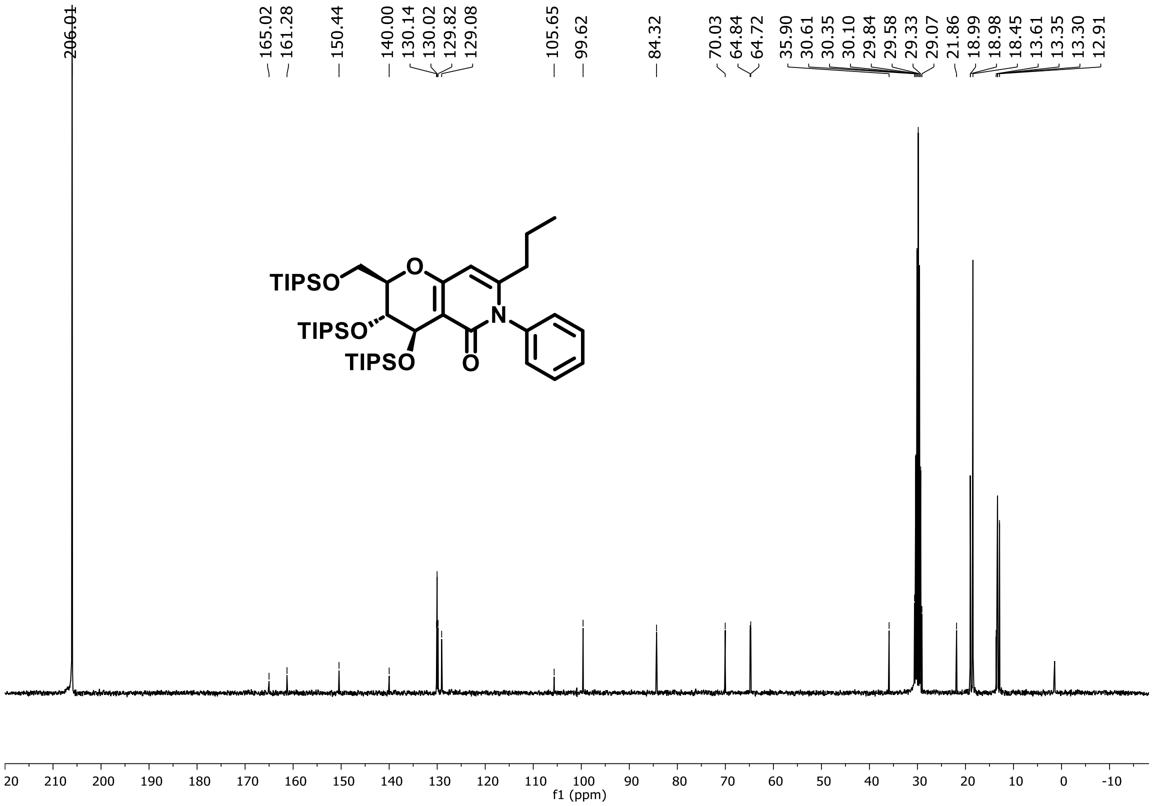

Figure S66. ^{13}C NMR spectra (75 MHz, $(CD_3)_2CO$) of **5h**

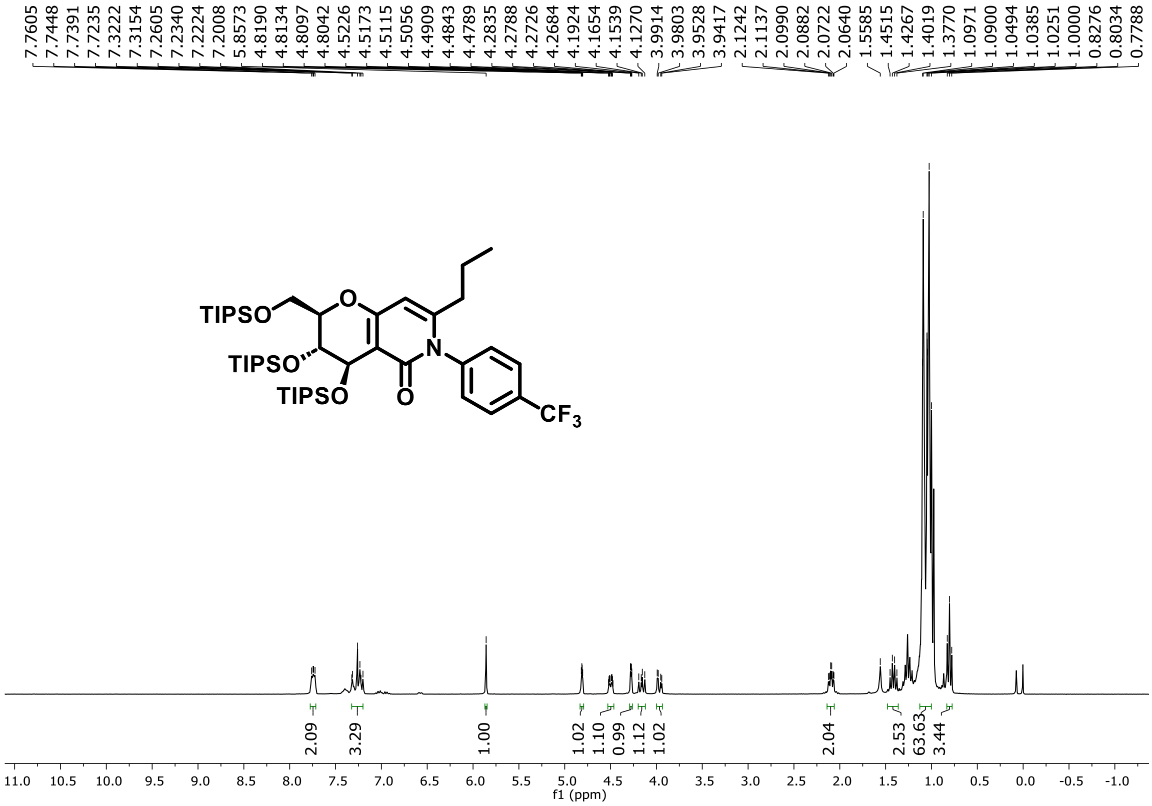

Figure S67. ^{1}H NMR spectra (300 MHz, $CDCl_3$) of **5i**

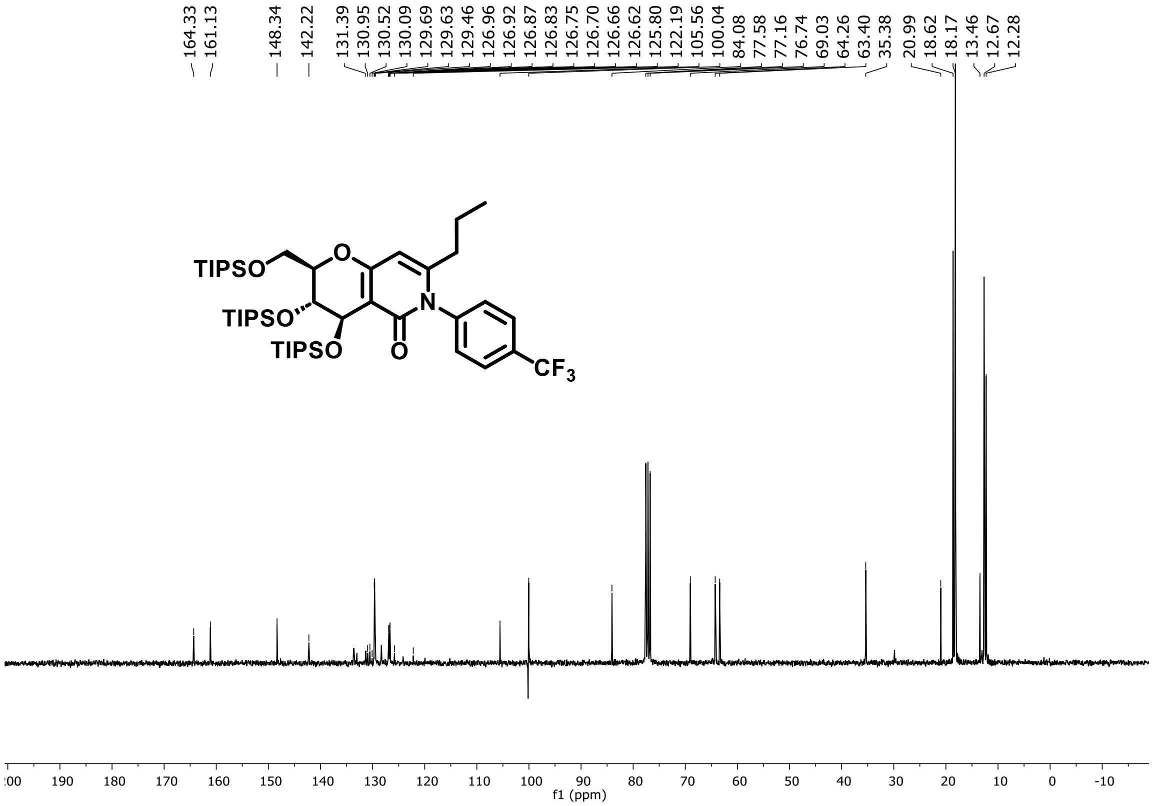

Figure S68. ^{13}C NMR spectra (75 MHz, $CDCl_3$) of **5i**

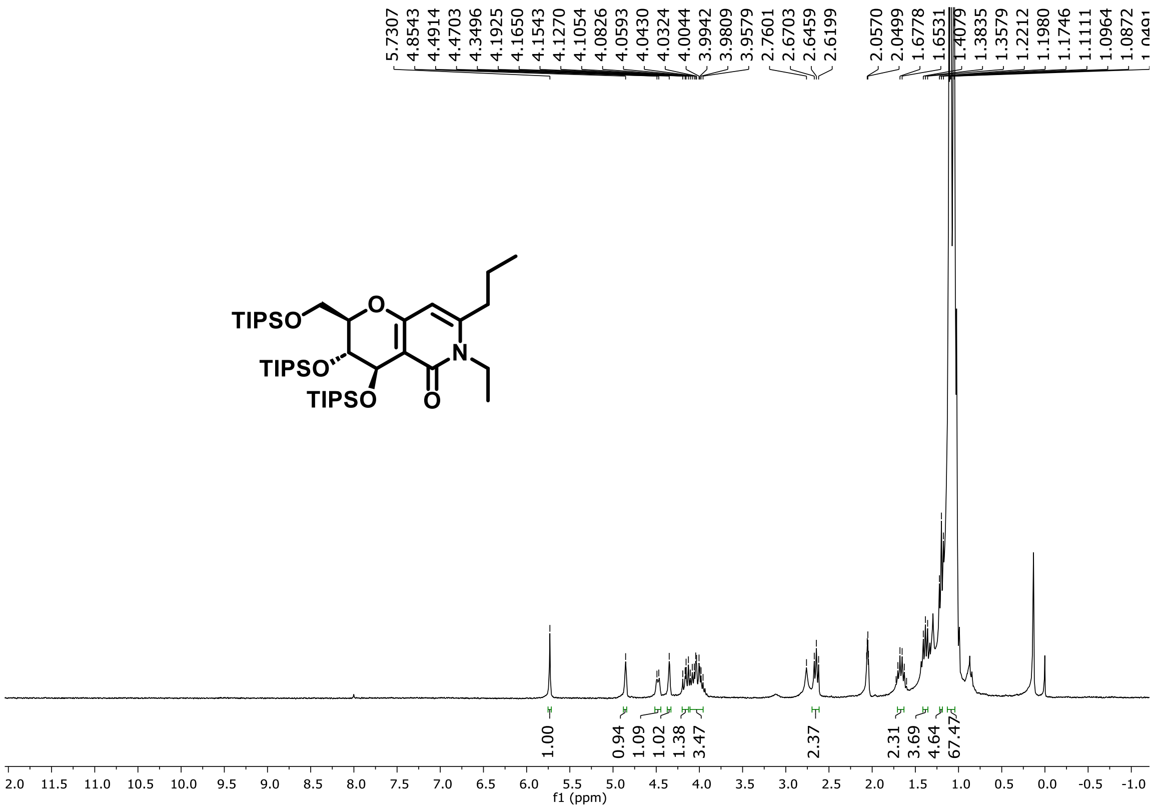

Figure S69. ^{1}H NMR spectra (300 MHz, $(CD_3)_2CO$) of **5j**

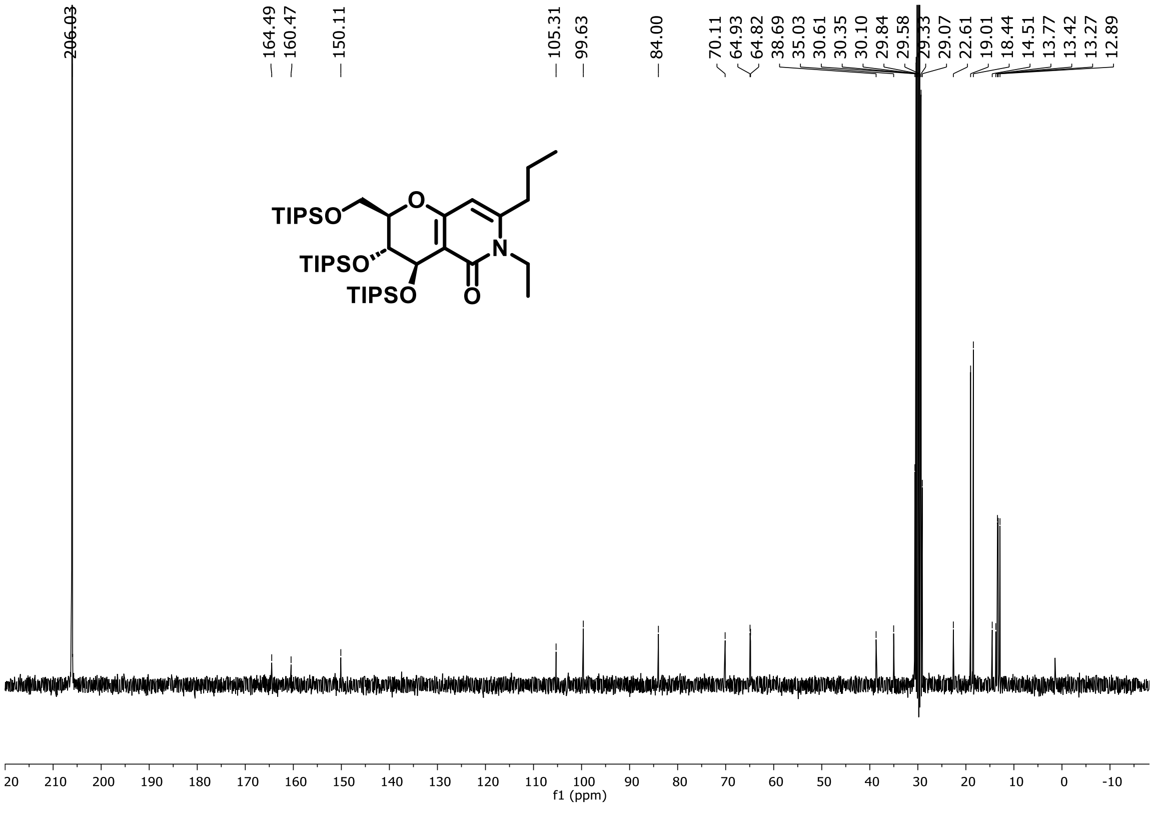

Figure S70. ^{13}C NMR spectra (75 MHz, $(CD_3)_2CO$) of **5j**

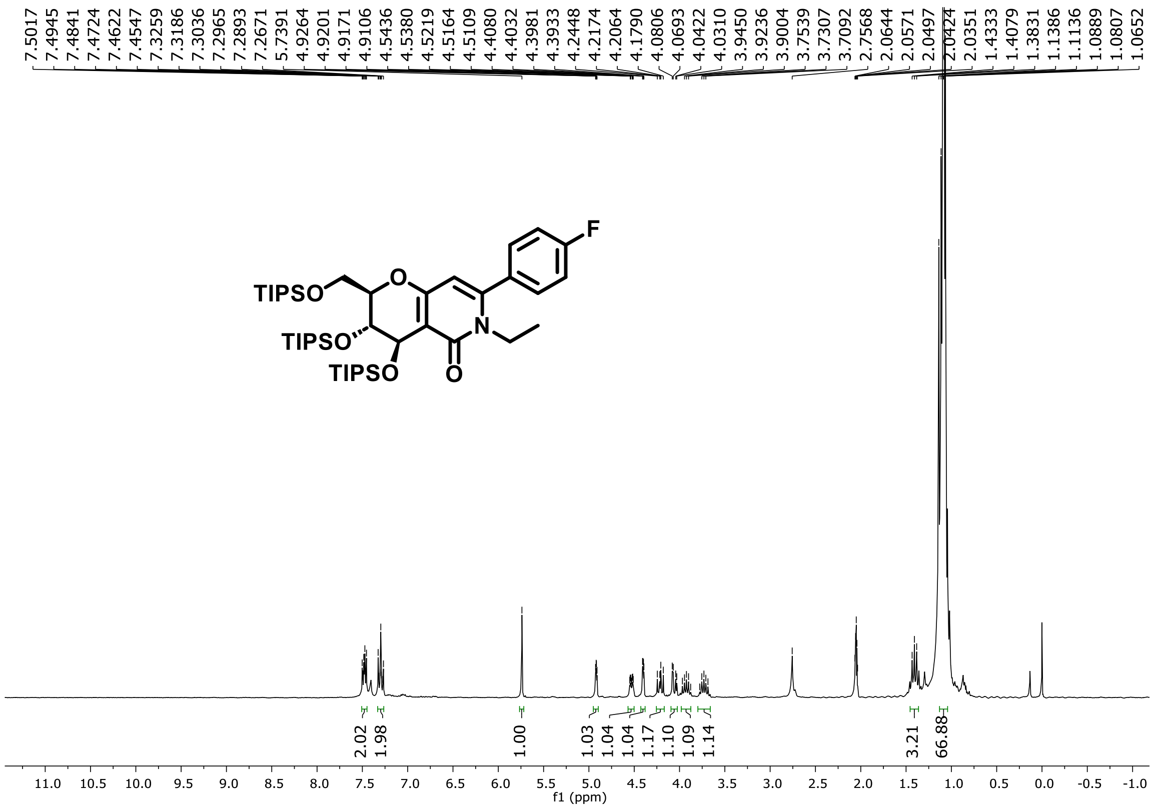

Figure S71. ^{1}H NMR spectra (300 MHz, $(CD_3)_2CO$) of **5k**

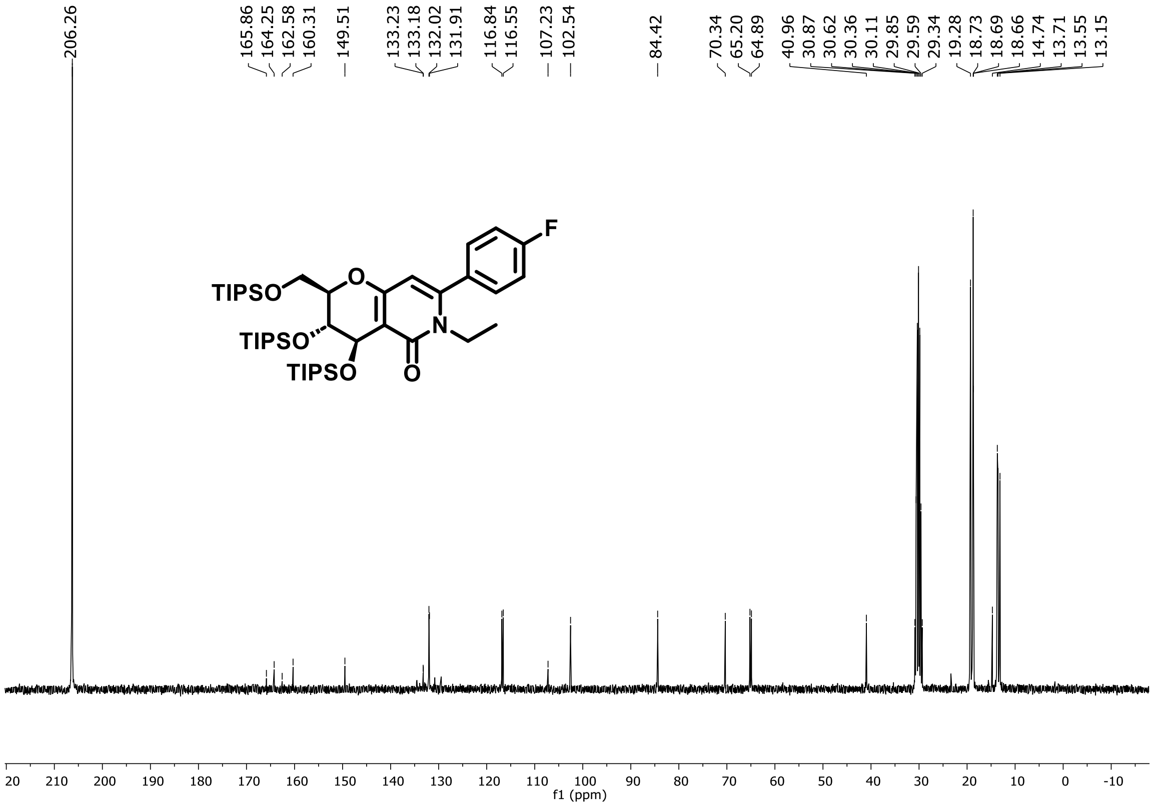

Figure S72. ^{13}C NMR spectra (75 MHz, $(CD_3)_2CO$) of **5k**

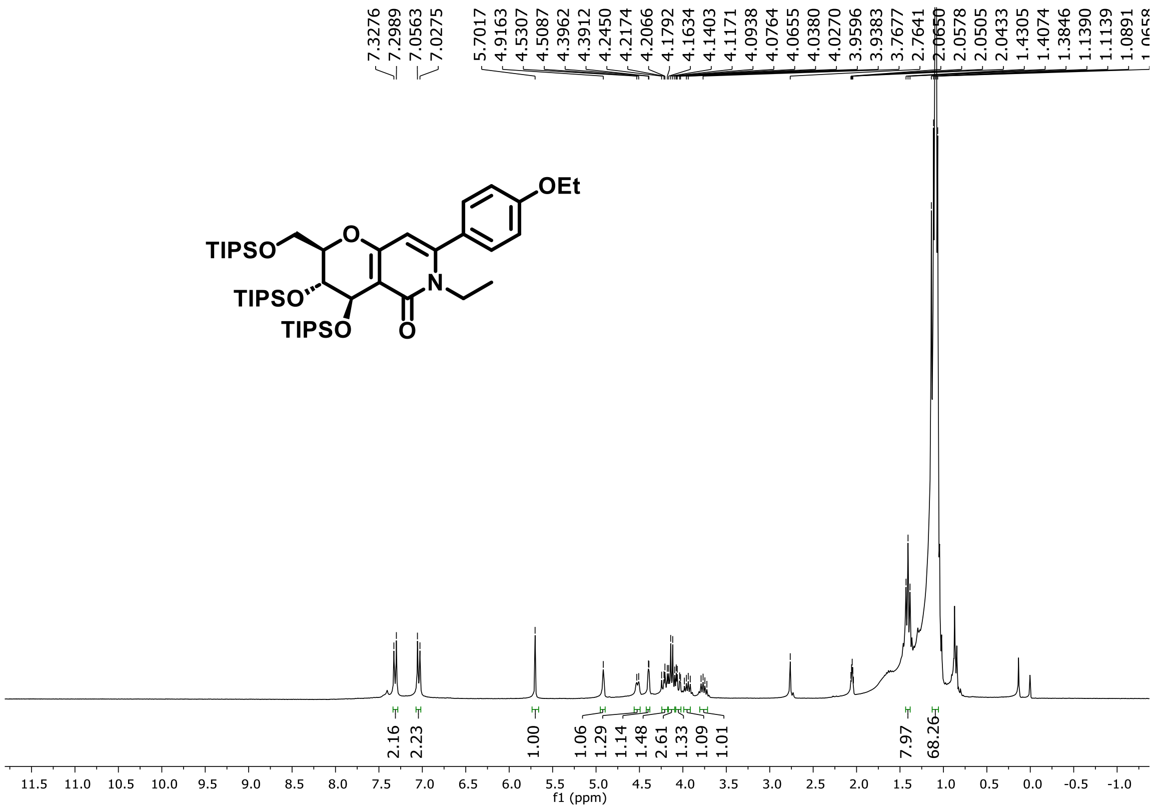

Figure S73. ^{1}H NMR spectra (300 MHz, $(CD_3)_2CO$) of **5l**

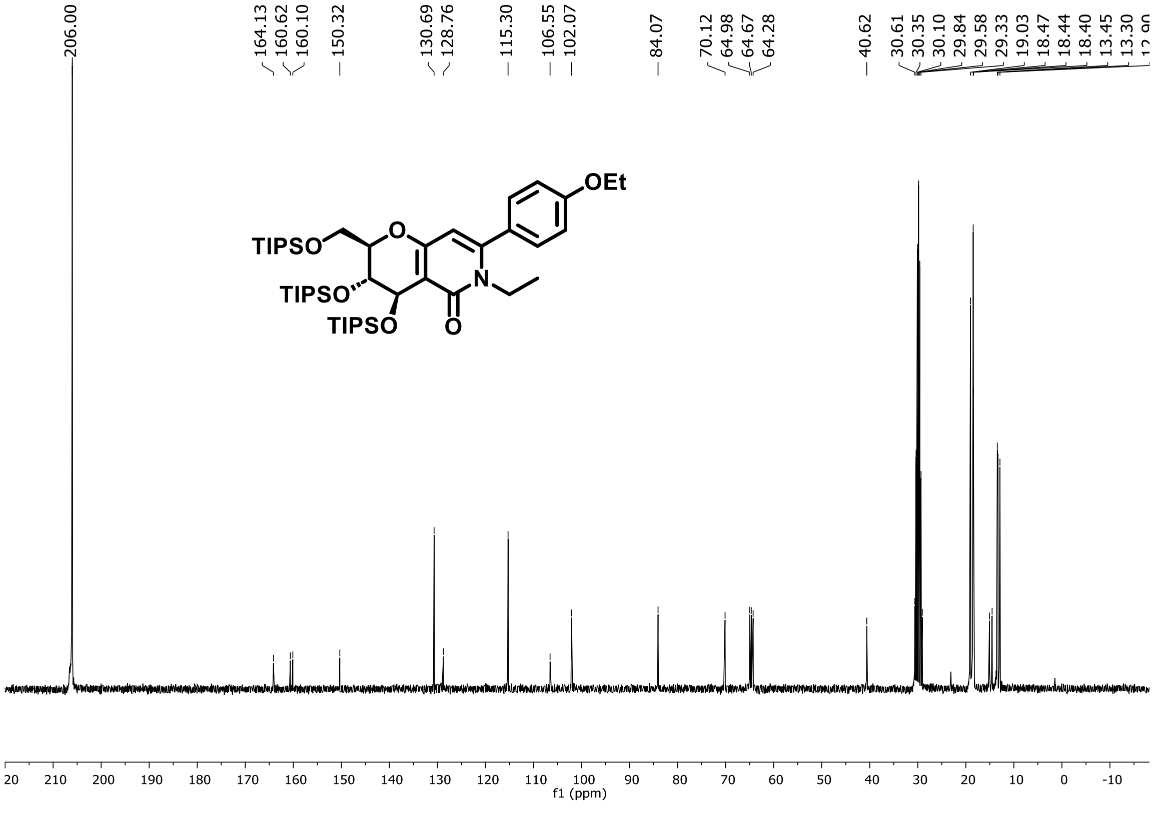

Figure S74. ^{13}C NMR spectra (75 MHz, $(CD_3)_2CO$) of **5l**

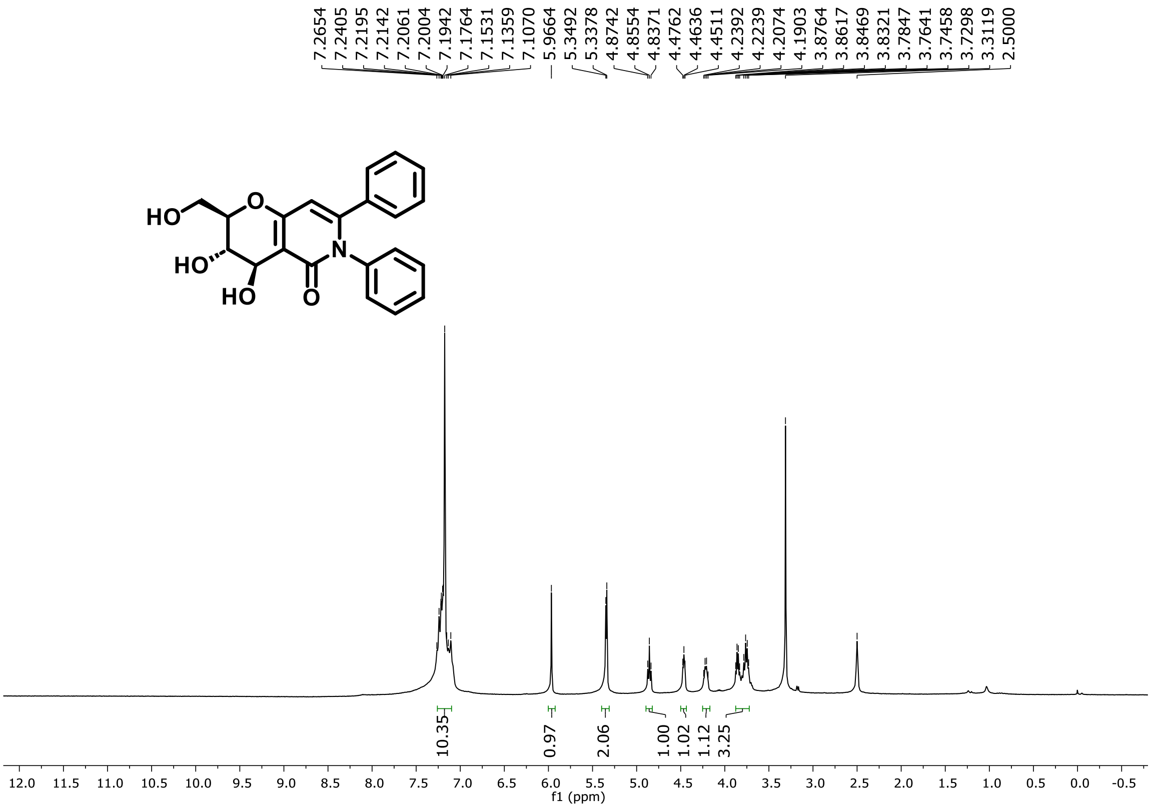

Figure S75. ^{1}H NMR spectra (300 MHz, DMSO-*d6*) of **6a**

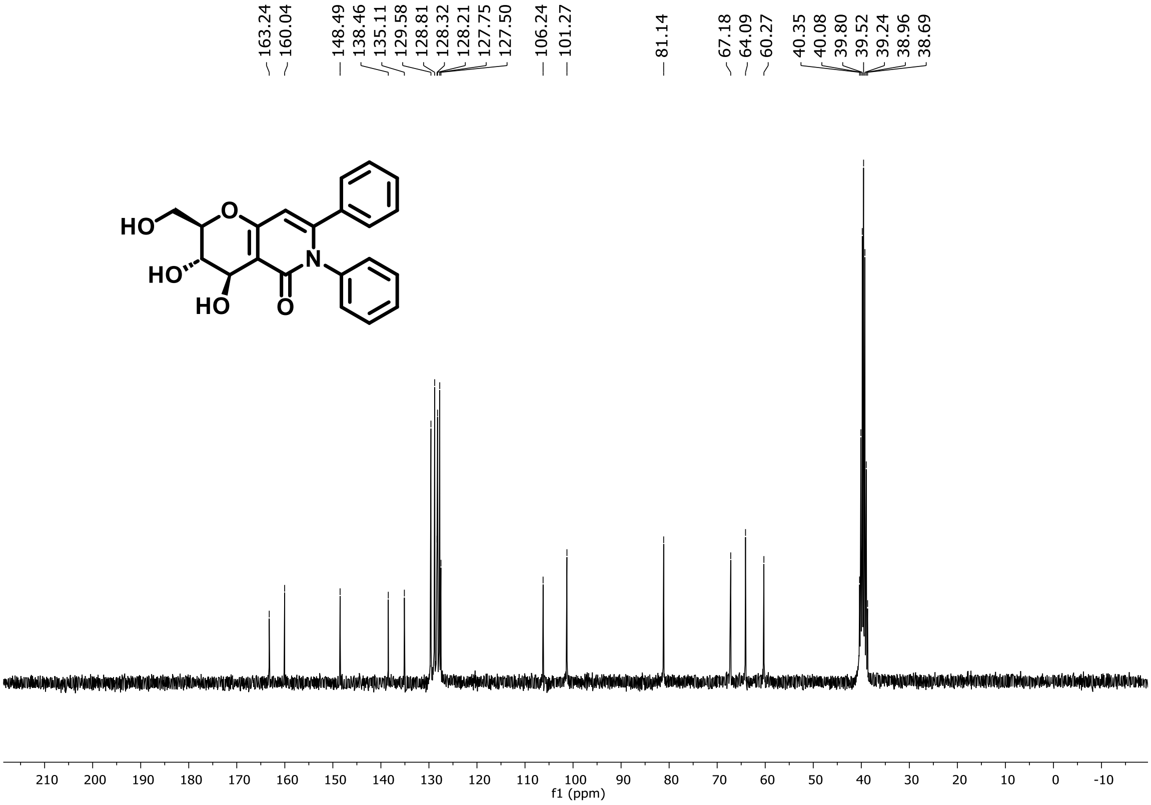

Figure S76. ^{13}C NMR spectra (75 MHz, DMSO-*d6*) of **6a**

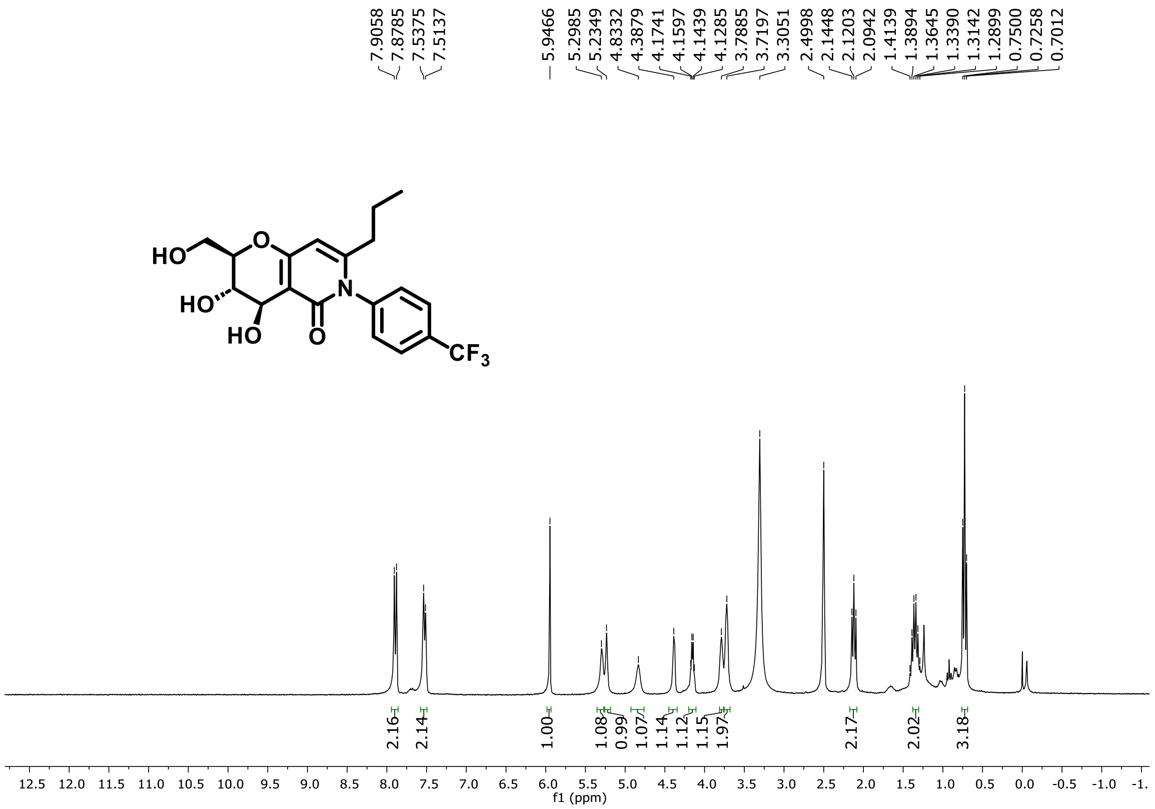

Figure S77. ^{1}H NMR spectra (300 MHz, DMSO-*d6*) of **6b**

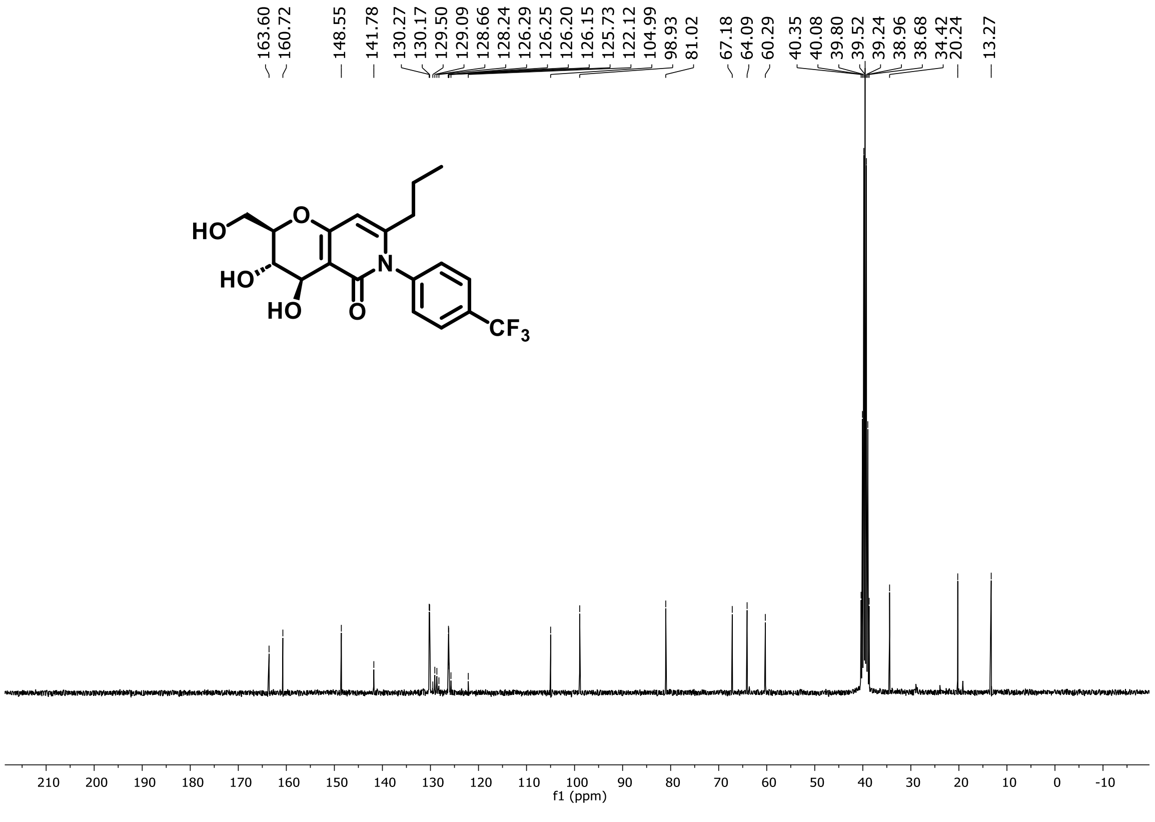

Figure S78. ^{13}C NMR spectra (75 MHz, DMSO-*d6*) of **6b**

4. NMR (HMBC, HSQC) Spectra of Products

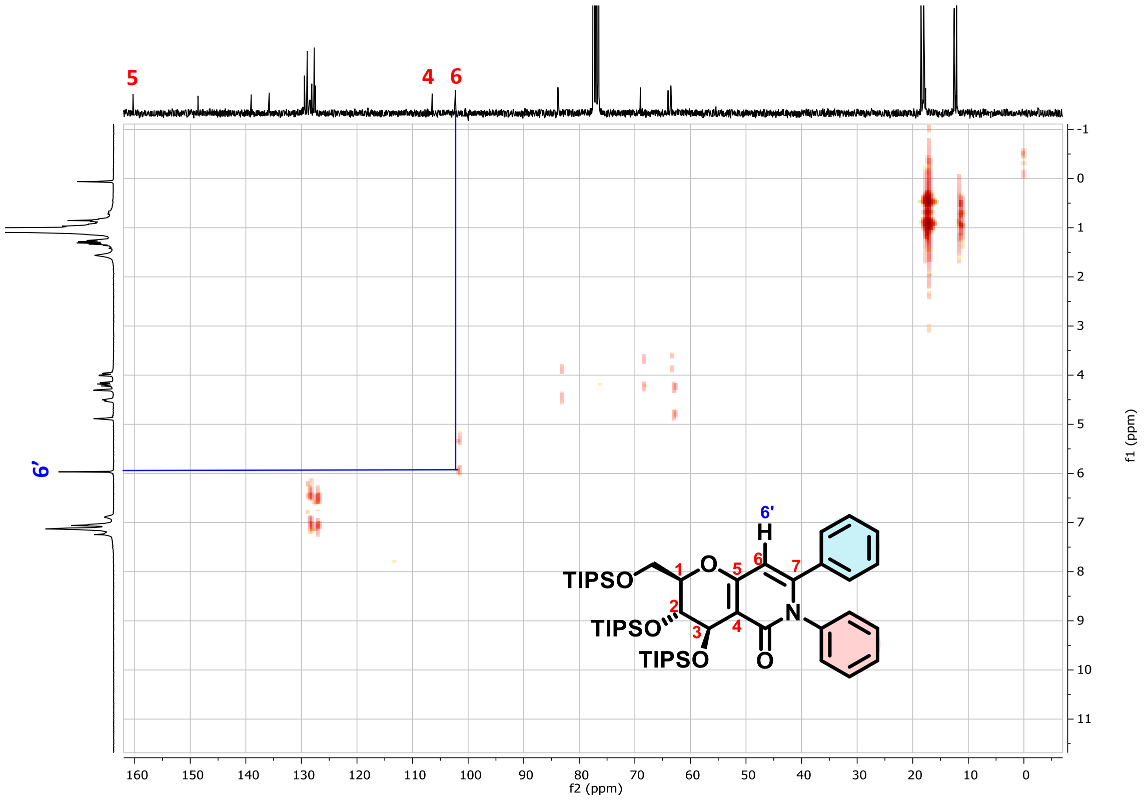

Figure S79. HSQC (300 MHz, $CDCl_3$) spectrum of product **4a.**

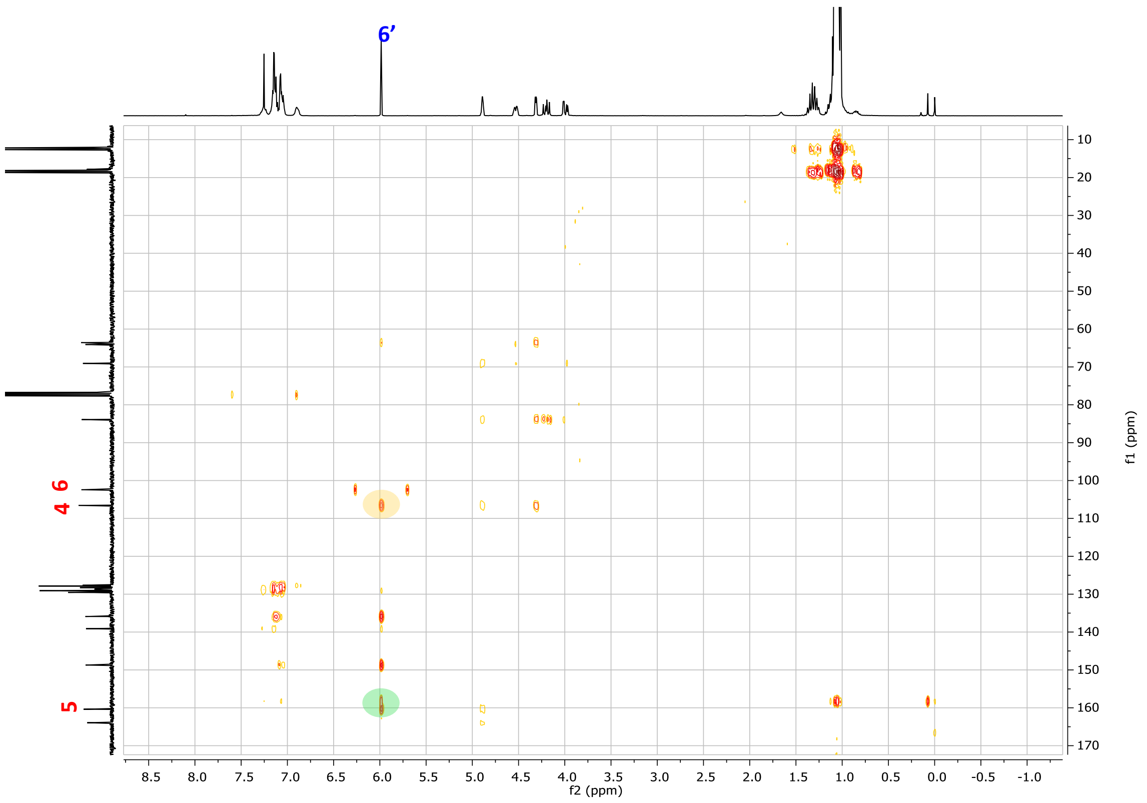

Figure S80. HMBC (300 MHz, $CDCl_3$) spectrum of product **4a**

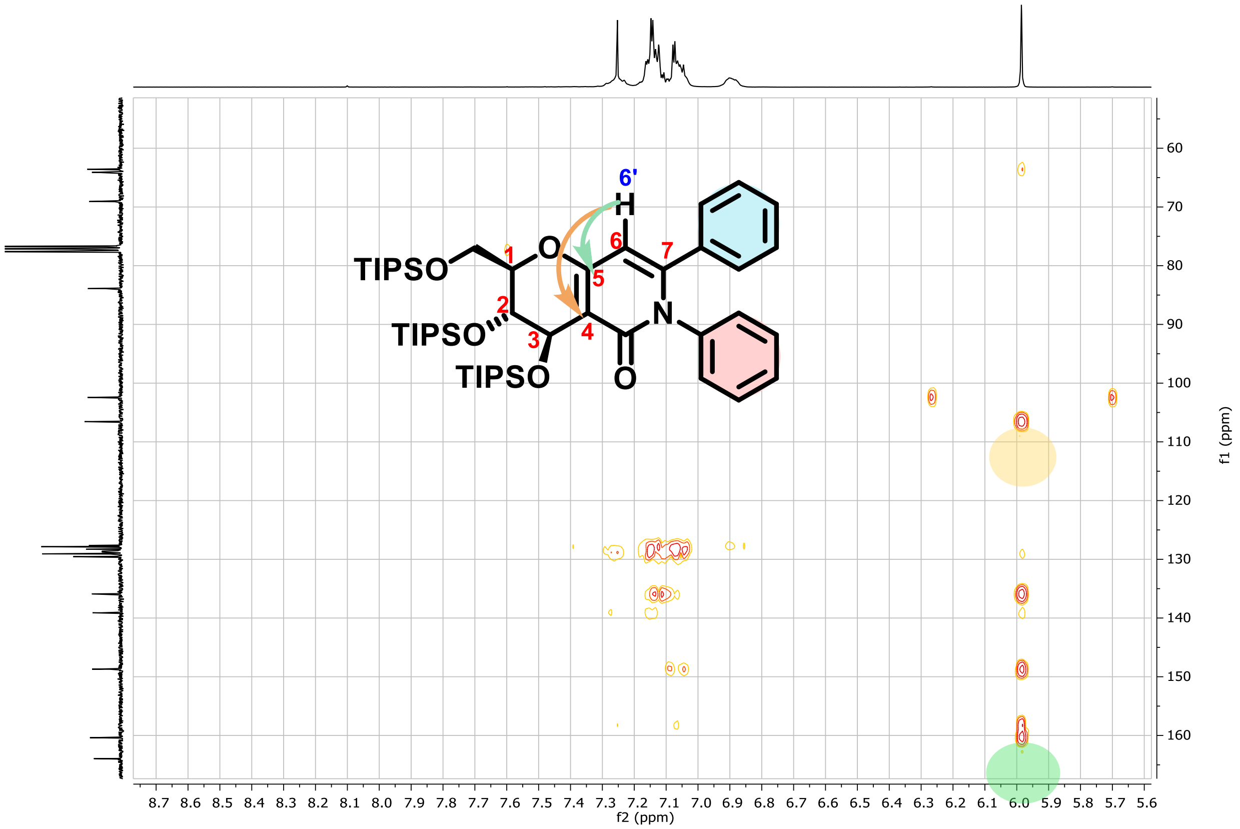

Figure S81. HMBC (300 MHz, $CDCl_3$) zoomed spectrum of product **4a** showing key **H6'- C5 $^2J_{CH}$ and H6'-C4 $^3J_{CH}$**
